# Effect of Internet-Delivered Emotion Regulation Individual Therapy for Adolescents With Nonsuicidal Self-Injury Disorder: A Randomized Clinical Trial

**DOI:** 10.1001/jamanetworkopen.2023.22069

**Published:** 2023-07-13

**Authors:** Johan Bjureberg, Olivia Ojala, Hugo Hesser, Henrike Häbel, Hanna Sahlin, Kim L. Gratz, Matthew T. Tull, Emma Claesdotter Knutsson, Erik Hedman-Lagerlöf, Brjánn Ljótsson, Clara Hellner

**Affiliations:** 1 Department of Clinical Neuroscience, Centre for Psychiatry Research, Karolinska Institutet, & Stockholm Health Care Services, Region Stockholm, Stockholm, Sweden; 2 School of Law, Psychology and Social Work, Örebro University, Örebro, Sweden; 3 Division of Biostatistics, Institute of Environmental Medicine, Karolinska Institutet, Stockholm, Sweden; 4 Department of Psychology, University of Toledo, Toledo, Ohio; 5 Lyra Health, Burlingame, California; 6 Department of Clinical Sciences Lund, Faculty of Medicine, Lund University, Lund, Sweden; 7 Department of Clinical Neuroscience, Division of Psychology, Karolinska Institutet, Stockholm, Sweden

## Abstract

**Importance:**

Nonsuicidal self-injury is prevalent in adolescence and associated with adverse clinical outcomes. Effective interventions that are brief, transportable, and scalable are lacking.

**Objective:**

To test the hypotheses that an internet-delivered emotion regulation individual therapy for adolescents delivered adjunctive to treatment as usual is superior to treatment as usual only in reducing nonsuicidal self-injury and that improvements in emotion regulation mediate these treatment effects.

**Design, Setting, and Participants:**

This 3-site, single-masked, randomized superiority trial enrolled participants from November 20, 2017, to April 9, 2020. Eligible participants were aged between 13 and 17 years and met diagnostic criteria for nonsuicidal self-injury disorder; they were enrolled as a mixed cohort of consecutive patients and volunteers. Parents participated in parallel to their children. The primary end point was at 1 month after treatment. Participants were followed up at 3 months posttreatment. Data collection ended in January 2021.

**Interventions:**

Twelve weeks of therapist-guided, internet-delivered emotion regulation individual therapy delivered adjunctive to treatment as usual vs treatment as usual only.

**Main Outcomes and Measures:**

Primary outcome was the youth version of the Deliberate Self-harm Inventory, both self-reported by participants prior to treatment, once every week during treatment, and for 4 weeks posttreatment, and clinician-rated by masked assessors prior to treatment and at 1 and 3 months posttreatment.

**Results:**

A total of 166 adolescents (mean [SD] age, 15.0 [1.2] years; 154 [92.8%] female) were randomized to internet-delivered emotion regulation therapy plus treatment as usual (84 participants) or treatment as usual only (82 participants). The experimental intervention was superior to the control condition in reducing clinician-rated nonsuicidal self-injury (82% vs 47% reduction; incidence rate ratio, 0.34; 95% CI, 0.20-0.57) from pretreatment to 1-month posttreatment. These results were maintained at 3-month posttreatment. Improvements in emotion dysregulation mediated improvements in self-injury during treatment.

**Conclusions and Relevance:**

In this randomized clinical trial, a 12-week, therapist-guided, internet-delivered emotion regulation therapy delivered adjunctive to treatment as usual was efficacious in reducing self-injury, and mediation analysis supported the theorized role of emotion regulation as the mechanism of change in this treatment. This treatment may increase availability of evidence-based psychological treatments for adolescents with nonsuicidal self-injury.

**Trial Registration:**

ClinicalTrials.gov Identifier: NCT03353961

## Key Points

**Question:**

What are the efficacy and mechanisms of change of a therapist-guided, internet-delivered emotion regulation therapy for nonsuicidal self-injury among adolescents?

**Findings:**

In this randomized clinical trial that included 166 adolescents, internet-delivered emotion regulation individual therapy delivered adjunctive to treatment as usual resulted in an 82% reduction in masked assessor-rated nonsuicidal self-injury frequency (vs a 47% reduction in treatment as usual only), a statistically significant difference. Improvements in emotion regulation mediated improvements in nonsuicidal self-injury.

**Meaning:**

A therapist-guided emotion regulation treatment delivered online may overcome common treatment barriers and increase availability of evidence-based psychological treatments for adolescents with nonsuicidal self-injury.

## Teaser

This randomized clinical trial examines the effectiveness of internet-delivered emotion regulation individual therapy in addition to usual care for reducing nonsuicidal self-injury among adolescents in Sweden.

## Introduction

Nonsuicidal self-injury (NSSI), the deliberate destruction of one’s body tissue without suicidal intent,^1^ is one of the strongest risk factors for suicide attempts and associated with a range of adverse outcomes.^2,3,4,5^ Indeed, NSSI disorder (NSSID) was included as a condition for further study in the *Diagnostic and Statistical Manual of Mental Disorders* (Fifth Edition).^6^ It is estimated that 3% to 7% of adolescents meet criteria for NSSID.^7^

There are very few empirically supported treatments for NSSI in adolescence.^8,9,10^ Dialectical behavior therapy is promising^8,9^; however, it is a long and resource-demanding treatment^11,12^ that is not widely available.^13^ Briefer and more accessible treatments for NSSI are needed.^8,9^

One way to treat NSSI more effectively is to target key mechanisms underlying NSSI.^10,14^ Emotion regulation has been identified as one of the most relevant mechanisms underlying this behavior, and a key mechanism of change in NSSI interventions.^8,9^ Open trial data provide support for the utility of a 12-week internet-delivered emotion regulation individual therapy for adolescents (IERITA) in the treatment of NSSI.^15,16,17^ Not only does this treatment aim to improve NSSI by directly targeting its underlying mechanism of emotion dysregulation, internet-delivered treatments with minimal clinician support have the potential to augment community therapy and increase availability of specialized psychological treatments.^18,19,20^

This study presents the results from what is to our knowledge the first randomized clinical trial of internet-delivered treatment for adolescents with NSSID. The first aim was to investigate the efficacy of therapist-guided IERITA delivered adjunctive to treatment as usual (TAU) compared with TAU only. The second aim was to examine emotion regulation as a mechanism of change in IERITA. We expected that IERITA plus TAU, relative to TAU only, would lead to both larger reductions in self-reported and assessor-rated NSSI frequency and greater improvements in the secondary outcomes of emotion dysregulation, global functioning, impulsive self-destructive behaviors, and psychiatric symptoms. Finally, we expected that improvements in emotion regulation would mediate improvements in NSSI during treatment.

## Methods

### Design

This was a 3-site, single-masked, randomized superiority trial comparing a 12-week, therapist-guided IERITA delivered adjunctive to TAU with TAU only for adolescents with NSSID and their parents. The trial was conducted within the Child and Adolescent Mental Health Services (CAMHS) in Stockholm, Västra Götaland, and Skåne in Sweden. Recruitment took place from November 20, 2017, to April 9, 2020. The primary end point was 1 month after treatment. Participants were followed up 3 months after treatment (ended January 2021). Participants randomized to TAU only were offered IERITA after the 3-month follow-up. We followed the Consolidated Standards of Reporting Trials (CONSORT) reporting guidelines. The trial was approved by the Stockholm Regional Ethical Review Board. All participants provided informed consent, with older participants providing written consent and younger participants verbal consent with parental written consent (eMethods in Supplement 1).

### Participants

Referrals from health care professionals and self-referrals from families living in Sweden were accepted. The trial was advertised in CAMHS, schools, and newspapers and on social media.

Inclusion criteria for the adolescents were: ages 13 to 17 years, meeting diagnostic criteria for NSSID, experiencing 1 or more NSSI episode during the past month, and having 1 guardian (hereafter referred to as parent) who could participate in the parent program. Exclusion criteria for the adolescents included: immediate suicide risk (ie, history of suicide attempt[s] [if triggers and/or life circumstances had not changed significantly since the attempt] or current suicide plans); diagnosis of psychotic or bipolar I disorder or ongoing (defined as past month) substance use disorder; other primary psychiatric disorder requiring immediate treatment (eg, severe anorexia nervosa); insufficient understanding of the Swedish language; life circumstances that could prevent treatment participation or that required immediate intervention; and a clinician-assessed global functioning level corresponding to a Children’s Global Assessment Scale (CGAS)^21^ score below 40.

### Procedures

Procedures for being admitted into the trial included an initial telephone screening, consecutive self-reported baseline assessments every 7 days for 4 weeks, and a face-to-face assessment at the clinic. During the face-to-face assessment, a psychologist or psychotherapist administered semi-structured interviews to assess for NSSID and other co-occurring psychiatric disorders (eMethods in Supplement 1).

Eligible participants were randomly allocated without stratification to IERITA plus TAU or TAU only. The random allocation sequence was based on a true random number service (Random.org) and conducted by an independent researcher in blocks of 4 or 6 per treatment clinic and placed in opaque sealed envelopes. Outcome measures included masked clinician-rated interviews (via telephone) and self-reported measures (via online platform). Psychologists and psychotherapists masked to treatment allocation and independent from the scientific team conducted the clinician-rated follow-up assessments. Details on masking and participant reimbursement are available in eMethods in Supplement 1.

### Outcomes

The primary outcome was self-reported and clinician-rated NSSI frequency as measured by the youth version of the Deliberate Self Harm Inventory (DSHI-Y).^22,23^ The DSHI-Y assesses the frequency of the 6 most common forms of NSSI (eg, cutting, burning). To optimize reliability and statistical power, the self-reported DSHI-Y, measuring past-week engagement in NSSI, was administered pretreatment, once every week during the treatment period, and for 4 weeks posttreatment (1-month posttreatment served as the primary end point). A clinician-rated version of the DSHI-Y measuring past-month NSSI was administered at pretreatment, 1-month posttreatment (primary end point), and 3-month posttreatment by masked assessors. Secondary outcomes included the masked clinician-rated assessment of global functioning (CGAS)^21^ and adolescent-reported emotion dysregulation (Difficulties in Emotion Regulation Scale [DERS]^24^ administered pretreatment and 1 and 3 months after treatment; and 16-item DERS [DERS-16]^25^ administered once every week during the treatment period, and for 4 weeks posttreatment), self-destructive behaviors (Borderline Symptom List [BSL]; details included in eMethods in Supplement 1),^26^ and psychiatric symptoms (Depression, Anxiety, and Stress Scales [DASS-21]).^27^ Self-reported measures were also administered to assess treatment credibility or expectancy, satisfaction, and adverse events in the IERITA plus TAU group. See eMethods and eTable 1 in Supplement 1 for information on measures and assessment points.

### Interventions

The therapist-guided IERITA used in this study had been optimized based on feasibility studies,^15,16,17^ and feedback from patients and end-user representatives. IERITA is a web-based, acceptance-based behavioral treatment that includes 11 modules for the adolescent participant and 6 modules for the parents delivered over the course of 12 weeks. The modules include text, animations, and interactive scripts (eFigures 1 through 3 in Supplement 1). The treatment on which IERITA is based, emotion regulation group therapy,^28^ was designed to treat NSSI by directly targeting its theorized underlying mechanism of emotion dysregulation. Thus, treatment modules aim to teach participants more adaptive ways of responding to their emotions (eTable 2 and eTable 3 in Supplement 1). A dedicated therapist (psychologist or psychotherapist) provided asynchronous online contact with both adolescent and parent (separately) via a message function in the platform. The therapist reinforced treatment engagement, answered questions, and assisted with homework assignments and problem solving. A mobile app was used to complement the adolescents’ internet-delivered treatment modules (eMethods; eFigure 4 in Supplement 1).

The comparison condition TAU was chosen to control for some nonspecific aspects of treatment (eg, time, monitoring) and ensure patient safety. The TAU was delivered in both conditions within regular CAMHS care by community clinicians (external to the research group) and typically involved supportive therapy sessions every 2 weeks. Thus, participants in IERITA plus TAU typically had contact with 2 clinicians (face-to-face contact with a community clinician and online contact with the IERITA therapist). TAU was enhanced by weekly self-rated assessments and as needed follow-ups from the research team (when deterioration in mental health was detected) (eMethods in Supplement 1).

### Statistical Analysis

See eMethods in Supplement 1 for descriptions of handling of outliers and missing data, the data visualization procedure, and computation methods for effect sizes. Based on an expected average difference of 2 NSSI episodes between the conditions at 1-month posttreatment, power analysis revealed a needed sample size of 166 participants with a power of 82%, α = 0.05, and a maximum attrition rate of 15% (eMethods in Supplement 1 and Trial Protocol in Supplement 2).

Treatment effects were evaluated according to the intention-to-treat principle. The primary outcome analysis of self-reported NSSI included treatment condition and weekly self-reports of NSSI frequency (using the DSHI-Y). A zero-inflated negative binomial generalized linear-mixed effects regression model was used to estimate the trend over time for self-reported NSSI counts measured every week by including an interaction term between treatment condition and time while allowing for a random intercept and slope per individual. Treatment condition and study site were included as categorical variables and time (weeks zero to 16) was treated as a continuous variable. Exploratory analyses investigated whether certain relevant TAU or client characteristics moderated treatment effects (eMethods in Supplement 1).

For pretreatment and 1-month and 3-month posttreatment analyses, each assessment point was included as a dummy variable in separate mixed effects regression models including a random intercept, and an interaction term between treatment condition and the categorized time variable (pretreatment, 1-month posttreatment, 3-month posttreatment). Treatment condition and study site were included as categorical variables. For assessor-rated NSSI frequency (DSHI-Y), a zero-inflated negative binomial regression model was fitted to the NSSI counts. For counts on other self-destructive behaviors (BSL-Supplement), zero-inflated Poisson regression models were used. Model comparisons are available in eTable 10 in Supplement 1. All other outcomes were analyzed in a similar fashion, but with linear mixed-effects models assuming a normal distribution of the residuals.

To determine whether change in week-to-week emotion dysregulation (DERS-16) during treatment mediated the overall effect of IERITA on week-to-week change in the self-reported NSSI frequency, we employed a parallel process latent growth curve modeling approach for mediation.^29^ A detailed description of the mediation analysis, including sensitivity analyses that examined the impact of unmeasured pretreatment mediator-outcome confounding, is available in eMethods in Supplement 1.^30^ Mixed-effect regression analyses for repeated measures (including all assessment points) are valid under the assumption that the data are missing at random (eMethods in Supplement 1).^31^ All tests were 2-sided, analyzed using R version 4.1.0 (R Core Team) and Mplus version 8.1 (Muthén & Muthén), and statistical significance was set at *P* < .05.

## Results

**Figure 1.**
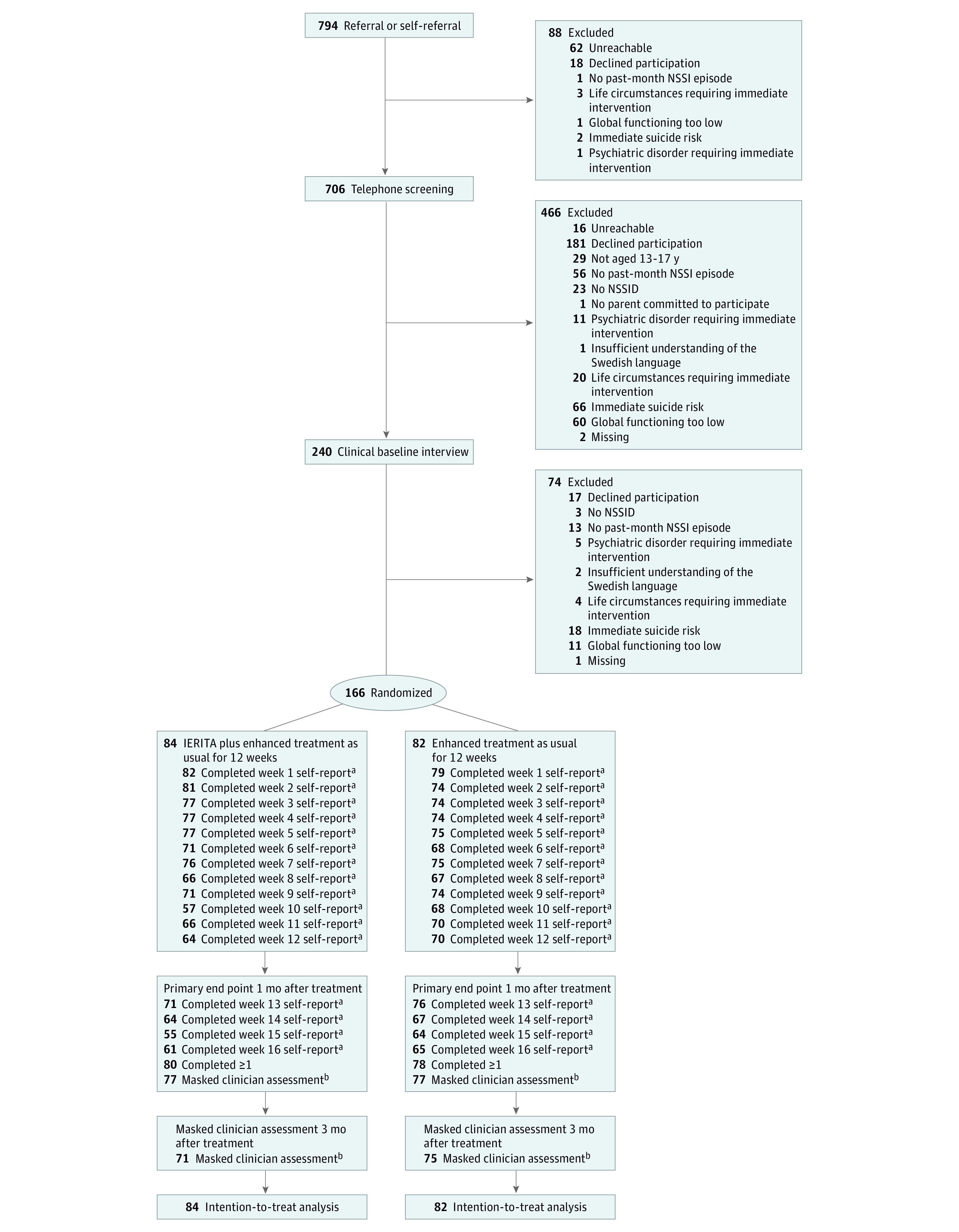
Flow Diagram of Patient Enrollment and Disposition IERITA indicates internet-delivered emotion regulation individual therapy for adolescents; NSSID, nonsuicidal self-injury disorder. ^a^Self-reported Deliberate Self-Harm Inventory–Youth Version. ^b^Masked Assessor-rated Deliberate Self-Harm Inventory–Youth Version.

A total of 166 participants (mean [SD] age, 15.0 [1.2] years; 154 [92.8%] female) were recruited and randomly allocated to IERITA plus TAU (84 participants) or TAU only (82 participants) (Table 1). Twelve participants (7%; 5 in IERITA plus TAU, 7 in TAU only) dropped out of the treatment (Figure 1). The majority (9 participants) dropped out between the first and sixth week of treatment. Twelve participants (7%; 7 in IERITA plus TAU, 5 in TAU only) and 20 (12%; 13 in IERITA plus TAU; 7 in TAU only) did not complete the clinician assessments at 1 month or 3 months after treatment, respectively. Eight participants (5%; 4 in each condition) did not complete any of the self-reported measures of NSSI during the 4 weeks following treatment completion (4 assessments total).

**Table 1.** Study and Participant Characteristics

Characteristics	Participants, No. (%)		
	IERITA plus TAU (n = 84)	TAU only (n = 82)	Total (n = 166)
**Study characteristics**			
Site			
Skåne	38 (45)	35 (43)	73 (44)
Stockholm	28 (33)	24 (30)	52 (31)
Västra Götaland	18 (21)	23 (28)	41 (25)
Source of referral			
Clinician	55 (66)	46 (56)	101 (61)
Self	29 (35)	36 (44)	65 (39)
**Participant characteristics**			
Gender			
Female	77 (92)	77 (94)	154 (93)
Male	5 (6)	2 (2)	7 (4)
Nonbinary	2 (2)	3 (4)	5 (3)
Age, mean (SD), y	15.04 (1.31)	15.02 (1.19)	15.03 (1.25)
Sexual orientation			
Heterosexual	56 (67)	55 (67)	111 (67)
Sexual minority	26 (31)	26 (33)	52 (31)
No answer	2 (2)	1 (1)	3 (2)
Region of birth			
Sweden	80 (95)	80 (98)	160 (96)
Asia, South or North America, or Europe	4 (5)	2 (2)	6 (4)
Any failed grades	11 (13)	16 (20)	27 (16)
**Parent characteristics^a^**			
Parent living arrangement			
With children	67 (80)	67 (82)	134 (81)
With spouse or partner	58 (70)	63 (77)	121 (73)
Alone	4 (5)	2 (2)	6 (4)
Parent education level			
Primary school	1 (1)	2 (2)	3 (2)
Secondary school	35 (42)	31 (38)	66 (40)
College or university <3 y	7 (8)	8 (10)	15 (9)
College or university ≥3 y	35 (42)	37 (45)	72 (43)
Doctorate	6 (7)	4 (5)	10 (6)
Parent occupational status			
Employed or self-employed	82 (98)	77 (94)	159 (96)
Unemployed, sick leave, or retired	2 (2)	5 (6)	7 (4)
**Participant clinical characteristics**			
Age NSSI onset, mean (SD), y	12.70 (1.27)	12.51 (1.57)	12.61 (1.42)
Time from NSSI onset, mean (SD), y	2.33 (1.36)	2.51 (1.31)	2.42 (1.33)
Comorbidity^b^			
Major depressive disorder	49 (58)	48 (59)	97 (58)
Anxiety disorders			
Social anxiety disorder	24 (29)	23 (28)	47 (28)
Panic disorder or agoraphobia	17 (20)	11 (13)	28 (17)
Specific phobia disorder	14 (17)	13 (16)	27 (16)
Generalized anxiety disorder	12 (14)	9 (11)	21 (13)
ADHD^c^	14 (17)	15 (18)	29 (18)
Autism spectrum disorder	4 (5)	3 (4)	7 (4)
OCD or BDD	3 (4)	7 (9)	10 (6)
Eating disorder^d^	6 (7)	1 (1)	7 (4)
Oppositional defiant disorder	3 (4)	2 (2)	5 (3)
No. of co-occurring disorders, mean (SD)	1.93 (1.76)	1.85 (1.52)	1.89 (1.64)
No. of BPD criteria, mean (SD)^e^	1.88 (1.28)	2.11 (1.54)	1.99 (1.42)
Fulfilling ≥5 BPD criteria	5 (6)	7 (9)	12 (7.2)
Suicidality			
Low	37 (44)	37 (45)	74 (45)
Moderate	21 (25)	21 (26)	42 (25)
High	26 (31)	24 (29)	50 (30)
Any previous suicide attempt^f^	13 (16)	12 (15)	25 (15)
Ever received inpatient care	2 (2)	2 (2)	4 (2)
Previous counselling	54 (64)	53 (65)	107 (64)
Time in previous counselling, mean (SD), mo	10.6 (13.8)	10.2 (14.8)	10.4 (14.3)
Any ongoing psychopharmacological medication^g^	31 (37)	25 (31)	56 (34)
Antidepressants (N06A)	20 (24)	15 (18)	35 (21)
SSRI (N06AB)	20 (24)	15 (18)	35 (21)
Other antidepressants (N06AX)	0	1 (1)	1 (1)
Anxiolytics (N05B)	6 (7)	8 (10)	14 (8)
Diphenylmethane derivatives (N05BB)	6 (7)	8 (10)	0
Benzodiazepine (N05BA)	0	1 (1)	0
Hypnotics and sedatives (N05C)	19 (23)	12 (15)	31 (19)
Melatonin receptor agonists (N05CH)	18 (21)	11 (13)	29 (17)
Other hypnotics and sedatives (N05CM)	1 (1)	1 (1)	2 (1)
Antihistamines for systemic use (R06A)	11 (13)	8 (10)	19 (11)
Psychostimulants (N06B)	4 (5)	6 (7)	10 (6)
Antiadrenergic agents (C02A)	0	1 (1)	1 (1)
Antipsychotics (N05A)	1 (1)	0 (0)	1 (1)
Ongoing counselling at inclusion	61 (73)	55 (67)	116 (67)
Time in ongoing counselling, mean (SD), mo	5.6 (5.9)	5.3 (6.3)	5.5 (6.1)
Recent onset (≤2 mo) of ongoing counselling	23 (27)	20 (24)	43 (26)
Type of ongoing counselling			
Supportive therapy	23 (38)	26 (47)	49 (42)
CBT	22 (36)	16 (29)	38 (33)
Do not know	14 (23)	12 (22)	26 (22)
PDT	2 (3)	1 (2)	3 (3)

Abbreviations: ADHD, attention-deficit hyperactivity disorder; BDD, body dysmorphic disorder; BPD, borderline personality disorder; CBT, cognitive behavioral therapy; IERITA, internet-delivered emotion regulation individual therapy for adolescents; TAU, enhanced treatment as usual; NSSI, nonsuicidal self-injury; OCD, obsessive-compulsive disorder; PDT, psychodynamic therapy; SSRI, selective serotonin reuptake inhibitors.

^a^ In case of 2 parents, 1 was assigned and consented to contribute to answer self-reports questions. Multiple answers were allowed.

^b^ Assessed by the research team using the MINI-KID (Mini-International Neuropsychiatric Interview for Children and Adolescents) version 6 and the Body Dysmorphic Disorder Questionnaire (administered as an interview).

^c^ Includes both combined, primarily inattentive, and primarily hyperactive-impulsive subtype.

^d^ Includes anorexia nervosa and bulimia nervosa.

^e^ Assessed by the research team using the Structured Clinical Interview for *Diagnostic and Statistical Manual of Mental Disorders* (Fifth Edition).

^f^ In total 8 participants (5%) had missing value on this variable.

^g^ Classes of psychopharmacological medication were based on World Health Organization anatomic therapeutic chemical categories. See eTable 13 in Supplement 1 for a breakdown of substances included in each group.

Type and level of TAU (counseling and psychopharmacology) did not differ across conditions during the trial (Table 2). In addition to the TAU received at CAMHS, the study therapists spent a mean (SD) of 26.9 (35.9) minutes per family on phone calls with participants enrolled in TAU only.

**Table 2.** Characteristics of Enhanced Treatment as Usual Within Each Condition and Differences Between Conditions^a^

Characteristic	Participants, No. (%)		χ^2^	*P* value
	IERITA plus TAU (n = 79)	TAU only (n = 76)		
**Pretreatment to 1-mo posttreatment**				
Received counselling	62 (78.5)	56 (73.7)	0.490	.48
Counselling type				
Supportive therapy	48 (77.4)	47 (83.9)	0.795	.37
CBT	6 (9.7)	5 (8.9)	0.020	.89
Do not know	8 (10.1)	4 (5.2)	1.069	.30
Frequency				
Every week	11 (17.8)	13 (23.2)	0.544	.46
Every second week	18 (29.0)	20 (35.7)	0.602	.44
1 mo	23 (37.1)	14 (25.0)	2.000	.16
<1 mo	9 (14.5)	9 (16.1)	0.055	.81
Never	1 (1.6)	0	0.911	.34
Inpatient care	0	1 (1.3)	NA	NA
Any ongoing psychopharmacological medication^b^	32 (46)	35 (41)	0.489	.49
Antidepressants (N06A)	24 (29)	21 (26)	0.184	.67
SSRI (N06AB)	24 (30)	20 (26)	0.315	.58
Other antidepressants (N06AX)	0	1 (1)	1.046	.31
Anxiolytics (N05B)	7 (9)	9 (12)	0.333	.56
Diphenylmethane (N05BB)	7 (9)	9 (12)	0.333	.56
Hypnotics and sedatives (N05C)	12 (14)	15 (18)	0.489	.48
Melatonin receptor agonists (N05CH)	11 (14)	14 (18)	0.579	.45
Other hypnotics and sedatives (N05CM)	1 (1)	2 (3)	0.381	.54
Antihistamines for systemic use (R06A)	9 (11)	5 (7)	1.092	.30
Psychostimulants (N06B)	5 (6)	6 (8)	0.144	.70
Antiadrenergic agents (C02A)	0	2 (3)	2.106	.15
Change in psychopharmacological medication	19 (24.1)	20 (25.3)	0.106	.75
Increased	12 (63.2)	12 (60.0)	0.041	.84
New	11 (57.9)	10 (50.0)	0.244	.62
Decreased	2 (10.5)	2 (10.0)	0.003	.96
Discontinue	3 (15.8)	3 (15.0)	0.005	.95
**1-mo posttreatment to 3-mo posttreatment (IERITA plus TAU, 74 participants; TAU only, 74 participants)** ^c^				
Counselling	48 (64.9)	52 (70.3)	0.493	.48
Counselling type				
Supportive therapy	35 (72.9)	37 (71.2)	0.039	.84
CBT	7 (14.6)	11 (21.2)	0.730	.39
Do not know	6 (8.1)	2 (2.7)	1.736	.19
Frequency				
Every week	7 (14.6)	12 (23.1)	1.170	.28
Every 2nd week	11 (22.9)	13 (25.0)	0.059	.81
1 mo	16 (33.3)	14 (26.9)	0.488	.49
<1 mo	14 (29.2)	13 (25.0)	0.222	.64
Never	0	0	NA	NA
Inpatient care	1 (1.4)	1 (1.4)	NA	NA
Any ongoing psychopharmacological medication^b^	35 (46)	31 (42)	0.263	.61
Antidepressants (N06A)	28 (33)	21 (26)	1.190	.28
SSRI (N06AB)	28 (37)	20 (27)	1.660	.20
Other antidepressants (N06AX)	0	1 (1)	1.034	.31
Anxiolytics (N05B)	2 (3)	6 (8)	2.227	.14
Diphenylmethane (N05BB)	2 (3)	6 (8)	2.227	.14
Hypnotics and sedatives (N05C)	18 (21)	14 (17)	0.506	.48
Melatonin receptor agonists (N05CH)	18 (24)	13 (18)	0.856	.36
Other hypnotics and sedatives (N05CM)	1 (1)	2 (3)	0.368	.54
Antihistamines for systemic use (R06A)	7 (9)	9 (12)	0.343	.56
Beta blocking beta agents (C07A)	1 (1)	0	0.980	.32
Antipsychotics (N05A)	1 (1)	0	0.982	.33
Antiepileptics (N03A)	1 (1)	1 (1)	0.000	.99
Change in psychopharmacological medication	17 (23.0)	16 (21.6)	0.039	.84
Increased	12 (70.6)	7 (43.8)	2.431	.12
New	7 (41.2)	8 (50.0)	0.259	.61
Decreased	4 (23.5)	5 (31.3)	0.248	.62
Discontinue	4 (23.5)	3 (18.8)	0.113	.74

Abbreviations: CBT, cognitive behavioral therapy; IERITA, internet-delivered emotion regulation therapy for adolescents; TAU, enhanced treatment as usual; SSRI, serotonin reuptake inhibitors.

^a^ Description of the level and content of enhanced treatment as usual that both conditions received during the trial.

^b^ Classes of psychopharmacological medication were based on World Health Organization anatomic therapeutic chemical categories (eTable 13 in Supplement 1).

^c^ Pharmalogical results included 76 participants in the IERITA plus TAU arm.

Ratings of treatment credibility, expectancy, and satisfaction with IERITA were moderate to high (eTable 11 in Supplement 1). Adolescents completed a mean (SD) of 9.6 modules out of 11 and parents completed 5.5 (1.0) modules out of 6 (similar for participants enrolled in treatment during the COVID-19 pandemic) (eResults in Supplement 1). In total, the mean (SD) therapist time for the IERITA intervention (including reviewing homework and providing feedback) was 378.8 (163.1) minutes per family.

**Figure 2.**
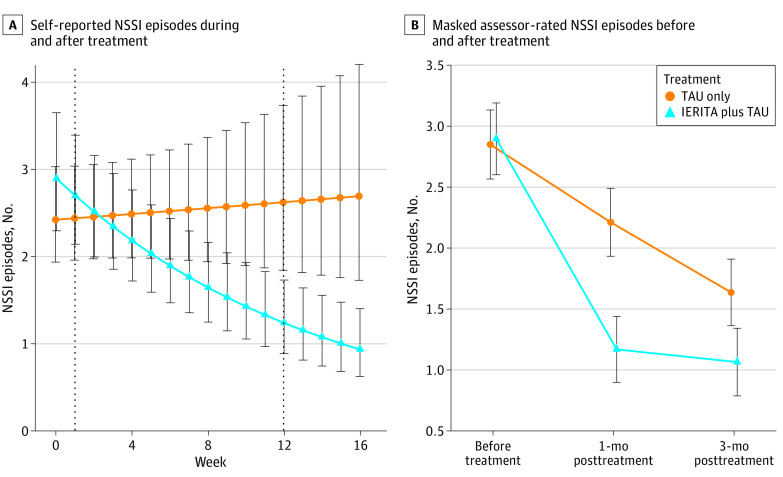
Self-Reported Nonsuicidal Self-Injury Disorder (NSSI) Episodes During Treatment and Posttreatment and Masked Assessor-Rated NSSI Episodes Pretreatment and Posttreatment Error bars indicate 95% confidence intervals.

Reduction in self-reported NSSI frequency (DSHI-Y) was larger in IERITA plus TAU vs TAU only from pretreatment to 1-month posttreatment (β [SE], −0.08 [0.02]; *P* < .001; IRR, 0.29; 95% CI, 0.14-0.58) (Figure 2, panel A). Participants in IERITA reported reductions in NSSI frequency of 68% (IRR, 0.32; 95% CI, 0.17-0.60) from pretreatment to 1-month posttreatment; there was no improvement of participants in TAU only during the same period (IRR, 1.11; 95% CI, 0.62-1.99) (eTable 12 in Supplement 1). Change in NSSI frequency was not associated with the examined moderators (ie, TAU or client characteristics) (eResults in Supplement 1).

Table 3 presents the results for masked assessor-rated NSSI frequency (as well as the secondary outcome measures). IERITA plus TAU led to greater reductions than TAU only in assessor-rated NSSI (DSHI-Y) (β [SE], −1.09 [0.27]; *P* < .001; IRR, 0.34; 95% CI, 0.20-0.57). At 1-month posttreatment, participants in IERITA evidenced an 82% reduction in NSSI frequency (IRR, 0.18; 95% CI, 0.09-0.36), whereas those in TAU only evidenced a 47% reduction (IRR, 0.53; 95% CI, 0.26-1.07). At 3-month posttreatment, IERITA plus TAU still showed a superior reduction in NSSI frequency than TAU only (β [SE], −0.62 [0.29]; *P* = .04; IRR, 0.54; 95% CI, 0.30-0.96) (Figure 2, panel B). See eResults in Supplement 1 for post hoc analyses on absence of NSSI at follow-up.

**Table 3.** Results From the Generalized and Linear Mixed Model Analyses and Effect Sizes^a^

Measurement	IERITA plus TAU		TAU only		Fixed effects		Effect size (95% CI)
	Result	Participants, No.^b^	Result	Participants, No.^b^	β (SE)	*P* value	
**Masked assessor-rated NSSI (DSHI-Y), median (IQR)** ^c^							
Pretreatment	8 (4-17)	84	8 (5-17)	82	NA	NA	NA
1-mo posttreatment	0 (0-4)	77	3 (0-11)	77	−1.09 (0.27)	<.001	0.34 (0.20 to 0.57)^d^
3-mo posttreatment	1 (0-5)	71	1 (0-6)	75	−0.62 (0.30)	.04	0.54 (0.30 to 0.62)^d^
**Self-reported self-destructive behaviors (BSL-Supplement), median (IQR)** ^e^							
Pretreatment	2 (1 to 4)	NA	2 (1 to 4)	82	NA	NA	NA
1-mo posttreatment	0 (0 to 1)	61	1 (0 to 2)	6482 65	−0.77 (0.23)	<.001	0.46 (0.28 to 0.77)^d^
3-mo posttreatment	0 (0 to 2)	73	1 (0 to 2)	75	−0.62 (0.13)	<.001	0.54 (0.33 to 0.87)^d^
**Masked assessor-rated global functioning (CGAS), mean (SD)** ^f^							
Pretreatment	54.30 (5.56)	84	54.84 (6.78)	82	NA	NA	NA
1-mo posttreatment	59.17 (8.93)	81	57.19 (8.36)	78	2.66 (1.1)	.02	0.43 (0.10 to 0.79)^g^
3-mo posttreatment	61.41 (9.18)	74	59.08 (8.56)	76	2.97 (1.13)	.009	0.48 (0.13 to 0.87)^g^
**Self-reported emotion dysregulation (DERS), mean (SD)** ^f^							
Pretreatment	128.35 (20.29)	84	130.17 (19.42)	82	NA	NA	NA
1-mo posttreatment	111.38 (26.38)	80	123.62 (25.15)	78	−10.27 (3.19)	<.001	0.52 (0.14 to 0.89)^g^
3-mo posttreatment	107.36 (28.59)	74	117.25 (29.70)	72	−8.13 (3.28)	.01	0.41 (0.09 to 0.73)^g^
**Self-reported psychiatric symptoms (DASS-21), mean (SD)** ^f^							
Pretreatment	31.08 (11.55)	84	32.24 (11.81)	82	NA	NA	NA
1-mo posttreatment	27.56 (12.1-28)	80	30.46 (13.89)	78	−1.87 (1.79)	.23	0.16 (−0.15 to 0.41)^g^
3-mo posttreatment	25.78 (14.38)	73	27.81 (15.17)	72	−1.37 (1.84)	.46	0.12 (−0.17 to 0.37)^g^

Abbreviations: BSL, Borderline Symptom List; CGAS, Children’s Global Assessment Scale; *d*, Cohen d; DASS-21, Depression Anxiety Stress Scales, 21-item version; DERS, Difficulties in Emotion Regulation Scale; DSHI-Y, Deliberate Self-Harm Inventory–Youth Version; IERITA, internet-delivered emotion regulation individual therapy; IRR incidence rate ratio; NSSI, nonsuicidal self-injury; TAU, enhanced treatment as usual.

^a^ Of count and continuous outcomes evaluating treatment differences in change from pretreatment to 1-month posttreatment (primary end point) and 3-month posttreatment (secondary end point). Fixed-effects parameter estimates β (SE) represent the time × group interaction with all randomized individuals (166 total). Positive effect sizes IRR and *d* indicate results favoring IERITA. Medians (IQR) and means (SDs) are observed values.

^b^ Numbers of participants contributing with data.

^c^ This outcome was analyzed using a zero-inflated negative binomial regression model.

^d^ Effect size result is an IRR.

^e^ This outcome was analyzed using a zero-inflated Poisson regression model.

^f^ This outcome was analyzed using a linear mixed effects regression model assuming a normal distribution.

^g^ Effect size result given as *d*.

Compared with TAU only, adolescents in IERITA plus TAU had larger improvements in self-reported emotion dysregulation (DERS: *d* = 0.52; 95% CI, 0.14-0.89), self-reported self-destructive behaviors (BSL-Supplement: IRR, 0.46; 95% CI, 0.28-0.77), and assessor-rated global functioning (CGAS: *d* = 0.43; 95% CI, 0.10-0.79) at 1-month posttreatment, as well as 3-month posttreatment (DERS: *d* = 0.41, 95% CI, 0.09-0.73; BSL-Supplement: IRR, 0.54; 95% CI, 0.33-0.87; CGAS: *d* = 0.48; 95% CI, 0.13-0.87). There were no statistically significant differences between the conditions in psychiatric symptoms (DASS-21) at 1-month or 3-month posttreatment (Table 2). Within-group effect sizes for all outcomes are reported in the eResults in Supplement 1.

Masked assessors were not more accurate than chance at guessing participants’ condition at 1-month posttreatment, although they were slightly more accurate than chance at 3-month posttreatment. The number of assessor-rated NSSI episodes at 1-month posttreatment (β [SE] = 0.29 [0.33], *P* = .38) and 3-month post-treatment (β [SE] = 0.06 [0.31], *P* = .85) did not differ significantly as a function of correctly vs incorrectly guessed treatment allocation. Eleven families revealed the treatment allocation by accident at 1-month posttreatment. Results on primary outcome remained virtually unchanged when the model was rerun without these ratings (eResults in Supplement 1).

The mediation analysis indicated that, relative to TAU only, IERITA plus TAU led to greater decreases in emotion dysregulation, which, in turn, were associated with decreases in NSSI. The estimated indirect (mediated) effect (*ab* = −0.028) was statistically significant (95% bootstrap CI, −0.053 to −0.010) (eResults, eFigures 7 and 8 in Supplement 1). Sensitivity analysis revealed that results were fairly robust to unmeasured pretreatment mediator-outcome confounding (eResults, eFigure 9 in Supplement 1).

Five participants (6%) reported suicide attempts in the IERITA plus TAU condition during the study period, compared with 9 participants (11%) in the TAU only condition. Further information on adverse events is presented in the eResults in Supplement 1.

## Discussion

Findings of this randomized clinical trial support the efficacy of this IERITA delivered adjunctive to TAU (vs TAU only). IERITA resulted in greater improvements in NSSI frequency (both self-reported and assessor-rated) and emotion dysregulation from pretreatment to 1-month posttreatment than TAU only. Results also revealed superior effects of IERITA plus TAU (vs TAU only) on global functioning and other self-destructive behaviors, with between-group effect sizes in the moderate range. Notably, treatment effects were maintained at 3-month posttreatment, suggesting durability of effects. Finally, week-to-week improvements in emotion dysregulation mediated week-to-week reductions in NSSI frequency in IERITA, providing further support for the theoretical framework underlying this treatment.^15^

The conditions did not differ significantly in psychiatric symptoms at 1 or 3 months following treatment, suggesting that IERITA results in improvements in emotion dysregulation and global functioning despite comparable levels of depression and anxiety (relative to TAU). These findings are consistent with the emphasis in IERITA on the acceptance of internal experiences and control of behaviors in the context of emotional distress (vs the control or downregulation of emotions themselves), and suggest that targeting emotion regulation may not be sufficient for the treatment of anxiety and depression among adolescents.

### Strengths and Limitations

This study had several strengths. These include the use of TAU as the control condition, the use of weekly measures of NSSI, inclusion of both self-reported and clinician-administered assessments, integrity of the masked assessments, and the sexual orientation diversity of the sample.

The study also has limitations. IERITA was delivered adjunctive to TAU and compared with TAU only. Although this type of augmentation design holds value in the early stages of treatment development, the lack of equipoise between conditions precludes any conclusions regarding the specific effects of IERITA or mechanistic claims of the intervention. Also, not receiving IERITA could constitute a nocebo condition. Furthermore, although our mediation analysis included a sensitivity analysis to assess the impact of unobserved pretreatment confounders, potential posttreatment confounding (including reverse causality) was not addressed. Future studies should include an active internet-delivered control condition matched on level of activity and attention from therapist and include additional mediators (eg, self-compassion, self-acceptance, other measures of emotion regulation, therapeutic alliance) and analysis (eg, cross-lagged models) to test whether the mediator temporally precedes the outcome. Additionally, data cannot speak to whether participants’ ongoing psychopharmacological treatment was appropriately targeted and dosed. However, given that the onset, type, and frequency of counselling and psychopharmacological treatment were equivalent across conditions, it is unlikely that the observed treatment effects were the result of differences in these additional treatment contacts alone. Furthermore, although 39% of participants were self-referred and may have been particularly motivated to engage in an internet-delivered intervention, most participants were clinician-referred and many of the self-referred patients had previous or ongoing contact with CAMHS, suggesting that the sample is clinically representative. Moreover, the lack of data on interrater reliability of the clinician-administered assessments across clinicians and time is an important limitation and precludes determination of the quality of the clinician-rated outcomes (including measures of NSSI and global functioning), thereby threatening the validity of the findings. Nonetheless, replication of findings across self-reported and clinician-assessed NSSI frequency suggests that findings pertaining to the primary outcome of NSSI were not driven solely by rater differences.

Likewise, the predominantly female sample and focus on adolescents with NSSID limit the generalizability of our findings. Future studies should examine the efficacy of this treatment among self-injuring adolescents without NSSID and with greater gender diversity. Future studies of IERITA should also include longer follow-up periods of at least 1 year. Moreover, at some weeks, the proportion of missing self-reported weekly data was moderate and the wide variety of observed missing data patterns precluded formal sensitivity analysis (treating data as not missing at random). However, intermittent missing observations are common in clinical trials employing weekly measures, and it is often reasonable to assume that these data points are randomly missing.^32^ Moreover, although statistically significant, most treatment effects were in the moderate range, and for some measures, such as self-destructive behaviors, the difference in medians between the 2 conditions was only 1 episode. Future trials could include preregistered thresholds for evaluation of clinical significance.

## Conclusions

Results of this trial suggest that a 12-week, therapist-guided, IERITA delivered adjunctive to TAU is efficacious in the treatment of NSSI and related factors compared with TAU only. Findings that week-to-week improvements in emotion dysregulation were associated with week-to-week reductions in NSSI frequency during IERITA provide preliminary support for the proposed mechanism of change in IERITA.

## Notes

Supplement 1.
eMethods.

eResults.
**eTable 1.** List of Measures and Assessment Points **eTable 2.** Content of the Adolescent Program in Internet-Delivered Emotion Regulation Individual Therapy for Adolescents **eTable 3.** Content of the Parent Program in Internet-Delivered Emotion Regulation Individual Therapy for Adolescents **eTable 4.** Study and Participant Characteristics of Participants with Complete Data and Missing Data on Clinician-Rated Primary Outcome at Primary End Point in IERITA+TAU Condition **eTable 5.** Study and Participant Characteristics of Participants with Complete Data and Missing Data on Clinician-Rated Primary Outcome at Primary End Point in TAU Only Condition **eTable 6.** Between Condition Differences in Study and Participant Characteristics for Participants With Complete Data and Missing Data on Clinician-Rated Primary Outcome at Primary End Point **eTable 7.** Study and Participant Characteristics of Participants Missing ≤20% vs >20% of Weekly Measures in IERITA+TAU Condition **eTable 8.** Study and Participant Characteristics of Participants Missing ≤20% vs >20% of Weekly Measures in TAU Only Condition **eTable 9.** Between Condition Differences in Study and Participant Characteristics for Participants Missing ≤20% and >20% of Weekly Measures **eTable 10.** Model Comparison by AIC and BIC for Count Outcomes at 1-Month Posttreatment and 3-Month Posttreatment **eTable 11.** Treatment Credibility/Expectancy, Satisfaction, Treatment Completion, and Therapist Time Spent on Treatment in Internet-Delivered Emotion Regulation Individual Therapy for Adolescents **eTable 12.** Descriptive Statistics for Self-Reported Nonsuicidal Self-Injury Frequency Week 0 Through 16 **eTable 13.** List of Psychopharmacological Medications Included in the Medication Classes Reported in Table 1 and Table 2 **eFigure 1.** Screenshot of an Interactive Work Sheet From Internet-Delivered Emotion Regulation Individual Therapy for Adolescents **eFigure 2.** Screenshot of a Worksheet With Psychoeducational Text and Illustrations From Internet-Delivered Emotion Regulation Individual Therapy for Adolescents **eFigure 3.** Screenshot of a Worksheet With Psychoeducational Text and Illustrations From Internet-Delivered Emotion Regulation Individual Therapy for Adolescents **eFigure 4.** Screen Shot of Mobile App From Internet-Delivered Emotion Regulation Individual Therapy for Adolescents **eFigure 5.** Missing Observation Occurrence Pattern **eFigure 6.** Histogram Over Nonsuicidal Self-Injury Across Weeks **eFigure 7.** Illustration of the Key Elements of the Parallel Process Growth Model for Mediation Employed in the Current Study **eFigure 8.** Scatterplot of Subject-Specific Latent Growth Rates of the Mediator and Outcome During Treatment and Bootstrap Distribution of the Indirect Effect **eFigure 9.** Sensitivity Analysis of Estimated Mediated Effect

Supplement 2.
Trial Protocol

Supplement 3.
Data Sharing Statement
