# Supplementary material for: Effect of Internet-Delivered Emotion Regulation Individual Therapy for Adolescents With Nonsuicidal Self-Injury Disorder: A Randomized Clinical Trial
Source: JAMA Netw Open. 2023 Jul 13;6(7):e2322069. doi: 10.1001/jamanetworkopen.2023.22069 (PMC10346121; doi:10.1001/jamanetworkopen.2023.22069)
Supplement: Supplement 1. — eMethods. eResults. eTable 1. List of Measures and Assessment Points eTable 2. Content of the Adolescent Program in Internet-Delivered Emotion Regulation Individual Therapy for Adolescents eTable 3. Content of the Parent Program in Internet-Delivered Emotion Regulation Individual Therapy for Adolescents eTable 4. Study and Participant Characteristics of Participants with Complete Data and Missing Data on Clinician-Rated Primary Outcome at Primary End Point in IERITA+TAU Condition eTable 5. Study and Participant Characteristics of Participants with Complete Data and Missing Data on Clinician-Rated Primary Outcome at Primary End Point in TAU Only Condition eTable 6. Between Condition Differences in Study and Participant Characteristics for Participants With Complete Data and Missing Data on Clinician-Rated Primary Outcome at Primary End Point eTable 7. Study and Participant Characteristics of Participants Missing ≤20% vs >20% of Weekly Measures in IERITA+TAU Condition eTable 8. Study and Participant Characteristics of Participants Missing ≤20% vs >20% of Weekly Measures in TAU Only Condition eTable 9. Between Condition Differences in Study and Participant Characteristics for Participants Missing ≤20% and >20% of Weekly Measures eTable 10. Model Comparison by AIC and BIC for Count Outcomes at 1-Month Posttreatment and 3-Month Posttreatment eTable 11. Treatment Credibility/Expectancy, Satisfaction, Treatment Completion, and Therapist Time Spent on Treatment in Internet-Delivered Emotion Regulation Individual Therapy for Adolescents eTable 12. Descriptive Statistics for Self-Reported Nonsuicidal Self-Injury Frequency Week 0 Through 16 eTable 13. List of Psychopharmacological Medications Included in the Medication Classes Reported in Table 1 and Table 2 eFigure 1. Screenshot of an Interactive Work Sheet From Internet-Delivered Emotion Regulation Individual Therapy for Adolescents eFigure 2. Screenshot of a Worksheet With Psychoeducational Text and Illustrations From Inter [file jamanetwopen-e2322069-s001.pdf]

SUPPLEMENTAL ONLINE CONTENT

Bjureberg J, Ojala O, Hesser H, et al. Effect of internet-delivered emotion regulation individual therapy for adolescents with nonsuicidal self-injury disorder: a randomized clinical trial. *JAMA Netw Open.* 2023;6(7):e2322069. doi:10.1001/jamanetworkopen.2023.22069

eMethods.

eResults.

eTable 1. List of Measures and Assessment Points

eTable 2. Content of the Adolescent Program in Internet-Delivered Emotion Regulation Individual Therapy for Adolescents

eTable 3. Content of the Parent Program in Internet-Delivered Emotion Regulation Individual Therapy for Adolescents

eTable 4. Study and Participant Characteristics of Participants with Complete Data and Missing Data on Clinician-Rated Primary Outcome at Primary End Point in IERITA+TAU Condition

eTable 5. Study and Participant Characteristics of Participants with Complete Data and Missing Data on Clinician-Rated Primary Outcome at Primary End Point in TAU-Only Condition

eTable 6. Between Condition Differences in Study and Participant Characteristics for Participants With Complete Data and Missing Data on Clinician-Rated Primary Outcome at Primary End Point

eTable 7. Study and Participant Characteristics of Participants Missing ≤20% vs >20% of Weekly Measures in IERITA+TAU Condition

eTable 8. Study and Participant Characteristics of Participants Missing ≤20% vs >20% of Weekly Measures in TAU Only Condition

eTable 9. Between Condition Differences in Study and Participant Characteristics for Participants Missing ≤20% and >20% of Weekly Measures

eTable 10. Model Comparison by AIC and BIC for Count Outcomes at 1-Month Posttreatment and 3-Month Posttreatment

eTable 11. Treatment Credibility/Expectancy, Satisfaction, Treatment Completion, and Therapist Time Spent on Treatment in Internet-Delivered Emotion Regulation Individual Therapy for Adolescents

eTable 12. Descriptive Statistics for Self-Reported Nonsuicidal Self-Injury Frequency Week 0 Through 16

eTable 13. List of Psychopharmacological Medications Included in the Medication Classes Reported in Table 1 and Table 2

eFigure 1. Screenshot of an Interactive Work Sheet From Internet-Delivered Emotion

© 2023 Bjureberg J et al. *JAMA Network Open.*

Regulation Individual Therapy for Adolescents

eFigure 2. Screenshot of a Worksheet With Psychoeducational Text and Illustrations From Internet-Delivered Emotion Regulation Individual Therapy for Adolescents

eFigure 3. Screenshot of a Worksheet With Psychoeducational Text and Illustrations From Internet-Delivered Emotion Regulation Individual Therapy for Adolescents

eFigure 4. Screen Shot of Mobile App From Internet-Delivered Emotion Regulation Individual Therapy for Adolescents

eFigure 5. Missing Observation Occurrence Pattern

eFigure 6. Histogram Over Nonsuicidal Self-Injury Across Weeks

eFigure 7. Illustration of the Key Elements of the Parallel Process Growth Model for Mediation Employed in the Current Study

eFigure 8. Scatterplot of Subject-Specific Latent Growth Rates of the Mediator and Outcome During Treatment and Bootstrap Distribution of the Indirect Effect

eFigure 9. Sensitivity Analysis of Estimated Mediated Effect

This supplemental material has been provided by the authors to give readers additional information about their work.

© 2023 Bjureberg J et al. *JAMA Network Open.*

eMethods

Informed Consent

In accordance with Swedish law, younger participants (13-14 years of age) provided verbal consent and their guardians provided written consent, whereas older participants (15-17 years of age) provided written consent. All participating parents also provided written informed consent for their own participation.

Blinding Integrity

Recruitment and assessment staff were blind to randomization sequence. All participants received explicit instructions not to reveal their treatment condition to the blinded assessor. To assess the integrity of the blinded ratings, the blinded assessors were instructed to guess the participants' treatment condition after each rating. In line with recommendations for blinding in psychological treatment trials, the blinded assessors were also instructed to specify the reason for their guess, choosing between 1) the family accidently revealed the condition, 2) the level of NSSI reduction, 3) global functioning, or 4) a pure guess.

Reimbursement

Adolescents in both the IERITA plus TAU and TAU only conditions were entitled to receive reimbursement if they completed certain self-report measures and participated in the clinician-administered interviews. For every time-point after the baseline assessment that participants provided data when they were not enrolled in the IERITA intervention, a gift card was loaded with 50 SEK (approx. 5 €). The IERITA plus TAU condition could receive reimbursement for providing data in the month after treatment (i.e., completing self-report measures for the 4 weeks following treatment, and completing the 1-month post-treatment interview) and at 3-months post-treatment (i.e., completing the 3-month post-treatment interview). Hence, the gift cards could be of a maximum value of 50 € for the IERITA plus TAU condition (this condition was also entitled to gift cards at the 12-month follow-up, which is not presented in this study). The TAU only condition could receive reimbursements for completing measures during the treatment period (i.e., 12 weekly self-reports), post treatment (i.e., 4 weekly self-reports and one clinician-administered interview at 1-month post-treatment), and 3-months post-treatment (i.e., one clinician-administered interview). Hence, the gift cards could be of a maximum value of 110 € for TAU only condition. The universal gift card could be used in common shops, e-commerce, and on tickets to events: https://gogift.io/en/se/sek.

Outcomes

Diagnostic Assessments

A full diagnostic interview was conducted using the MINI-KID International Neuropsychiatric Interview, version 6[2] and Body Dysmorphic Disorder Questionnaire (administered as an interview; BDDQ).[3] The Clinician-Administered Nonsuicidal Self-Injury Disorder Index[4] including items corresponding to the Deliberate Self Harm Inventory (DSHI-Y)[5,6] was used to determine if participants met criteria for NSSID, and the Structured Clinical Interview for DSM-IV Personality Disorders Borderline Personality Disorder (BPD) Module[7] was used to assess diagnostic criteria for BPD.

Self-rated Measures

The primary outcome was NSSI frequency as measured by the youth version of the Deliberate Self Harm Inventory (DSHI-Y).[5,6] The DSHI-Y assesses the presence and frequency of the 6 most common forms of NSSI, including cutting, burning, severe scratching, self-biting, self-punching, and head banging.

Emotion dysregulation was assessed pre-, 1-month post-, and 3-months post-treatment using the 36-item Difficulties in Emotion Regulation Scale (DERS).[8] Scores on this measure range from 36 to 180, with higher scores indicating greater emotion dysregulation. The DERS has demonstrated good reliability and construct and convergent validity in adolescents.[9,10] Internal consistency in this sample was excellent (α = ·90).

Emotion dysregulation was also measured once every week during the treatment period and for 4 weeks post-treatment using the 16-item version of the DERS, the DERS-16.[11] Scores on this measure range from 16 to 80, with higher scores indicating greater emotion dysregulation. The DERS-16 has been found to demonstrate good test-retest reliability among adults,[11] as well as good reliability and validity and measurement and structural invariance across age among adolescents[9]. Internal consistency in this sample was excellent (α = .92).

Past week engagement in a variety of risky, self-destructive behaviors (e.g., suicide attempts, risky sexual behavior, binge eating, substance misuse) was measured using the 11-item behavior supplement to the Borderline Symptom List (BSL).[12] Scores on this measure range from 0 to 55. At the end of BSL during weeks 1 to 16, we also added a question regarding suicidal ideation during the past week, where participants

© 2023 Bjureberg J et al. *JAMA Network Open.*

could choose any of the following answers: "*I am not thinking about killing myself*", "*I am thinking about killing myself, but would never do it*", and "*I want to kill myself*".

The DASS-21[13] is a 21-item self-report measure of depression, anxiety, and stress symptoms experienced in the past week. Psychometric studies suggest that it is best represented by a total scale score as an overall marker of psychiatric symptoms.[14] Items are answered on a 4-point Likert-type scale, with higher scores indicating more severe symptoms. The DASS-21 has demonstrated good validity and reliability[14], and its total scale score had good internal consistency in our sample (α =.89).

The following scales were administered to participants in the IERITA plus TAU condition: The Credibility/Expectancy Questionnaire (CEQ)[15] was administered before module 2 to measure treatment credibility and expectancy of IERITA with six items. Evidence for the reliability and predictive validity of this measure has been provided.[15] Following common practice,[16] an overall credibility rating was calculated by taking the average of the first three items of the CEQ (range: 3-27; α in current sample = .80). Expectancy was measured by the item "*By the end of the therapy period, how much improvement in your anxiety do you think will occur?*" (range 0-100 in 10-point increments). Higher scores indicate greater credibility and expectancy. The Client Satisfaction Questionnaire[17] (range: 8-32) was administered at 1-month post-treatment to determine levels of treatment satisfaction in IERITA (α in current sample = .94). Higher scores indicate greater satisfaction. Self-reported adverse events related to participation in IERITA plus TAU were assessed at 1-month post-treatment. Participants were asked to report and describe the adverse events, as well as to rate the discomfort of potential adverse events (both when the event occurred and currently, to assess residual discomfort) on a scale of 0 (*did not affect me at all*) to 3 (*affected me very negatively*).[18]

Blinded Assessor-rated Measures

Global functioning was assessed using the Children's Global Assessment Scale (CGAS).[19] Scores on this measure range from 0-100, with higher scores indicating better functioning. Evidence for moderate to excellent inter-rater reliability, good stability over time, and good concurrent and discriminant validity has been provided.[19,20] The interrater reliability was excellent for the CGAS on two practice cases (intraclass correlation coefficient = .93; 95% CI, .72-1.0).

Participant and Therapist Activity

Participant activity, therapist time per youth and parent, and number of online messages were logged in the treatment platform.

Interventions

Prior to randomization, all participants (both conditions) were also provided with individualized treatment recommendations from the research team (typically either recommendations to continue existing care and/or referrals to specialized care). The research team also coordinated with treatment providers on an as-needed basis as a safety measure. Importantly, because weekly self-rated assessments and follow-ups were included in both conditions, and treatment recommendations were provided before randomization, IERITA also included all elements of TAU and therefore constituted an add-on intervention to TAU.

If participants did not have access to the internet in their homes, the clinicians were instructed to problem-solve so the families could access the treatment from schools and libraries.

Mobile App

The participants were instructed to use the app daily to register self-destructive behaviors, impulses to act destructively, and potential protective factors. The app also included several programs that could be used to practice skills taught in the online modules, reminders of homework assignments, module summaries, and an individualized crisis plan. See eFigure 4. If participants did not have access to a smartphone, they could access the app via the desktop version of the treatment.

Safety Routines

A robust participant safety management protocol was established before study start. All study therapists and relevant study staff received training in suicide risk assessment and management before participant recruitment commenced. Participants, regardless of treatment condition, were carefully monitored by a study coordinator and an assigned therapist. Participants were instructed to complete online weekly assessments of NSSI, other self-destructive behaviors and suicidal ideation, and were automatically flagged in the assessment platform if sudden deterioration was detected. Participants who did not complete the assessment were contacted via telephone. To increase participant safety and enhance linkage to outpatient treatment, participants (adolescents and/or parents) were immediately contacted if any assessment indicated deterioration in health. Any event

© 2023 Bjureberg J et al. *JAMA Network Open.*

relevant to the participants' health care was noted in the electronic health record. Two senior child and adolescent psychiatrists were available for consultation based on clinical need. An individualized crisis plan was created at the face-to-face assessment and included information about who to contact in acute situations and an agreement to follow the crisis plan. The crisis plan contained necessary contact information for the study therapist and acute health care services. Further, weekly treatment conferences including a senior child and adolescent psychiatrist, a senior psychologist, and a study coordinator (psychologist) with extensive clinical experience in the treatment of self-injury were held throughout the course of the trial to monitor the safety, including adverse events, and clinical worsening of the participants. This group acted as an internal safety monitoring board that consulted with an external senior consultant (i.e., a specialist in child and adolescent psychiatry and certified DBT therapist) on an as-needed basis.

Statistical Analysis

Power

Statistical power was estimated using a 0.05-level Wald test for the interaction between a binary treatment variable and linear time in treatment (12 weeks) in a zero-inflated negative binomial regression model using 200 bootstrap samples. The data for the treated group were obtained by sampling with replacement from the available data from our previous open trial of IERITA.[21]

Outliers and Missing Data

The data were visually inspected using histograms (figures not shown) to check for distribution assumptions and outliers. Intermittent missing observations are common in clinical trials employing weekly measures, and it is often reasonable to assume that these data points are randomly missing.[22] Any missing observations (see Figure 1 for a breakdown of number of completed assessments) were assumed to be missing at random after visual inspection of the pattern of the missing values (eFigure 5), distribution of primary outcome across weeks (eFigure 6), and analyses of missingness in relation to study and participant characteristics. No distinct missing data patterns were identified and no differences in missing data patterns between the two treatment groups were found (eFigure 5). Furthermore, the distribution of the primary outcome across weeks did not seem to be affected by differences in missing data occurrence over time (eFigure 6). Analyses of study and participant characteristics for participants missing ≤ 20% of weekly measures versus missing > 20% within IERITA+TAU (eTable 7) and TAU only (eTable 8), as well as analyses examining between-condition differences for participants missing ≤ 20% and > 20% of weekly measures (eTable9), suggested that missingness was not strongly related to baseline characteristics within or between conditions. Corresponding analyses conducted for participants with complete data versus missing data on the primary outcome of clinician-rated NSSI at 1-month post-treatment (eTable 4, eTable 5, eTable 6) supported the same conclusion.

Self-reported NSSI Frequency Week 0 Through Week 16

The primary outcome analysis of self-reported NSSI frequency included treatment condition and weekly reports of NSSI frequency (DSHI-Y) measured once immediately before the start of treatment, once every week during treatment, and once every week for four weeks after treatment termination (1-month post-treatment). The 3 preceding pre-treatment measurement points were considered "burn-in" measures and not included in the analysis.

To examine if certain TAU or client characteristics influence the efficacy of IERITA, we ran exploratory moderation analyses examining specific aspects of TAU and client sexual orientation as moderators of treatment effects. Examined moderators included frequency of TAU (5 ordinal levels), medication (yes/no), CBT (yes/no), supportive therapy (yes/no), and unknown TAU (yes/no), and sexual orientation (heterosexual or sexual minority). Analyses used separate zero-inflated negative binomial generalized linear mixed effects regression models to estimate the rate of change in NSSI counts as a function of treatment condition and each moderator variable (in separate analyses). The models included fixed effects of time, treatment condition, moderator, and their interactions, and subject-specific random effects for intercept and linear time for the count part of the model. Frequency of TAU was centered before being entered into the model as a predictor. We investigated whether levels of the moderator influenced change over time across treatment conditions (i.e., two-way interaction between moderator and time) and differential rate of change in NSSI count as a function of treatment condition (i.e., three-way interaction between moderator, treatment condition, and time).

Effect Sizes and Data Visualization

For count outcomes, exponentiated marginal coefficients[23] (i.e., population-averaged incidence rate ratios [IRRs]) were presented, and corresponding predictions along with their 95% confidence intervals (CI) were plotted against time to visualize the development per week and treatment group. The robust sandwich estimator was used for the

© 2023 Bjureberg J et al. *JAMA Network Open.*

estimation of the standard errors. The effects for all other outcomes were evaluated with Cohen's *d* for mixed effects models with bootstrap (99 simulations) confidence intervals.[24] The development over time was visualized as estimated marginal means and their 95% CI.

Mediation Analysis

Parallel process latent growth curve modelling (PPGM; aka. dual-trajectory growth curve model) is a recommended method for evaluating mediation when the mediator and outcome variables are collected at repeated time points because the approach allows for estimation of individual differences in change. Further, variation in individual change in the mediator can be related to change in the outcome in a single combined growth model that also takes into account missing data and dependence due to repeated measurements.[25,26]

We followed recommendations for the evaluation of PPGM.[27] Univariate latent growth models for the outcome and mediator for the weekly measured variables were specified in a similar way as those estimated with generalized linear mixed models (i.e., zero-inflated negative binomial and continuous latent growth models, respectively) and provided similar estimates of key parameters. The two univariate latent growth curve models for the outcome and mediator were combined into a PPGM. Study site and initial levels on outcome and mediator variables were statistically covaried in the model. Mediation was evaluated at the latent level (i.e., continuous random effects) and by using linear regression to relate the observed binary treatment variable (IERITA plus TAU = 1, TAU only = 0), the latent growth rate factor of the mediator, and the latent growth rate factor of the outcome (log rate count trajectory part of the model; individual variation and change in NSSI counts). See eFigure 7 for an illustration of the key elements of the PPGM employed in the current study. Mediation was supported when IERITA plus TAU, relative to TAU only, changed the latent growth factor of the mediator (a-path) and this, in turn, influenced the latent growth factor of the outcome (b-path). The point estimate of the mediated (indirect) effect (i.e., the ab-product which was the product of the a- and b-path) was significance tested with a bias-corrected bootstrapped 95% CI, constructed with the empirical distribution of 3000 bootstrap samples drawn with replacement. If the bias-corrected confidence interval did not include zero, the mediated effect was considered to be statistically significant.

Given that the magnitude of the indirect effect varies as a function of the values of the predictors in models with nonlinear associations (e.g., loglinear regression),[28] we also computed conditional indirect effects at different values of the treatment variable (0, 1) as detailed in[29,30]. These analyses revealed that the estimate of the indirect effect was slightly lower in magnitude for participants in IERITA plus TAU relative to those in TAU only, but the findings did not alter the overall conclusions about mediation and are therefore not further presented.

A sensitivity analysis was performed to examine the impact of unmeasured pre-treatment mediator-outcome confounding (aka. sequential ignorability assumption)[31]. In the sensitivity analysis, the correlation between the error terms in the mediator and the outcome growth models was fixed at different values (-.7 to .7) to determine whether this altered the point estimate and significance of the indirect effect (see Figure S9).

Statistical Software

The descriptive and outcome analyses were conducted using the statistical software R version 4.1.0.[32] The package GLMMadaptive[33] was used for fitting generalized linear mixed effects regression models and obtaining and plotting marginal coefficients. The package lme4[34] was used for fitting generalized linear mixed effects regression models, and the package ggeffects[35] was used for estimating and plotting marginal means.

In secondary analysis, we compared number of participants in each treatment condition with no NSSI episodes at 1-month post-treatment and at 3-months post-treatment using $\chi 2$.
The mediation analysis was evaluated with Mplus vs. 8.1,[36] and PPGM was estimated with maximum likelihood and the EM algorithm (in combination with numerical integration) using all available observations according to intention-to-treat and the assumption of missing at random.

eResults

Participants enrolled in treatment during the COVID-19 pandemic (i.e., included after December 1, 2019; n=16;19%) completed on average 9.0 (SD=2.4) modules and parents completed on average 5.4 (SD=1.0) modules.

Blinding Integrity

Eleven families revealed their treatment condition (7 IERITA plus TAU and 4 TAU only) by accident at 1-month post-treatment and 4 families revealed their treatment condition at 3-months post-treatment (1 IERITA plus TAU and 3 TAU only). Post-hoc analyses revealed that the estimates, p-values, and IRR remained virtually

© 2023 Bjureberg J et al. *JAMA Network Open.*

unchanged for 1-month post-treatment (β=-1.18; SE=0.30, < .001, IRR=0.31, 095 CI [0.33, -0.77]) and 3-months post-treatment (β=-0.63, SE = 0.30, *P*=0.032, 95% CI [0.30, 0.94]) when the model was rerun without these ratings. Given these results, we present the models with the full sample as the main results. Excluding the eleven families at 1-month post-treatment and 4 families at 3-months post-treatment, the blinded assessors guessed the correct treatment condition in 55% of the cases at 1-month post-treatment ($\chi2(1)=1.26$, p=.262) and 60% of the cases at 3-months post-treatment ($\chi2(1)=6.12$, *P*=.013). These findings indicate that the blinded assessors were not better at guessing treatment condition than expected by change (50%) at 1-month post-treatment, but were statistically significantly better at guessing treatment condition than expected by chance at 3-months post-treatment. The majority (58%) were guesses based on the participant's reduction of NSSI and 35% were pure guesses at 1-month post-treatment. The corresponding numbers for 3-months post-treatment were 51% and 44%.

Primary Outcomes

The within-group reductions in blinded assessor-rated NSSI frequency from pre-treatment to 3-months post-treatment was 84% (IRR 0.16, 95% CI, 0.08, 0.31) in IERITA plus TAU and 70% (IRR 0.30, 95% CI, 0.14, 0.62) in TAU only.

Exploratory post-hoc analyses indicated that 43 of 77 (55.8%) participants in IERITA reported an absence of NSSI (0 episodes) at 1-month post-treatment, compared to 26 of 77 (33.8%) in TAU-only (a statistically significant difference, $\chi2=7.59$, *P*=.006). The corresponding analysis was non-significant at 3-months post-treatment.

None of the examined TAU characteristics or sexual orientation emerged as unspecific predictors or moderators (i.e., none of the two-way or three-way interactions approached statistical significance).

Secondary Outcomes

Participants in IERITA plus TAU evidenced statistically significant reductions in self-reported self-destructive behaviors (BSL; IRR=0.23, 95% CI, 0.15, 0.36), self-reported emotion dysregulation (DERS; *d*=0.85, 95% CI, 0.58, 1.12), self-reported psychiatric symptoms (DASS; *d*=0.29, 95% CI, 0.08, 0.51), and blinded assessor-rated global functioning (CGAS, *d*=0.79, 95% CI, 0.54, 1.05) from pre-treatment to 1-month post-treatment. Furthermore, the same patterns were found for the period from pre-treatment to 3-months post-treatment (CGAS: *d*=1.14, 95% CI, 0.86, 1.43; DASS: *d*=0.48, 95% CI, 0.27, 0.68; DERS: *d*=1.03, 95% CI, 0.80, 1.25; BSL: IRR=0.30, 95% CI, 0.22, 0.42). Participants in TAU only experienced statistically significant reductions in self-reported emotion dysregulation (DERS; *d*=0.33, 95% CI, 0.10, 0.58), self-reported self-destructive behaviors (BSL; IRR=0.50, 95% CI, 0.38, 0.66), and blinded assessor-rated global functioning (CGAS; *d*=0.36, 95% CI, 0.12, 0.58), but no significant reduction in self-reported psychiatric symptoms (DASS; *d*=0.13, 95% CI, -0.08, 0.36) from pre-treatment to 1-month post-treatment. Furthermore, the same patterns were found for the period from pre-treatment to 3-months post-treatment (CGAS: *d*=0.66, 95% CI, 0.41, 0.88; DERS: *d*=0.62, 95% CI, 0.39, 0.84; BSL: IRR=0.57, 95% CI, 0.38, 0.85), although there was also a significant reduction in self-reported psychiatric symptoms during this time (DASS: *d*=0.36, 95% CI, 0.13, 0.58).

Mediation Analysis

IERITA plus TAU (vs. TAU only) was negatively associated with the latent growth rate factor of the mediator, emotion dysregulation (a[SE]=-0.418 [0.132], p=.002). Further, individual change in emotion dysregulation was positively associated with individual change in NSSI frequency (b[SE]=0.067 [0.014], *P*<.001). The estimated indirect (mediated) effect (ab =-0.028) was statistically significant as tested with a 95% bootstrap CI (-0.053, -0.010). Findings from the sensitivity analysis are depicted in eFigure 9. As can be seen, the results were fairly robust to possible violations of mediator-outcome confounding, given that it would require a large size residual correlation (Rho ≈.53) to reduce the point estimate of the indirect effect to zero (see eFigure 9). After taking uncertainty of sampling variability into account, the 95% CI covered zero with a correlation that exceeded .36. When compared to other studies that have used the same sensitivity analysis (e.g.,[37]), our findings indicate a moderate degree of robustness to unmeasured pre-treatment confounders (see [38, p. 151]).

Adverse Events

Out of the 80 participants who completed the self-report form measuring adverse events, 16 participants (20.0%) enrolled in IERITA plus TAU reported having experienced negative effects during the treatment period, of which 4 (5.0%) were determined to be related to the treatment at post-treatment. Two participants reported experiencing increased sadness (immediate effect 0 and 2; residual effects 0), one participant reported experiencing increased stress (immediate effect=1 and 2, residual effect=0), and one participant reported an increase in self-destructive behaviors (immediate effect=2, residual effect=0) because of the study. Five participants (6%) reported suicide attempts in the IERITA plus TAU condition, compared to 8 participants

© 2023 Bjureberg J et al. *JAMA Network Open.*

(10%) in the TAU only condition during the treatment period. One participant reported a suicide attempt in the TAU only condition at 3-months post-treatment. One participant in the IERITA plus TAU condition was reported dead by accident at 3-months post-treatment. It is standard procedure in Sweden to investigate any potential suicide attempts. Such investigations typically involve all treatment contacts with which the deceased has been engaged. The research group was not contacted in such investigation.

© 2023 Bjureberg J et al. *JAMA Network Open*.

eReferences

1. Mataix-Cols D, Andersson E. Ten Practical Recommendations for Improving Blinding Integrity and Reporting in Psychotherapy Trials. *JAMA psychiatry.* 2021; **78**(9): 943–944.
2. Sheehan DV, Lecrubier Y, Sheehan KH, et al. The Mini- International Neuropsychiatric Interview (M.I.N.I.): the development and validation of a structured diagnostic psychiatric interview for DSM- IV and ICD- 10. *The Journal of clinical psychiatry.* 1998; **59**: 20–22.
3. Phillips KA. *Understanding Body Dysmorphic Disorder: An Essential Guide.* Oxford University Press; 2009.
4. Gratz KL, Dixon-Gordon KL, Chapman AL, Tull MT. Diagnosis and Characterization of DSM-5 Nonsuicidal Self-Injury Disorder Using the Clinician-Administered Nonsuicidal Self-Injury Disorder Index. *Assessment.* 2015; **22**(5): 527–539.
5. Gratz KL, Latzman RD, Young J, et al. Deliberate Self-Harm Among Underserved Adolescents: The Moderating Roles of Gender, Race, and School-Level and Association With Borderline Personality Features. *Personality disorders: Theory, Research, and Treatment.* 2012; **3**(1): 39–54.
6. Gratz KL. Measurement of Deliberate Self-Harm: Preliminary Data on the Deliberate Self-Harm Inventory. *Journal of psychopathology and behavioural assessment.* 2001; **23**(4): 253–263.
7. First MB, Gibbon M, Spitzer RL, Williams JBW, Benjamin LS. *Structured clinical interview for DSM-IV axis II personality disorders, (SCID-II).* Washington, DC: American Psychiatric Association; 1997.
8. Gratz KL, Roemer L. Multidimensional Assessment of Emotion Regulation and Dysregulation: Development, Factor Structure, and Initial Validation of the Difficulties in Emotion Regulation Scale. *Journal of Psychopathology and Behavioural Assessment.* 2004; **26**(1): 41–54.
9. Monell E, Birgegård A, Nordgren L, Hesser H, Bjureberg J. Factor structure and clinical correlates of the original and 16-item version of the Difficulties In Emotion Regulation Scale in adolescent girls with eating disorders. *Journal of clinical psychology.* 2022; **78** (6): 1201–1219
10. Neumann A, van Lier PAC, Gratz KL, Koot HM. Multidimensional assessment of emotion regulation difficulties in adolescents using the difficulties in Emotion Regulation Scale. *Assessment (Odessa, Fla).* 2010; **17**(1): 138–149.
11. Bjureberg J, Ljótsson B, Tull MT, et al. Development and Validation of a Brief Version of the Difficulties in Emotion Regulation Scale: The DERS-16. *Journal of psychopathology and behavioural assessment.* 2016; **38**(2): 284–296.
12. Bohus M, Limberger MF, Frank U, Sender I, Gratwohl T, Stieglitz R-D. Development of the borderline symptom list. *Psychotherapie, Psychosomatik, Medizinische Psychologie.* 2001; **51**(5): 201–211.
13. Lovibond SH, Lovibond PF. The structure of negative emotional states: comparison of the Depression Anxiety Stress Scales (DASS) with the Beck Depression and Anxiety Inventories. *Behaviour Research and Therapy.* 1995; **33**(3): 335–343.
14. Osman A, Wong JL, Bagge CL, Freedenthal S, Gutierrez PM, Lozano G. The Depression Anxiety Stress Scales-21 (DASS-21): Further Examination of Dimensions, Scale Reliability, and Correlates. *Journal of clinical psychology.* 2012; **68**(12): 1322–1338.
15. Devilly GJ, Borkovec TD. Psychometric properties of the credibility/expectancy questionnaire. *Journal of behaviour therapy and experimental psychiatry.* 2000; **31**(2): 73–86.
16. Thompson-Hollands J, Bentley KH, Gallagher MW, Boswell JF, Barlow DH. Credibility and Outcome Expectancy in the Unified Protocol: Relationship to Outcomes. *Journal of experimental psychopathology.* 2014; **5**(1): 72–82.
17. Attkisson CC, Zwick R. The client satisfaction questionnaire: Psychometric properties and correlations with service utilization and psychotherapy outcome. *Evaluation and Program Planning.* 1982; **5**(3): 233–237.
18. Ljótsson B, Hesser H, Andersson E, et al. Provoking symptoms to relieve symptoms: A randomised controlled dismantling study of exposure therapy in irritable bowel syndrome. *Behaviour research and therapy.* 2014; **55**: 27–39.
19. Shaffer D, Gould MS, Brasic J, et al. A Children's Global Assessment Scale (CGAS). *Archives of general psychiatry.* 1983; **40**(11): 1228–1231.
20. Lundh A, Kowalski J, Sundberg CJ, Gumpert C, Landén M. Children's Global Assessment Scale (CGAS) in a naturalistic clinical setting: Inter-rater reliability and comparison with expert ratings. *Psychiatry research.* 2010; **177**(1): 206–210.
21. Bjureberg J, Sahlin H, Hedman-Lagerlöf E, et al. Extending research on Emotion Regulation Individual Therapy for Adolescents (ERITA) with nonsuicidal self-injury disorder: open pilot trial and mediation analysis of a novel online version. *BMC Psychiatry.* 2018; **18**(1): 326.
22. Hedeker D, Gibbons R. D. Application of random-effects pattern-mixture models for missing data in longitudinal studies. *Psychological Methods*. 1997; 2: 64–78.
23. Hedeker D, du Toit SHC, Demirtas H, Gibbons RD. A note on marginalization of regression parameters from mixed models of binary outcomes: A Note on Marginalization. *Biometrics.* 2018; **74**(1): 354–361.

© 2023 Bjureberg J et al. *JAMA Network Open.*

24. Feingold A. New approaches for estimation of effect sizes and their confidence intervals for treatment effects from randomised controlled trials. *The quantitative methods for psychology.* 2019; **15**(2): 96–111.
25. Cheong J. Accuracy of Estimates and Statistical Power for Testing Meditation in Latent Growth Curve Modeling. *Structural equation modeling.* 2011; **18**(2): 195–211.
26. Hesser H. Modeling individual differences in randomised experiments using growth models: Recommendations for design, statistical analysis and reporting of results of internet interventions. *Internet interventions : the application of information technology in mental and behavioural health.* 2015; **2**(2): 110-120.
27. Cheong J, MacKinnon DP, Khoo ST. Investigation of Mediational Processes Using Parallel Process Latent Growth Curve Modeling. *Structural equation modeling.* 2003; **10**(2): 238-262.
28. Muthén B, Asparouhov T. Causal Effects in Mediation Modeling: An Introduction With Applications to Latent Variables. *Structural equation modeling.* 2015; **22**(1): 12-23.
29. Geldhof GJ, Anthony KP, Selig JP, Mendez-Luck CA. Accommodating binary and count variables in mediation: A case for conditional indirect effects. *International journal of behavioural development.* 2018; **42**(2): 300–308.
30. O'Rourke HP, Vazquez E. Mediation analysis with zero-inflated substance use outcomes: Challenges and recommendations. *Addictive behaviours.* 2019; **94**: 16–25.
31. Imai K, Keele L, Tingley D. A General Approach to Causal Mediation Analysis. *Psychological methods.* 2010; **15**(4): 309–334.
32. R Core Team. R: A language and environment for statistical computing. Vienna, Austria: R Foundation for Statistical Computing; 2021.
33. Rizopoulos D. GLMMadaptive: Generalized Linear Mixed Models using Adaptive Gaussian Quadrature. R package version 0.8-2. https://CRAN.R-project.org/package=GLMMadaptive. Published 2021.
34. Bates D, Mächler M, Bolker B, Walker S. Fitting Linear Mixed-Effects Models Using lme4. *Journal of Statistical Software.* 2015; **67**(1): 1–48.
35. Lüdecke D. ggeffects: Tidy data frames of marginal effects from regression models. *Journal of Open Source Software.* 2018; **3**(26): 772.
36. Muthén LK, Muthén BO. *Mplus User's Guide*. Eigth ed. Los Angeles, CA: Muthén & Muthén; 1998-2017
37. Imai Kosuke, Keele Luke, Tingley Dustin, Yamamoto Teppei. Unpacking the Black Box of Causality: Learning about Causal Mechanisms from Experimental and Observational Studies. *American Political Science Review*. 2011;105(4):765-789.
38. Imai K, Yamamoto T. Identification and Sensitivity Analysis for Multiple Causal Mechanisms: Revisiting Evidence from Framing Experiments. *Political Analysis*. 2013;21(2):141-171.

eTable 1. List of Measures and Assessment Points

Measure	Pre-treatment	During treatment (Weeks 1-16)	1-month post-treatment	3-months post-treatment
**Diagnostic Interviews**				
CANDI	X			
MINI KID+BDDQ	X			
SCID-II-BPD	X			
**Blinded assessor-rated**				
NSSI (CANDI DSHI-Y)	X		X	X
Global functioning (CGAS)	X		X	X
**Self-rated**				
NSSI (DSHI-Y)	X	X	X	
Risky behaviors (BSL-Supplement)	X	X	X	X
Emotion dysregulation (DERS)	X		X	X
Emotion dysregulation (DERS-16)		X		
Psychiatric symptoms (DASS-21)	X		X	X
**Self-rated treatment-related measures**				
Credibility/ Expectancy (CEQ)		X[a]		
Treatment satisfaction (CSQ)			X	
Adverse events			X	

Abbreviations: 1MFU, 1-month follow-up; 3MFU, 3-month follow-up; BDDQ, Body Dysmorphic Disorder Questionnaire; BSL, Borderline Symptom List; CANDI, Clinician-Administered Nonsuicidal Self-Injury Disorder Index; CEQ, The Credibility/Expectancy Questionnaire; CGAS, Children's Global Assessment Scale; CSQ, Client Satisfaction Questionnaire; DASS-21, Depression Anxiety Stress Scales, 21 item version; DERS, Difficulties in Emotion Regulation Scale; DERS-16, Difficulties in Emotion Regulation Scale, 16-item version; DSHI-Y, Deliberate Self-Harm Inventory – Youth Version; SCID-II-BPD, Structured Clinical Interview for DSM-IV Personality Disorders Borderline Personality Disorder Module.

[a]Administered before module 2.

© 2023 Bjureberg J et al. *JAMA Network Open.*

eTable 2. Content of the Adolescent Program in Internet-delivered Emotion Regulation Individual Therapy for Adolescents

Module	Theme	Content
**Module 1**	**Psychoeducation**	• Information about how internet-delivered treatment works and advice on how to structure the treatment work<br>• Psychoeducation about NSSI and other self-destructive behaviors<br>• Emotional avoidance and how it is associated with NSSI<br>• Identify short- and long-term consequences of NSSI<br>• Introduction to fictional characters to follow through the treatment<br><br>*Homework:* Register NSSI acts and impulses (or absence of such acts or impulses) and related contextual factors (e.g., preceding emotions and thoughts)
**Module 2**	**Impulse control training**	• Psychoeducation about impulses and impulsive/planned behavior<br>• Learning and practicing skills to identify and observe an impulse without acting<br>• Impulse-control training: (1) distraction from NSSI impulses, (2) choose another behavior fulfilling similar functions as NSSI but without long-term negative consequences, and (3) remind yourself of negative long-term consequences of NSSI/impulsive behavior<br><br>*Homework:* Continue NSSI registration and practice impulse control strategies.
**Module 3**	**Emotional awareness**	• Psychoeducation about emotions: what they are, their components (thoughts, bodily sensations, and action urges), their functions and why we have them, and common emotions and how they can be experienced<br>• Practicing emotional awareness<br><br>*Homework*: Practice emotional awareness.
**Module 4**	**Emotional awareness/clarity**	• Identify one's own negative beliefs about emotions<br>• Psychoeducation about primary and secondary emotions<br>• Practice identifying primary and secondary emotions<br>• Identify and manage secondary emotions; e.g., notice if there are any negative beliefs about emotions that are eliciting secondary emotions, describe these thoughts as thoughts, and return attention back to the primary emotion<br>• Practice alternative ways to approach emotions based on what has been taught in module 3<br><br>*Homework*: Continue practicing emotional awareness (now also distinguishing between primary and secondary emotions) + identify thoughts as thoughts.
**Module 5**	**Emotional acceptance**	• Learn about the negative consequences of emotional avoidance and identity one's own behaviors that function to avoid and control emotions<br>• Learn to be willing to have and experience emotions and still do what is meaningful in life<br>• Practice sitting with discomfort without trying to change or modify the experience<br><br>*Homework*: (1) Practice noticing when you try to avoid/control emotions as well as when you are not trying to change the emotional experience. (2) Plan to do something that is important to you and try to follow through regardless of whether painful emotions occur.

© 2023 Bjureberg J et al. *JAMA Network Open.*

eTable 2. Content of the Adolescent Program in Internet-delivered Emotion Regulation Individual Therapy for Adolescents (continued)

Module	Theme	Content
**Module 6**	**Emotion regulation strategies**	• Psychoeducation on adaptive and maladaptive emotion regulation strategies: emotional approach, distraction, and avoidance<br>• Emphasis on the advantages of emotional approach (i.e., identify, label, and investigate what information the emotion is providing, and identify how to act according to that information)<br>• Clarify how emotional approach and distraction strategies may work together: e.g., in some situations, distraction may be helpful for a short time, if the goal is to return attention to the emotion at a later time-point<br>• Identify different emotional approach and distraction strategies to test from lists of suggestions<br>• Half-time evaluation of how the treatment work is going and investigate additional need for support<br><br>*Homework*: Practice emotional approach and distraction.
**Module 7**	**Emotion regulation strategies**	• Strategies for implementing emotion regulation strategies in situations where one is at risk of destructive behaviors<br>• Rate present destructive behaviors according to the level of suffering and impairment associated with the behavior, identify common contextual factors surrounding a given destructive behavior and alternative emotion regulation strategies that might be helpful instead, given these contextual factors<br>• Advice on how to remember to practice skills and handle worry that might emerge<br><br>*Homework:* Continue practicing emotional approach and distraction in risk situations of destructive behavior.
**Module 8**	**Validation**	• Psychoeducation on validation and invalidation<br>• Note risk situations for self-invalidation and the effects of self-invalidation<br>• Teaching skills in self-validation: (1) describe without judging, (2) change perspective (talk to yourself as a friend), (3) experience the emotion as it is, and (4) be open to validation from yourself or others<br><br>*Homework:* (1) Continue using emotional approach and distraction in risk situations for destructive behaviors. (2) Notice self-invalidation and practice self-validation.
**Module 9**	**Valued direction**	• Identify valued directions within friendship, health, personal development, school, and family domains<br>• Come up with specific actions that correspond to valued directions in these different areas<br>• Psychoeducation on both external and internal obstacles to valued directions<br>• Manage external obstacles through problem-solving and manage internal obstacles through emotional approach and distraction<br><br>*Homework:* (1) Continue using emotional approach and distraction in risk situations for destructive behaviors. (2) Engage in at least two valued actions consistent with your valued directions.

eTable 2. Content of the Adolescent Program in Internet-delivered Emotion Regulation Individual Therapy for Adolescents (continued)

© 2023 Bjureberg J et al. *JAMA Network Open.*

Module	Theme	Content
**Module 10**	**Summary and Practice**	• Summary of the treatment content<br>• Common pitfalls with the strategies and how to manage them<br>• Examples of how to combine all the different strategies<br><br>*Homework:* Repetition of skills according to preference.
**Module 11**	**Relapse prevention**	• Personal evaluation of potential changes and what have been the most helpful parts of treatment<br>• Identify risk situations for setbacks/relapse and suggest strategies to handle them<br>• Inform about setbacks and encourage skills training in those situations<br>• Option to download all the material

© 2023 Bjureberg J et al. *JAMA Network Open*.

eTable 3. Content of the Parent Program in Internet-delivered Emotion Regulation Individual Therapy for Adolescents

Module	Theme	Content
**Module 1**	**Psychoeducation**	• Information about how internet-delivered treatment works and advice on how to structure the treatment work<br>• Psychoeducation about NSSI and emotional reactivity<br><br>*Homework*: Observe emotional reactivity and its consequences in yourself and others.
**Module 2**	**Emotional awareness**	• Psychoeducation about emotions: what they are, their components (thoughts, bodily sensations, and action urges), why we have them, and common emotions and how they can be experienced<br>• The difference between describing and judging emotions<br><br>*Homework*: Practice emotional awareness
**Module 3**	**Validation and invalidation**	• Psychoeducation about validation and invalidation<br>• Learn about contextual factors that can contribute to an invalidating environment (e.g., not noticing the emotions that are expressed, not knowing what to do)<br>• Notice situations where one has been invalidating<br>• Learn that validation can be a way to regulate emotions and have a positive impact on relationships<br>• Practice self-validation<br>• Examples of how one can validate others<br>• Half-time evaluation of how the treatment work is going. Investigate additional need for support<br><br>*Homework:* Practice validation of others; try three different ways to validate.
**Module 4**	**Self-validation and self-invalidation**	• Common pitfalls with validation and how to handle them<br>• Psychoeducation on self-invalidation, the contexts and situations in which it can occur, and what emotions it can lead to<br>• Learn and practice self-validation.<br><br>*Homework:* (1) Continue practicing validation of others. (2) Practice self-validation once a day.
**Module 5**	**Behavioral activation**	• Learn about behavioral activation for yourself<br>• Learn about behavioral activation together with your adolescent<br>• Strategies to ask the adolescent to engage in an activity together<br>• Suggestions of activities to do together to get more positive time together<br><br>*Homework*: (1) Engage in activities that are enjoyable or relaxing (2) Engage in activities together with the adolescent on the adolescent's terms.
**Module 6**	**Summary**	• Summary and follow-up<br>• Plan for continued practice of skills<br>• Evaluation of what has been helpful<br>• Possibility to download all the material

The parents also had access to PDF-files of the adolescent modules.

© 2023 Bjureberg J et al. *JAMA Network Open.*

eTable 4. Study and Participant Characteristics of Participants with Complete Data and Missing Data on Clinician-Rated Primary Outcome at Primary End-Point in IERITA+TAU Condition

	Complete Data	Missing Data	Chi2/T	*P*
**No. (%)**	77 (92)	7 (8)		
**Study Characteristics**				
Site			0.399	.819
Skåne	35 (45)	3 (43)		
Stockholm	25 (32)	3 (43)		
Västra Götaland	17 (22)	1 (14)		
Source of referral			0.235	.628
Clinician	51 (66)	4 (57)		
Self	26 (34)	3 (43)		
**Participant Characteristics**				
Gender			0.694	.707
Female	70 (91)	7 (100)		
Male	5 (6)	0 (0)		
Non-binary	2 (3)	0 (0)		
Age, mean (SD)	15.01 (1.29)	15.42 (1.57)	0.787	.434
Any failed grades (yes)	9 (12)	2 (29)	1.607	.205
**Participant Clinical Characteristics**				
Age NSSI onset, mean (SD)	12.73 (1.17)	12.43 (2.23)	0.594	.554
Years since NSSI onset, mean (SD)	2.28 (1.23)	2.99 (2.41)	1.328	.188
Comorbidity[a]				
Major depressive disorder	44 (57)	5 (71)	0.538	.463
Anxiety disorders				
Social anxiety disorder	23 (30)	1 (14)	0.764	.382
Panic disorder/Agoraphobia	15 (20)	2 (29)	0.329	.567
Specific phobia disorder	12 (16)	2 (29)	0.779	.377
Generalized anxiety disorder	12 (16)	0 (0)	1.273	.259
ADHD[b]	13 (17)	1 (14)	0.031	.860
Autism spectrum disorder	4 (5)	0 (0)	0.382	.537
OCD/BDD	3 (4)	0 (0)	0.283	.595
Eating disorder[c]	6 (8)	0 (0)	0.587	.443
Oppositional defiant disorder	3 (4)	0 (0)	0.283	.595
Mean (SD) number of co-occurring disorders	1.88 (1.70)	1.57 (1.13)	0.474	.637
Mean (SD) number of BPD criteria	1.90 (1.32)	1.71 (0.76)	0.357	.722
Fulfilling ≥5 BPD criteria[d]	5 (7)	0 (0)	0.483	.487
Suicidality			0.055	.973
Low	34 (45)	3 (43)		
Moderate	19 (25)	2 (29)		
High	24 (32)	2 (29)		
Ever received inpatient care, yes	2 (3)	0 (0)	0.183	.666
Previous counselling, yes	50 (65)	4 (57)	0.170	.680
Any ongoing psychopharmacological medication, yes[e]	28 (36)	3 (43)	0.116	.733
Ongoing counselling at inclusion, yes	55 (71)	6 (86)	0.659	.417
Number of months in ongoing counselling, mean (SD)	5.14 (5.85)	8.17 (6.62)	1.189	.239

Abbreviations: ADHD, attention-deficit hyperactivity disorder; BDD, body dysmorphic disorder; BPD, borderline personality disorder; CBT, cognitive behavioral therapy; IERITA, Internet-delivered emotion regulation individual therapy for Adolescents; TAU, treatment as usual; NSSI, nonsuicidal self-injury; OCD, obsessive-compulsive disorder.

[a] Assessed by the research team using the MINI-KID International Neuropsychiatric Interview and the Body Dysmorphic Disorder Questionnaire (administered as an interview).

[b] Includes both combined, primarily inattentive, and primarily hyperactive-impulsive subtype.

[c] Includes anorexia nervosa and bulimia nervosa.

[d] Assessed by the research team using the Structured Clinical Interview for DSM-IV.

[e] Classes of psychopharmacological medication were based on World Health Organization anatomic therapeutic chemical categories. See eTable 13 for breakdown of substances included in each group.

© 2023 Bjureberg J et al. *JAMA Network Open.*

eTable 5. Study and Participant Characteristics of Participants with Complete Data and Missing Data on Clinician-Rated Primary Outcome at Primary End-Point in TAU-only Condition

	Complete data	Missing data	Chi2/T	*P*
**No. (%)**	77 (94)	5 (6)		
**Study Characteristics**				
Site			0.653	.721
Skåne	32 (42)	3 (60)		
Stockholm	23 (30)	1 (20)		
Västra Götaland	22 (29)	1 (20)		
Source of referral			1.235	.266
Clinician	42 (55)	4 (80)		
Self	35 (46)	1 (20)		
**Participant Characteristics**				
Gender			0.346	.841
Female	72 (94)	5 (100)		
Male	2 (3)	0 (0)		
Non-binary	3 (4)	0 (0)		
Age, mean (SD)	15.04 (1.9)	14.75 (1.17)	0.516	.607
Any failed grades (yes)	13 (16.9)	3 (60.0)	5.558	.018
**Participant Clinical Characteristics**				
Age NSSI onset, mean (SD),	12.57 (1.57)	11.60 (1.34)	1.352	.180
Years since NSSI onset, mean (SD)	2.46 (1.28)	3.15 (1.53)	1.142	.257
Comorbidity[a]				
Major depressive disorder	43 (56)	5 (100)	3.772	.052
Anxiety disorders				
Social anxiety disorder	21 (27)	2 (40)	0.377	.539
Panic disorder/Agoraphobia	10 (13)	1 (20)	0.199	.656
Specific phobia disorder	13 (17)	0 (0)	1.003	.317
Generalized anxiety disorder	8 (10)	1 (20)	0.444	.505
ADHD[b]	15 (20)	0 (0)	1.192	.275
Autism spectrum disorder	2 (3)	1 (2)	4.034	.045
OCD/BDD	7 (9)	0 (0)	0.497	.481
Eating disorder[c]	1 (1)	0 (0)	0.066	.798
Oppositional defiant disorder	2 (3)	0 (0)	0.133	.715
Mean (SD) number of co-occurring disorders	1.74 (1.38)	2.20 (2.17)	0.696	.488
Mean (SD) number of BPD criteria	2.11 (1.53)	2.00 (2.00)	0.164	.871
Fulfilling ≥5 BPD criteria[d]	6 (8)	1 (20)	0.896	.344
Suicidality			0.618	.734
Low	35 (46)	2 (40)		
Moderate	19 (25)	2 (40)		
High	23 (30)	1 (20)		
Ever received inpatient care, yes	2 (3)	0 (0)	0.133	.715
Previous counselling, yes	51 (66)	2 (40)	1.413	.234
Any ongoing psychopharmacological medication, yes[e]	24 (31)	1 (20)	0.276	.599
Ongoing counselling at inclusion, yes	51 (66)	4 (80)	0.403	.526
Number of months in ongoing counselling, mean (SD)	4.94 (4.98)	12.75 (15.52)	2.478	.016

Abbreviations: ADHD, attention-deficit hyperactivity disorder; BDD, body dysmorphic disorder; BPD, borderline personality disorder; CBT, cognitive behavioral therapy; IERITA, Internet-delivered emotion regulation individual therapy for Adolescents; TAU, treatment as usual; NSSI, nonsuicidal self-injury; OCD, obsessive-compulsive disorder.

[a] Assessed by the research team using the MINI-KID International Neuropsychiatric Interview and the Body Dysmorphic Disorder Questionnaire (administered as an interview).

[b] Includes both combined, primarily inattentive, and primarily hyperactive-impulsive subtype.

[c] Includes anorexia nervosa and bulimia nervosa.

[d] Assessed by the research team using the Structured Clinical Interview for DSM-IV.

[e] Classes of psychopharmacological medication were based on World Health Organization anatomic therapeutic chemical categories. See eTable 13 for breakdown of substances included in each group.

© 2023 Bjureberg J et al. *JAMA Network Open.*

eTable 6. Between Condition Differences in Study and Participant Characteristics for Participants with Complete Data and Missing Data on Clinician-Rated Primary Outcome at Primary End-Point

	IERITA+TAU (n = 84)	TAU (n = 82)	Chi 2 / T	*P*
**Study Characteristics**				
Site				
Skåne				
No missing	32 (42)	35 (45)	0.859	.651
Missing	3 (60)	3 (43)	0.686	.710
Stockholm				
No missing	23 (30)	25 (32)		
Missing	1 (20)	3 (43)		
Västra Götaland				
No missing	22 (29)	17 (22)		
Missing	1 (20)	1 (14)		
Source of referral				
Clinician				
No missing	42 (55)	51 (66)	2.198	.138
Missing	4 (80)	4 (57)	0.686	.408
Self				
No missing	35 (46)	26 (34)		
Missing	1 (20)	3 (43)		
**Participant Characteristics**				
Gender				
Female				
No missing	72 (94)	70 (91)	1.514	.469
Missing	5 (100)	7 (100)		
Male				
No missing	2 (3)	5 (6)		
Missing	0 (0)	0 (0)		
Non-binary				
No missing	3 (4)	2 (3)		
Missing	0 (0)	0 (0)		
Age, mean (SD)				
No missing	15.04 (1.20)	15.01 (1.29)	0.128	.899
Missing	14.75 (1.17)	15.42 (1.57)	0.800	.442
Any failed grades (yes)				
No missing	13 (17)	9 (12)	0.849	.357
Missing	3 (60)	2 (29)	1.185	.276
**Participant Clinical Characteristics**				
Age NSSI onset, mean (SD)				
No missing	12.57 (1.57)	12.73 (1.17)	0.699	.458
Missing	11.60 (1.34)	12.43 (2.23)	0.736	.478
Years since NSSI onset, mean (SD)				
No missing	2.46 (1.28)	2.28 (1.23)	0.896	.372
Missing	3.15 (1.53)	2.99 (2.41)	0.131	.898
Comorbidity[a]				
Major depressive disorder				
No missing	43 (56)	44 (57)	0.026	.871
Missing	5 (100)	5 (71)	1.7143	.190
Anxiety disorders				
Social anxiety disorder				
No missing	21 (27)	23 (30)	0.127	.721
Missing	2 (40)	1 (14)	1.029	.310
Panic disorder/Agoraphobia				
No missing	10 (13)	15 (19)	1.194	.275
Missing	1 (20)	2 (29)	0.114	.735
Specific phobia disorder				
No missing	13 (17)	12 (16)	0.948	.827
Missing	0 (0)	2 (29)	1.714	.190

© 2023 Bjureberg J et al. *JAMA Network Open.*

eTable 6. Between Condition Differences in Study and Participant Characteristics for Participants with Complete Data and Missing Data on Clinician-Rated Primary Outcome at Primary End-Point (continued)

	IERITA+TAU (n = 84)	TAU (n = 82)	Chi 2 / T	*P*
Generalized anxiety disorder				
No missing	8 (10)	12 (16)	0.919	.338
Missing	1 (20)	0 (0)	1.527	.217
ADHD[b]				
No missing	15 (19)	13 (17)	0.175	.676
Missing	0 (0)	1 (14)	0.779	.377
Autism spectrum disorder				
No missing	2 (3)	4 (5)	0.694	.405
Missing	1 (20)	0 (0)	1.527	.217
OCD/BDD				
No missing	7 (9)	3 (4)	1.711	.191
Missing	0 (0)	0 (0)		
Eating disorder[c]				
No missing	1 (1)	6 (8)	3.742	.053
Missing	0 (0)	0 (0)		
Oppositional defiant disorder				
No missing	2 (3)	3 (4)	0.207	.649
Missing	0 (0)	0 (0)		
Mean (SD) number of co-occurring disorders				
No missing	1.74 (1.38)	1.88 (1.70)	0.572	.568
Missing	2.20 (2.17)	1.57 (1.13)	0.659	.525
Mean (SD) number of BPD criteria				
No missing	2.12 (1.52)	1.89 (1.32)	0.961	.338
Missing	2.00 (2.00)	1.71 (0.76)	0.350	.734
Fulfilling ≥5 BPD criteria[d]				
No missing	6 (8)	5 (6)	0.098	.754
Missing	1 (20)	0 (0)	1.527	.217
Suicidality				
Low				
No missing	35 (45)	34 (44)	0.358	.982
Missing	2 (40)	3 (43)	0.206	.902
Moderate				
No missing	19 (25)	19 (25)		
Missing	2 (40)	2 (29)		
High				
No missing	23 (30)	24 (31)		
Missing	1 (20)	2 (29)		
Ever received inpatient care, yes				
No missing	2 (3)	2 (3)	0.000	1.000
Missing	0 (0)	0 (0)		
Previous counselling, yes				
No missing	51 (66)	50 (65)	0.029	.865
Missing	2 (40)	4 (57)	0.343	.558
Any ongoing psychopharmacological medication, yes[e]				
No missing	24 (31)	28 (36)	0.465	.496
Missing	1 (20)	3 (43)	0.686	.408
Ongoing counselling at inclusion, yes				
No missing	51 (66)	55 (71)	0.484	.486
Missing	4 (80)	6 (86)	0.069	.793
Number of months in ongoing counselling, mean (SD)				
No missing	4.94 (4.99)	5.14 (5.85)	0.191	.849
Missing	12.75 (15.52)	8.17 (6.62)	0.655	.531

© 2023 Bjureberg J et al. *JAMA Network Open.*

eTable 6. Between Condition Differences in Study and Participant Characteristics for Participants with Complete Data and Missing Data on Clinician-Rated Primary Outcome at Primary End-Point (continued)

Abbreviations: ADHD, attention-deficit hyperactivity disorder; BDD, body dysmorphic disorder; BPD, borderline personality disorder; CBT, cognitive behavioral therapy; IERITA, Internet-delivered emotion regulation individual therapy for Adolescents; TAU, treatment as usual; NSSI, nonsuicidal self-injury; OCD, obsessive-compulsive disorder.
[a] Assessed by the research team using the MINI-KID International Neuropsychiatric Interview and the Body Dysmorphic Disorder Questionnaire (administered as an interview).
[b] Includes both combined, primarily inattentive, and primarily hyperactive-impulsive subtype.
[c] Includes anorexia nervosa and bulimia nervosa.
[d] Assessed by the research team using the Structured Clinical Interview for DSM-IV.
[e] Classes of psychopharmacological medication were based on World Health Organization anatomic therapeutic chemical categories. See eTable 13 for a breakdown of substances included in each group.

© 2023 Bjureberg J et al. *JAMA Network Open.*

eTable 7. Study and Participant Characteristics of Participants Missing ≤20% versus >20% of Weekly Measures in IERITA+TAU Condition

	Total ≤20%missing	Total > 20%missing	Chi2/ T	*P*
**No. (%)**	63 (75)	21 (25)		
**Study Characteristics**				
Site			0.300	.861
Skåne	29 (46)	9 (43)		
Stockholm	20 (32)	8 (38)		
Västra Götaland	14 (22)	4 (19)		
Source of referral			0.158	.691
Clinician	42 (67)	13 (62)		
Self	21 (33)	8 (38)		
**Participant Characteristics**				
Gender			1.271	.530
Female	58 (92)	19 (91)		
Male	3 (5)	2 (10)		
Non-binary	2 (3)	0 (0)		
Age, mean (SD)	15.16 (1.29)	14.70 (1.32)	1.399	.083
Any failed grades (yes)	6 (10)	5 (24)	2.82	.093
**Participant Clinical Characteristics**				
Age NSSI onset, mean (SD)	12.83 (1.25)	12.33 (1.28)	1.553	.124
Years since NSSI onset, mean (SD)	2.33 (1.26)	2.37 (1.64)	0.100	.923
Comorbidity[a]				
Major depressive disorder	34 (54)	15 (71)	1.980	.160
Anxiety disorders				
Social anxiety disorder	17 (27)	7 (33)	0.311	.577
Panic disorder/Agoraphobia	13 (21)	4 (19)	0.025	.875
Specific phobia disorder	12 (19)	2 (10)	1.029	.310
Generalized anxiety disorder	9 (14)	3 (14)	0.000	1.000
ADHD[b]	12 (19)	2 (10)	1.029	.310
Autism spectrum disorder	2 (3)	2 (10)	1.400	.237
OCD/BDD	3 (5)	0 (0)	1.037	.309
Eating disorder[c]	5 (8)	1 (5)	0.239	.625
Oppositional defiant disorder	2 (3)	1 (5)	0.115	.734
Mean (SD) number of co-occurring disorders	1.84 (1.57)	1.90 (1.95)	0.151	.880
Mean (SD) number of BPD criteria	1.75 (1.29)	2.29 (1.19)	1.687	.096
Fulfilling ≥5 BPD criteria[d]	3 (5)	2 (10)	0.638	.424
Suicidality			3.639	.162
Low	30 (48)	7 (33)		
Moderate	17 (27)	4 (19)		
High	30 (48)	7 (33)		
Ever received inpatient care, yes	2 (3)	0 (0)	0.683	.409
Previous counselling, yes	40 (63)	14 (67)	0.069	.793
Any ongoing psychopharmacological medication, yes[e]	24 (38)	7 (33)	0.153	.695
Ongoing counselling at inclusion, yes	45 (71)	16 (76)	0.180	.672
Number of months in ongoing counselling, mean (SD)	6.07 (6.20)	4.31 (5.08)	1.016	.314

Abbreviations: ADHD, attention-deficit hyperactivity disorder; BDD, body dysmorphic disorder; BPD, borderline personality disorder; CBT, cognitive behavioral therapy; IERITA, Internet-delivered emotion regulation individual therapy for Adolescents; TAU, treatment as usual; NSSI, nonsuicidal self-injury; OCD, obsessive-compulsive disorder.

[a] Assessed by the research team using the MINI-KID International Neuropsychiatric Interview and the Body Dysmorphic Disorder Questionnaire (administered as an interview).

[b] Includes both combined, primarily inattentive, and primarily hyperactive-impulsive subtype.

[c] Includes anorexia nervosa and bulimia nervosa.

[d] Assessed by the research team using the Structured Clinical Interview for DSM-IV.

[e] Classes of psychopharmacological medication were based on World Health Organization anatomic therapeutic chemical categories. See eTable 13 for a breakdown of substances included in each group.

© 2023 Bjureberg J et al. *JAMA Network Open.*

eTable 8. Study and Participant Characteristics of Participants missing ≤20% versus >20% of Weekly Measures in TAU-only Condition

	Total ≤20%missing	Total >20%missing	Chi2/ T	*P*
**No. (%)**	68 (83)	14 (17)		
**Study Characteristics**				
Site			1.854	.396
Skåne	31 (46)	4 (29)		
Stockholm	18 (26)	6 (43)		
Västra Götaland	19 (28)	4 (29)		
Source of referral			0.255	.614
Clinician	39 (57)	7 (50)		
Self	29 (43)	7 (50)		
**Participant Characteristics**				
Gender			2.151	.341
Female	64 (94)	13 (93)		
Male	1 (1)	1 (7)		
Non-binary	3 (4)	0 (0)		
Age, mean (SD)	15. 04 (1.21)	14.93 (1.15)	0.318	.751
Any failed grades (yes)	11 (16)	5 (36)	2.822	.093
**Participant Clinical Characteristics**				
Age NSSI onset, mean (SD),	12.50 (1.60)	12.57 (1.45)	0.154	.878
Years since NSSI onset, mean (SD)	2.54 (1.25)	2.35 (1.60)	0.477	.635
Comorbidity[a]				
Major depressive disorder	39 (57)	9 (59)	0.230	.632
Anxiety disorders				
Social anxiety disorder	19 (28)	3 (29)	0.002	.962
Panic disorder/Agoraphobia	7 (10)	4 (29)	3.339	.068
Specific phobia disorder	10 (15)	3 (21)	0.393	.531
Generalized anxiety disorder	7 (10)	2 (14)	0.189	.663
ADHD[b]	11 (16)	4 (29)	1.193	.275
Autism spectrum disorder	1 (1)	2 (14)	5.409	.020
OCD/BDD	7 (10)	0 (0)	1.576	.209
Eating disorder[c]	1 (1)	0 (0)	0.208	.648
Oppositional defiant disorder	2 (3)	0 (0)	0.422	.516
Mean (SD) number of co-occurring disorders	1.68 (1.40)	2.21 (1.53)	1.291	.201
Mean (SD) number of BPD criteria	2.00 (1.39)	2.64 (2.10)	1.432	.156
Fulfilling ≥5 BPD criteria[d]	4 (6)	3 (21)	3.594	.058
Suicidality			1.047	.593
Low	31 (46)	6 (43)		
Moderate	16 (24)	5 (36)		
High	21 (31)	3 (21)		
Ever received inpatient care, yes	2 (3)	0 (0)	0.422	.516
Previous counselling, yes	44 (25)	9 (64)	0.001	.976
Any ongoing psychopharmacological medication, yes[e]	22 (32)	3 (21)	0.654	.419
Ongoing counselling at inclusion, yes	47 (69)	8 (57)	0.754	.385
Number of months in ongoing counselling, mean (SD)	4.72 (5.08)	8.63 (11.10)	1.640	.107

Abbreviations: ADHD, attention-deficit hyperactivity disorder; BDD, body dysmorphic disorder; BPD, borderline personality disorder; CBT, cognitive behavioral therapy; IERITA, Internet-delivered emotion regulation individual therapy for Adolescents; TAU, treatment as usual; NSSI, nonsuicidal self-injury; OCD, obsessive-compulsive disorder.

[a] Assessed by the research team using the MINI-KID International Neuropsychiatric Interview and the Body Dysmorphic Disorder Questionnaire (administered as an interview).

[b] Includes both combined, primarily inattentive, and primarily hyperactive-impulsive subtype.

[c] Includes anorexia nervosa and bulimia nervosa.

[d] Assessed by the research team using the Structured Clinical Interview for DSM-IV.

[e] Classes of psychopharmacological medication were based on World Health Organization anatomic therapeutic chemical categories. See eTable 13 for breakdown of substances included in each group.

© 2023 Bjureberg J et al. *JAMA Network Open.*

eTable 9. Between Condition Differences in Study and Participant Characteristics for Participants missing ≤20% and >20% of Weekly Measures

	IERITA+TAU (n = 84)	TAU (n = 82)	Chi 2 / T	*P*
**Study Characteristics**				
Site				
Skåne				
≤20% missing	29 (46)	31 (46)	0.740	.692
>20% missing	9 (43)	4 (29)	0.843	.656
Stockholm				
≤20% missing	20 (32)	18 (26)		
>20% missing	8 (38)	6 (43)		
Västra Götaland				
≤20% missing	14 (22)	19 (28)		
>20% missing	4 (19)	4 (29)		
Source of referral				
Clinician				
≤20% missing	42 (67)	39 (57)	1.202	.273
>20% missing	13 (62)	7 (50)	0.486	.486
Self				
≤20% missing	21 (33)	29 (43)		
>20% missing	8 (38)	7 (50)		
**Participant Characteristics**				
Gender				
Female				
≤20% missing	58 (94)	64 (94)	1.306	.520
>20% missing	19 (90)	13 (93)	0.061	.805
Male				
≤20% missing	3 (5)	1 (1)		
>20% missing	2 (10)	1(7)		
Non-binary				
≤20% missing	2 (3)	3 (4)		
>20% missing	0 (0)	0 (0)		
Age, mean (SD)				
≤20% missing	15.16 (1.29)	15.03 (1.21)	0.554	.580
>20% missing	14.70 (1.32)	14.93 (1.15)	0.519	.607
Any failed grades (yes)				
≤20% missing	6 (10)	11 (16)	1.282	.258
>20% missing	5 (24)	5 (36)	0.583	.445
**Participant Clinical Characteristics**				
Age NSSI onset, mean (SD)				
≤20% missing	12.83 (1.25)	12.50 (1.60)	1.291	.199
>20% missing	12.33 (1.28)	12.57 (1.45)	0.511	.613
Years since NSSI onset, mean (SD)				
≤20% missing	2.33 (1.26)	2.54 (1.25)	0.932	.353
>20% missing	2.36 (1.64)	2.35 (1.60)	0.023	.982
Comorbidity[a]				
Major depressive disorder				
≤20% missing	34 (54)	39 (57)	0.152	.697
>20% missing	15 (71)	9 (64)	0.199	.656
Anxiety disorders				
Social anxiety disorder				
≤20% missing	17 (27)	19 (28)	0.015	.902
>20% missing	7 (33)	4 (29)	0.088	.766
Panic disorder/Agoraphobia				
≤20% missing	13 (21)	7 (10)	2.703	.100
>20% missing	4 (19)	4 (29)	0.432	.511
Specific phobia disorder				
≤20% missing	12 (19)	10 (15)	0.441	.507
>20% missing	2 (10)	3 (21)	0.972	.324

© 2023 Bjureberg J et al. *JAMA Network Open.*

eTable 9. Study and Participant Characteristics of Participants missing ≤20% versus >20% of Weekly Measures in TAU-only Condition (continued)

	IERITA+TAU (n = 84)	TAU (n = 82)	Chi 2 / T	*P*
Generalized anxiety disorder				
≤20% missing	9 (14)	7 (10)	0.486	.486
>20% missing	3 (14)	2 (14)	0.000	1.00
ADHD[b]				
≤20% missing	12 (19)	11 (16)	0.186	.666
>20% missing	2 (10)	4 (29)	2.146	.143
Autism spectrum disorder				
≤20% missing	2 (3)	1 (1)	0.434	.515
>20% missing	2 (10)	2 (14)	0.188	.664
OCD/BDD				
≤20% missing	3 (5)	7 (10)	1.420	.233
>20% missing	0 (0)	0 (0)	-	-
Eating disorder[c]				
≤20% missing	5 (8)	1 (1)	3.128	.077
>20% missing	1 (3)	0 (0)	0.686	.407
Oppositional defiant disorder				
≤20% missing	2 (3)	2 (3)	0.006	.938
>20% missing	1 (5)	0 (0)	0.686	.407
Mean (SD) number of co-occurring disorders				
≤20% missing	1.84 (1.57)	1.68 (1.40)	0.636	.526
>20% missing	1.90 (1.95)	2.21 (1.53)	0.500	.620
Mean (SD) number of BPD criteria				
≤20% missing	1.75 (1.29)	2.00 (1.39)	1.079	.283
>20% missing	2.29 (1.19)	2.64 (2.10)		
Fulfilling ≥5 BPD criteria[d]				
≤20% missing	3 (5)	4 (6)	0.081	.776
>20% missing	2 (10)	3 (21)	0.972	.325
Suicidality				
Low				
≤20% missing	30 (48)	31 (46)	0.532	.766
>20% missing	7 (33)	6 (43)	2.664	.264
Moderate				
≤20% missing	17 (27)	16 (24)		
>20% missing	4 (19)	5 (36)		
High				
≤20% missing	16 (25)	21 (31)		
>20% missing	10 (48)	3 (21)		
Ever received inpatient care, yes				
≤20% missing	2 (3)	2 (3)	0.006	.938
>20% missing	0 (0)	0 (0)	-	-
Previous counselling, yes				
≤20% missing	40 (63)	44 (65)	0.021	.885
>20% missing	14 (67)	9 (64)	0.021	.884
Any ongoing psychopharmacological medication, yes[e]				
≤20% missing	24 (38)	22 (32)	0.473	.491
>20% missing	7 (33)	3 (21)	0.583	.445
Ongoing counselling at inclusion, yes				
≤20% missing	45 (71)	47 (69)	0.084	.773
>20% missing	16 (76)	8 (57)	1.414	.234
Number of months in ongoing counselling, mean (SD)				
≤20% missing	6.07 (6.20)	4.72 (5.08)	1.139	.258
>20% missing	4.31 (5.08)	8.63 (11.10)	1.32	.200

eTable 9. Study and Participant Characteristics of Participants missing ≤20% versus >20% of Weekly Measures in TAU-only Condition (continued)

© 2023 Bjureberg J et al. *JAMA Network Open.*

Abbreviations: ADHD, attention-deficit hyperactivity disorder; BDD, body dysmorphic disorder; BPD, borderline personality disorder; CBT, cognitive behavioral therapy; IERITA, Internet-delivered emotion regulation individual therapy for Adolescents; TAU, treatment as usual; NSSI, nonsuicidal self-injury; OCD, obsessive-compulsive disorder.
[a] Assessed by the research team using the MINI-KID International Neuropsychiatric Interview and the Body Dysmorphic Disorder Questionnaire (administered as an interview).
[b] Includes both combined, primarily inattentive, and primarily hyperactive-impulsive subtype.
[c] Includes anorexia nervosa and bulimia nervosa.
[d] Assessed by the research team using the Structured Clinical Interview for DSM-IV.
[e] Classes of psychopharmacological medication were based on World Health Organization anatomic therapeutic chemical categories. See eTable 13 for breakdown of substances included in each group.

© 2023 Bjureberg J et al. *JAMA Network Open.*

eTable 10: Model Comparison by AIC and BIC for Count Outcomes at 1-Month Post-Treatment and 3-Month Post-treatment

Count outcome	AIC			BIC		
	Poisson	ZIP	ZINB	Poisson	ZIP	ZINB
**Blinded assessor-rated NSSI (DSHI-Y)**	7162.66	7061.44	7036.77	7190.66	7092.56	7071.00
**Self-reported self-destructive behaviors (BSL Supplement)**	7126.91	7124.41	*	7154.92	7155.53	*

Abbreviations: AIC Akaike information criterion, BIC Bayesian information criterion, ZIP Zero-inflated Poisson, ZINB Zero-inflated negative-binomial

* Message from R: A value greater than 22000 has been detected for the shape/size parameter of the negative binomial distribution. This typically indicates that the Poisson model would be better.

eTable 11. Treatment Credibility/Expectancy, Satisfaction, Treatment Completion, and Therapist Time Spent on Treatment in Internet-delivered Emotion Regulation Individual Therapy for Adolescents

	IERITA+TAU		
	n	Mean	SD
CEQ			
Credibility	83	20.0	4.1
Expectancy	83	61.0	20.2
CSQ	79	25.2	5.2
Completed modules adolescents[a]	84	9.6	2.2
Completed modules parents[b]	84	5.5	1.0
Therapist time spent in minutes adolescents[c]	84	306	139.8
Therapist time spent in minutes parents[c]	84	111.4	43.2

Abbreviations: CEQ, Credibility/Expectancy Questionnaire; CSQ, Client Satisfaction Questionnaire; IERITA, Internet-delivered emotion regulation individual therapy for adolescents.

[a] Total number of modules were 11.
[b] Total number of modules were 6.
[c] Including time spent on reviewing participant exercises and providing written feedback.

© 2023 Bjureberg J et al. *JAMA Network Open.*

eTable 12. Descriptive Statistics for Self-reported Nonsuicidal Self-Injury Frequency Week 0 Through 16

	IERITA+TAU		TAU	
	**Mean (SD)**	**Median (Q1, Q3)**	**Mean (SD)**	**Median (Q1, Q3)**
Self-reported NSSI (DSHI-Y)				
Week 0	3.2 (3.7)	2.00 (1.00, 5.00)	3.1 (4.0)	2.00 (1.00, 4.00)
Week 1	3.0 (3.9)	2.00 (0.00, 4.00)	2.2 (2.4)	1.00 (0.00, 3.00)
Week 2	2.4 (3.3)	1.00 (0.00, 3.00)	2.2 (2.6)	2.00 (0.00, 3.00)
Week 3	2.6 (4.0)	1.00 (0.00, 3.00)	2.1 (2.5)	1.00 (0.00, 4.00)
Week 4	2.2 (3.7)	1.00 (0.00, 3.00)	2.0 (2.1)	2.00 (0.00, 3.00)
Week 5	1.9 (3.1)	1.00 (0.00, 2.00)	1.7 (2.5)	1.00 (0.00, 3.00)
Week 6	1.9 (3.1)	1.00 (0.00, 2.00)	1.7 (2.1)	1.00 (0.00, 3.00)
Week 7	1.8 (3.5)	0.00 (0.00, 2.00)	1.9 (2.8)	1.00 (0.00, 2.00)
Week 8	1.8 (3.7)	0.00 (0.00, 2.00)	1.6 (2.2)	1.00 (0.00, 2.00)
Week 9	1.7 (3.1)	0.00 (0.00, 2.00	1.7 (2.3)	1.00 (0.00, 2.00)
Week 10	1.4 (3.2)	0.00 (0.00, 1.00)	1.4 (2.3)	0.00 (0.00, 2.00)
Week 11	1.3 (3.5)	0.00 (0.00, 1.00)	1.4 (2.4)	0.50 (0.00, 2.00)
Week 12	1.1 (2.5)	0.00 (0.00, 1.00)	1.7 (2.9)	0.00 (0.00, 3.00)
Week 13	0.9 (2.3)	0.00 (0.00, 1.00)	1.5 (2.4)	0.00 (0.00, 2.00)
Week 14	0.8 (1.6)	0.00 (0.00, 1.00)	1.5 (2.6)	0.00 (0.00, 2.00)
Week 15	0.8 (2.4)	0.00 (0.00, 1.00)	1.5 (2.7)	0.00 (0.00, 2.00)
Week 16	0.9 (2.3)	0.00 (0.00, 1.00)	1.4 (2.7)	0.00 (0.00, 2.00)

Abbreviations: IERITA, Internet-delivered Emotion Regulation Individual Therapy; TAU, Treatment as Usual; DSHI-Y, Deliberate Self-Harm Inventory – Youth Version, NSSI, nonsuicidal self-injury.

© 2023 Bjureberg J et al. *JAMA Network Open.*

eTable 13. List of Psychopharmacological Medications Included in The Medication Classes Reported in Table 1 and Table 2

Name of group/subgroups (ATC code)	Name of substance (ATC code)
Antidepressants (N06A)	
SSRI (N06AB)	Fluoxetine (N06AB03), Sertralin (N06AB06), Citalopram (N06AB04), Escitalopram (N06AB10)
Other antidepressants (N06AX)	Venlafaxine (N06AX16)
Anxiolytics (N05B)	
Diphenylmethane derivatives (N05BB)	Hydroxyzine (N05BB01)
Benzodiazepine derivatives (N05BA)	Diazepam (N05BA01)
Hypnotics and sedatives (N05C)	
Melatonin receptor agonists (N05CH)	Melatonin (N05CH01)
Other hypnotics and sedatives (N05CM)	Propiomazine (N05CM06)
Antihistamines for systemic use (R06A)	Promethazine (R06AD02), Alimemazine (R06AD01)
Psychostimulants, agents used for ADHD and nootropics (N06B)	Methylphenidate (N06BA04), Atomoxetine (N06BA09), Lisdexamfetamine (N06BA12)
Antiadrenergic agents, centrally acting (C02A)	Guanfacine (C02AC02)
Antipsychotics (N05A)	Quetiapine (N05AH04)
Antiepileptics (N03A)	Lamotrigine (N03AX09)
Beta blocking agents (C07A)	Propranolol (C07AA05)

Abbreviations: SSRI, Selective serotonin reuptake inhibitors.

© 2023 Bjureberg J et al. *JAMA Network Open.*

eFigure 1. Screenshot of an Interactive Work Sheet from Internet-delivered Emotion Regulation Individual Therapy for Adolescents

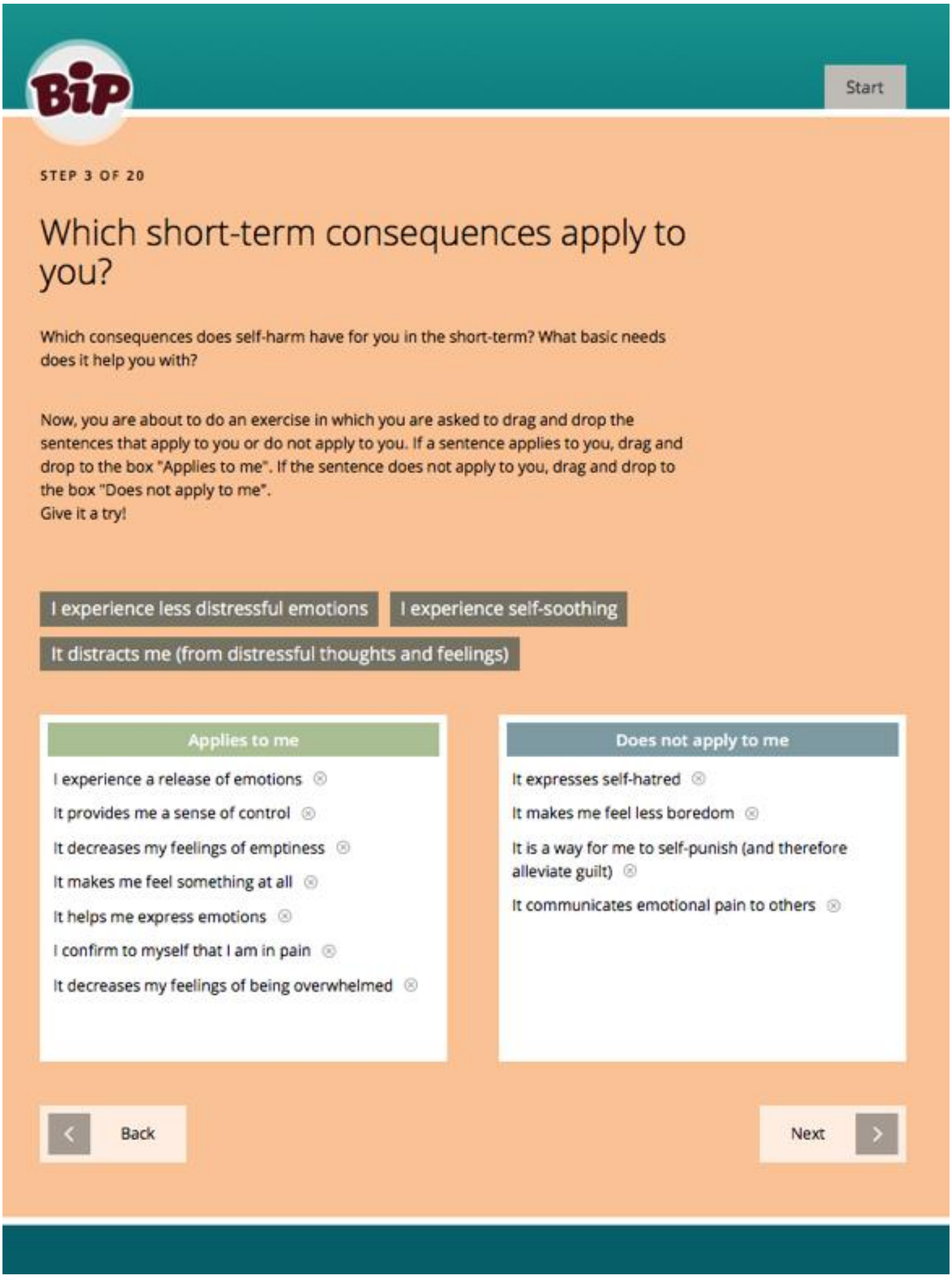

In: Bjureberg, J., et al. (2018). Extending research on Emotion Regulation Individual Therapy for Adolescents (IERITA) with nonsuicidal self-injury disorder: open pilot trial and mediation analysis of a novel online version. BMC Psychiatry, 18(1), 1–13. Reproduced with permission.

eFigure 2. Screenshot of a Worksheet with Psychoeducational Text and Illustrations from Internet-delivered Emotion Regulation Individual Therapy for Adolescents

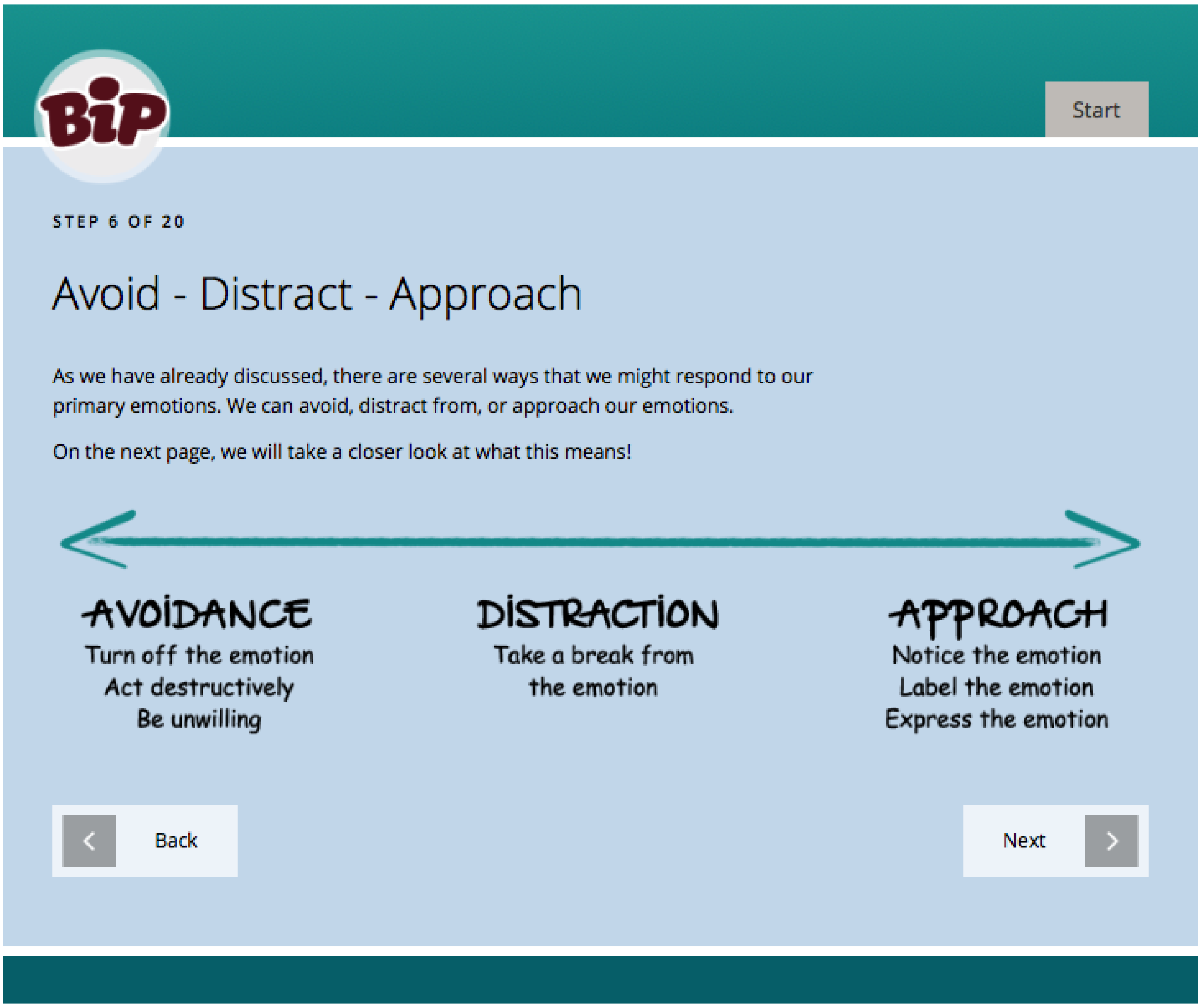

In: Bjureberg, J., et al. (2018). Extending research on Emotion Regulation Individual Therapy for Adolescents (ERITA) with nonsuicidal self-injury disorder: open pilot trial and mediation analysis of a novel online version. BMC Psychiatry, 18(1), 1–13. Reproduced with permission.

© 2023 Bjureberg J et al. *JAMA Network Open.*

eFigure 3. Screenshot of a Worksheet with Psychoeducational Text and Illustrations from Internet-delivered Emotion Regulation Individual Therapy for Adolescents

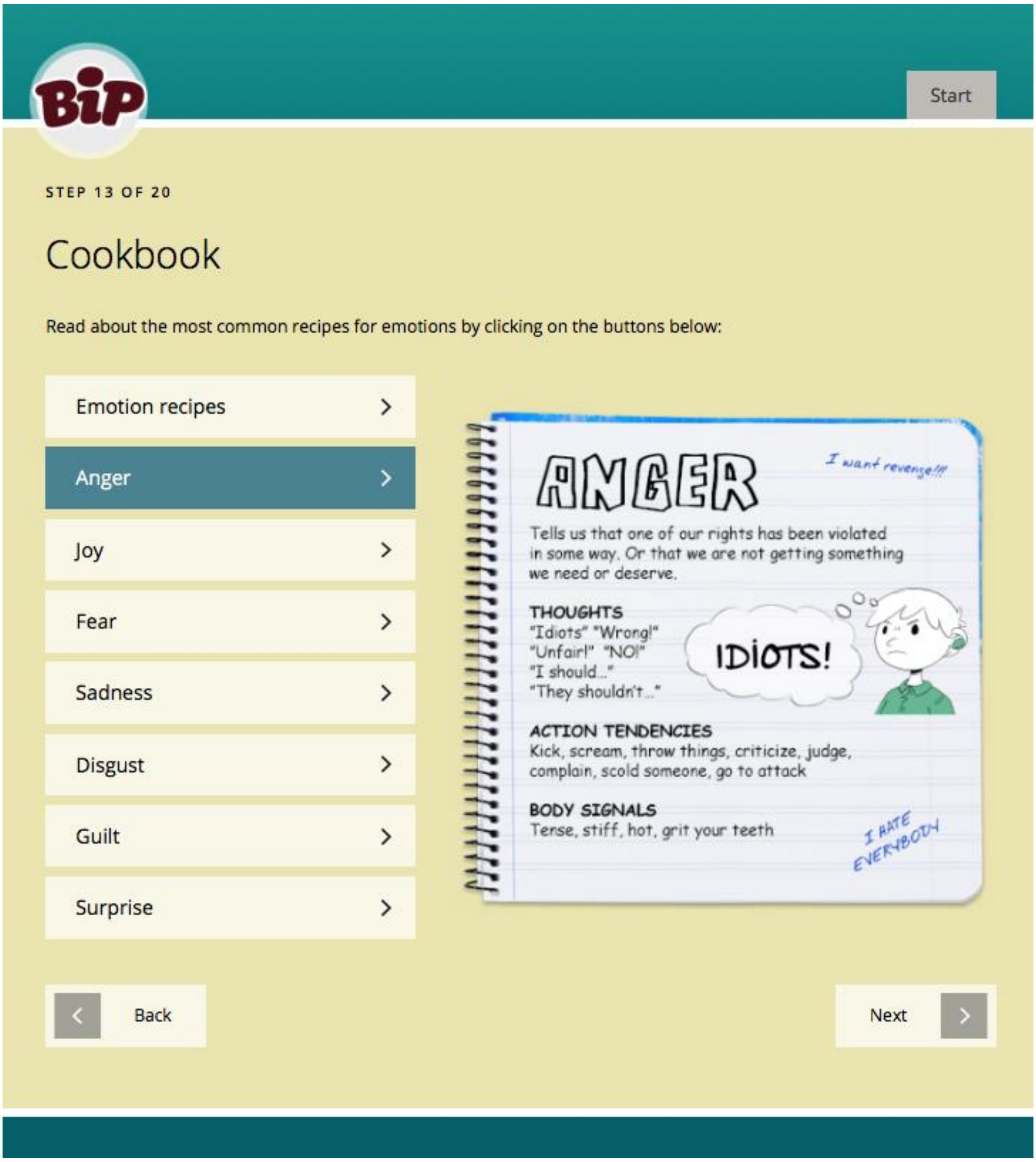

In: Bjureberg, J., et al. (2018). Extending research on Emotion Regulation Individual Therapy for Adolescents (ERITA) with nonsuicidal self-injury disorder: open pilot trial and mediation analysis of a novel online version. BMC Psychiatry, 18(1), 1–13. Reproduced with permission.

© 2023 Bjureberg J et al. *JAMA Network Open.*

eFigure 4. Screen Shot of Mobile App from Internet-delivered Emotion Regulation Individual Therapy for Adolescents

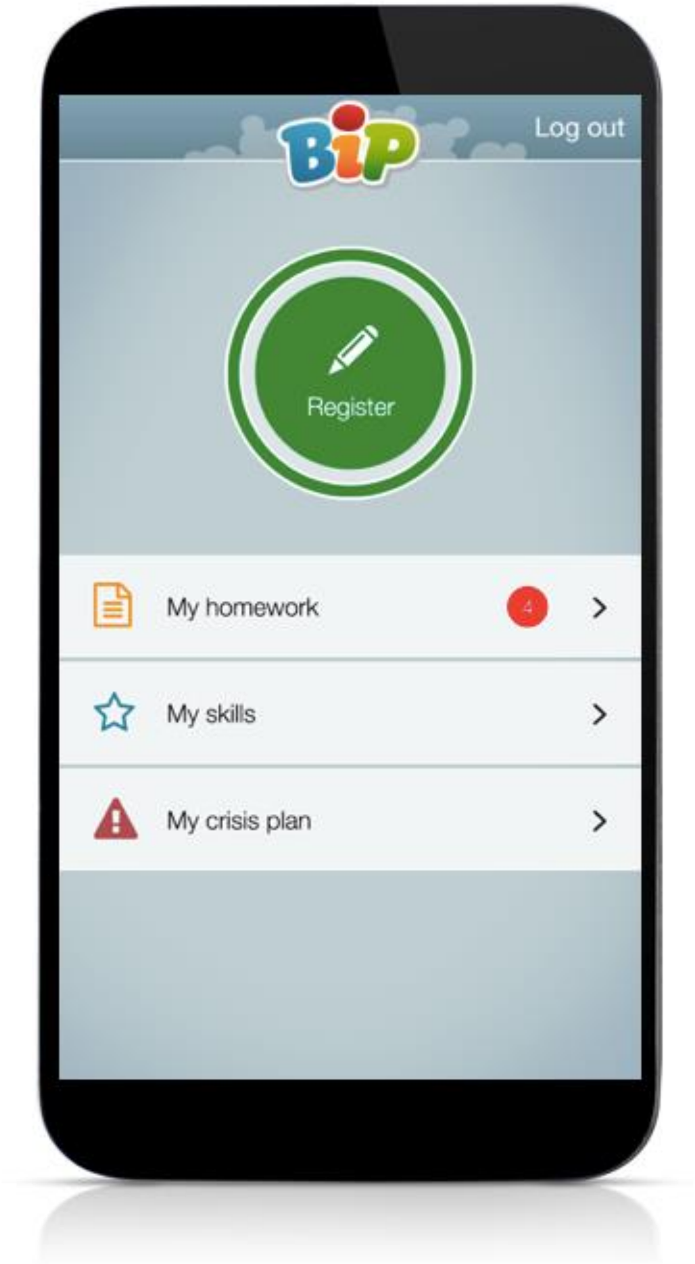

In: Bjureberg, J., et al. (2018). Extending research on Emotion Regulation Individual Therapy for Adolescents (ERITA) with nonsuicidal self-injury disorder: open pilot trial and mediation analysis of a novel online version. BMC Psychiatry, 18(1), 1–13. Reproduced with permission.

© 2023 Bjureberg J et al. *JAMA Network Open.*

eFigure 5. Missing Observation Occurrence Pattern For All Individuals (A) and By Treatment (B)

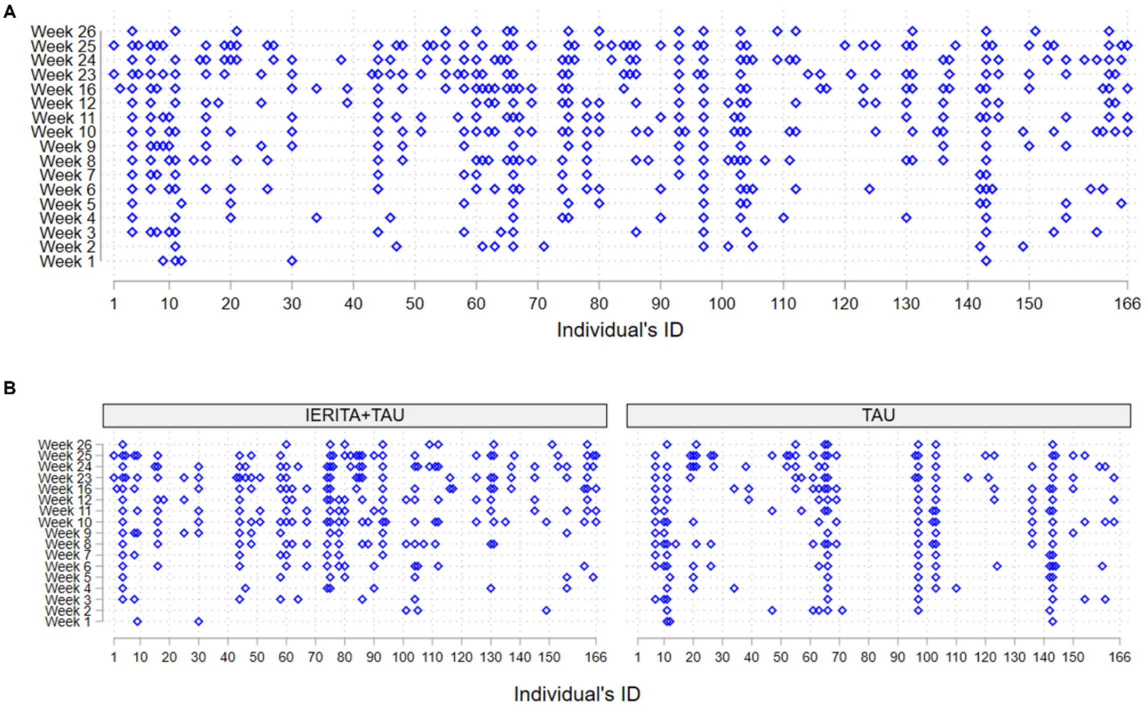

Each column corresponds to an individual. There were no missing observations in Week 0.

© 2023 Bjureberg J et al. *JAMA Network Open.*

eFigure 6. Histogram Over Nonsuicidal Self-Injury (NSSI) Across Week

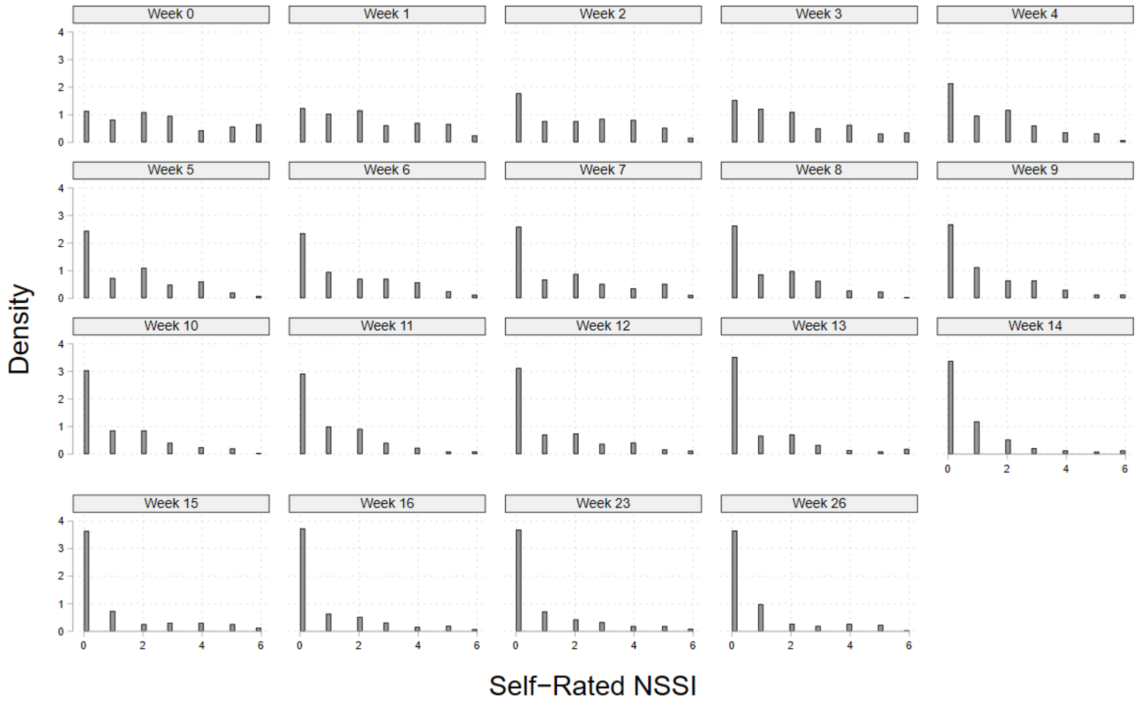

© 2023 Bjureberg J et al. *JAMA Network Open.*

eFigure 7. Illustration of the Key Elements of the Parallel Process Growth Model for Mediation Employed in the Current Study

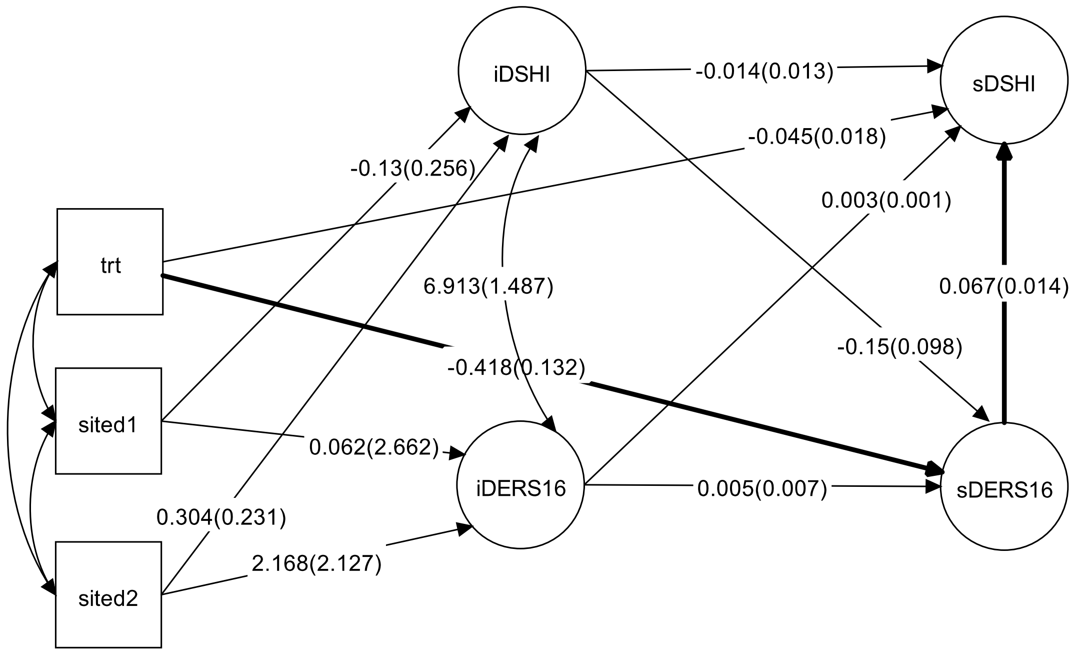

Parallel process growth model for mediation examining the treatment effect on the growth rate (log count rate) of the outcome Deliberate Self-Harm Inventory – Youth version (DSHI-Y) via the growth rate of the process variable Difficulties in Emotion Regulation Scale – 16-item version (DERS-16). A zero-inflated negative binomial latent growth model and a continuous latent growth model were estimated for DSHI-Y and DERS-16, respectively. Observed indicator variables measured over time and zero-inflated part of the model are not shown. The bolded arrows specify the tested mediation pathway; trt= binary treatment variable (IERITA+TAU = 1, TAU= 0); sited1, sited2=dummy variables for sites; iDSHI= latent intercept factor (initial status) for the DSHI-Y; sDSHI =latent growth rate factor for the DSHI-Y; iDERS = latent intercept factor (initial status) for the DERS-16; sDERS-16 = latent growth rate factor for the DERS-16. Numbers represent unstandardized estimates, with bootstrapped standard errors in parentheses.

© 2023 Bjureberg J et al. *JAMA Network Open.*

eFigure 8. Panel A: Scatterplot of Subject-specific Latent Growth Rates of the Mediator and Outcome During Treatment. Panel B: Bootstrap Distribution of the Indirect Effect

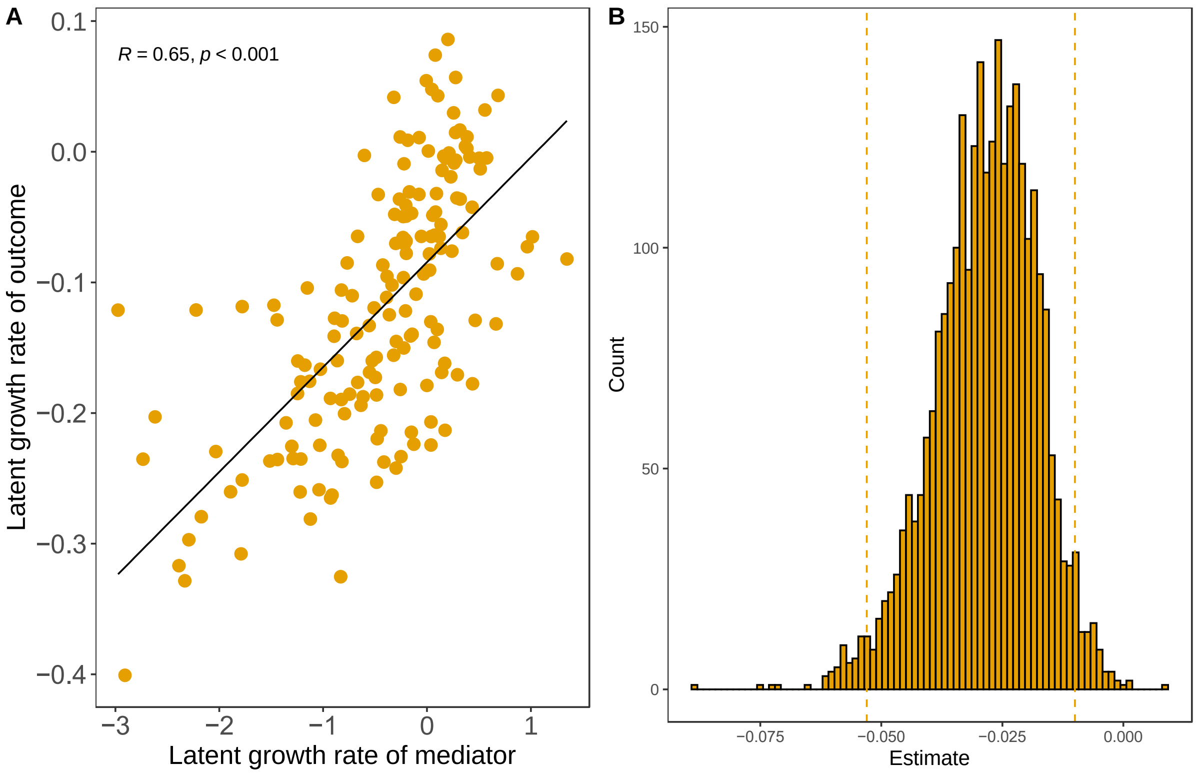

The dotted vertical lines represent 95% upper and lower bound.

© 2023 Bjureberg J et al. *JAMA Network Open.*

eFigure 9. Sensitivity Analysis of Estimated Mediated Effect

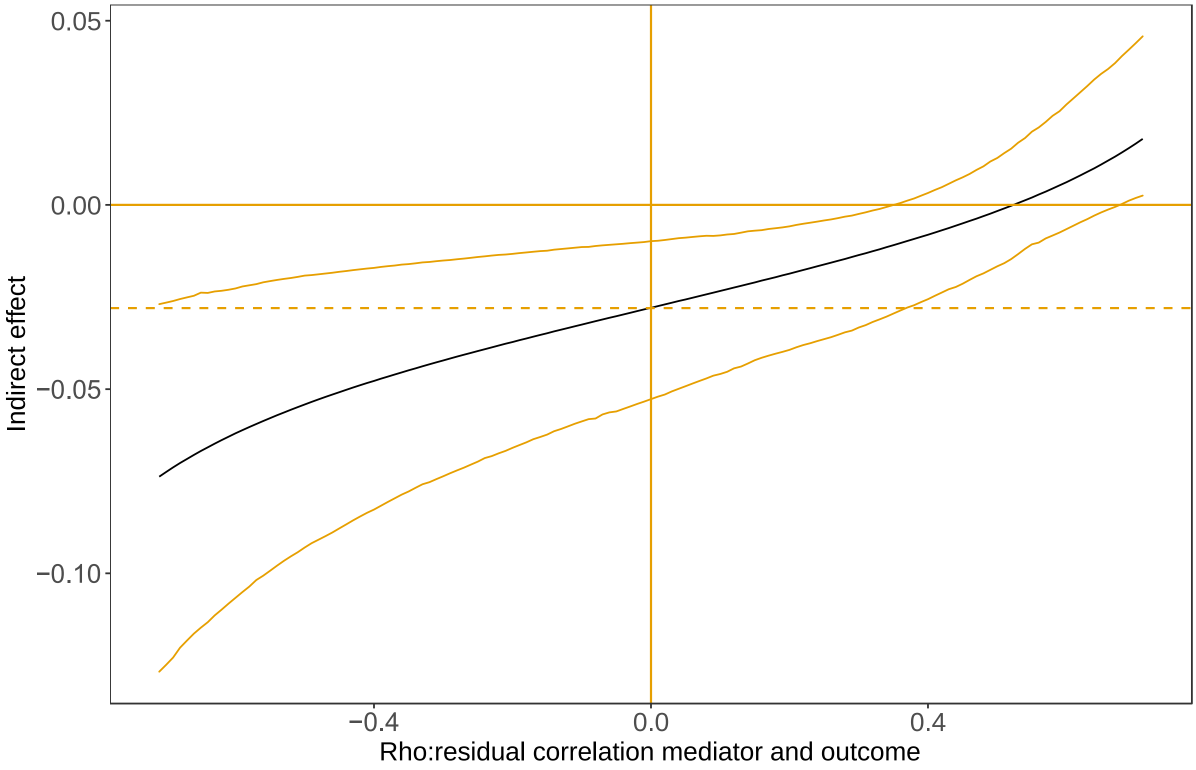

The estimated values of the indirect (mediated) effect as a function of the sensitivity parameter (Rho), which represents the correlation between the error terms in the mediator and the outcome growth models. The bold black line and yellow bands represent the point estimates of the indirect effect and 95% bootstrapped confidence intervals, respectively.

© 2023 Bjureberg J et al. *JAMA Network Open.*
