# Supplementary material for: Effect of Internet-Delivered Emotion Regulation Individual Therapy for Adolescents With Nonsuicidal Self-Injury Disorder: A Randomized Clinical Trial
Source: JAMA Netw Open. 2023 Jul 13;6(7):e2322069. doi: 10.1001/jamanetworkopen.2023.22069 (PMC10346121; doi:10.1001/jamanetworkopen.2023.22069)
Supplement: Supplement 2. — Trial Protocol [file jamanetwopen-e2322069-s002.pdf]

Trial Protocol

A Randomized Controlled Trial of Internet-delivered Emotion Regulation Individual Treatment for Adolescents with Non-suicidal Self-injury

BACKGROUND

Non-suicidal self-injury (NSSI; also referred to as self-harm) is a common and serious global health problem and refers to direct and deliberate destruction of one's own body tissue in the absence of suicidal intent[1], such as cutting or burning oneself. These behaviors are prevalent in a variety of adolescent populations, ranging from approximately 14% to 45%.[2-4] NSSI commonly begins between the ages of 13 and 16 years.[3,5]

Repeated NSSI often results in negative emotional and interpersonal consequences for both the afflicted youths and their families. Self-harm behavior is listed in the DSM-IV as a criterion of borderline personality disorder (BPD), however many individuals who engage in repeated self-harm do not fulfill criteria for this disorder.[6-8] NSSI has high comorbidity with a range of psychiatric disorders, in particular depression and anxiety disorders.[6] Therefore, a NSSI diagnosis was included in DSM-5 in the section "conditions for further study".[9] A Swedish study found that 6.7% of an adolescent community sample fulfilled the proposed diagnostic criteria for NSSI.[3]

Several studies indicate that NSSI is a risk factor for suicide attempt or completed suicide.[10,11] Longitudinal findings have shown that NSSI predicts suicide attempts even after controlling for a history of suicidal behavior.[11,12] Although these findings are highly alarming, deaths by suicide most frequently occur during adulthood. Thus, although suicidal behavior often has its onset in adolescence, adulthood is the period with the greatest risk for death by suicide.[13,14] Consequently, early effective interventions for adolescents who engage in NSSI could potentially have a suicide preventive effect.[15] However, although NSSI is a serious condition, many individuals who engage in self-harm behaviors do not seek help.[16] The stigma associated with NSSI is thought to be an important barrier to treatment-seeking.[15]

EVIDENCE-BASED TREATMENTS FOR NSSI

To date no established treatments specific to self-harm exists.[15] Randomized controlled trials (RCT) of psychological treatment effectiveness for NSSI are scarce and the results are inconclusive. It is of great concern that few therapeutic treatments have been designed and evaluated specifically for NSSI and even less research has focused on treatment for adolescents that engage in NSSI. Most studies that have been conducted in the field have included individuals that engage in self-harm behaviors both with and without suicide intent.[17] Failing to distinguish lethal intent and the usage of vague and inconsistent terms and definitions makes it difficult to understand what exactly the different treatments have been addressing. With that said, there is some support that psychological interventions can reduce frequency of NSSI.[15] Among the treatments that have been most frequently studied for self-harm is dialectical behavioral therapy (DBT).[18,19] DBT is effective in reducing self-harm for individuals with BPD.[20] However, DBT aims at reducing symptoms of BPD, irrespectively of self-harm, and is comprehensive and time-consuming with high costs (approximately two years of treatment including both individual therapy and skills training in group). Therefore less intensive, shorter and less expensive psychological interventions have been called for.[21]

EMOTION REGULATION GROUP THERAPY (ERGT)

ERGT, a form of cognitive behavioral therapy (CBT), was developed to address the need for an effective short-term, focused and easily implemented treatment for adults with repeated self-harm behavior.[22] Several researchers and treatment models (e.g. DBT) have emphasized the central role of emotion regulation in the development and maintenance of self-harm behaviors, a theory that is gaining robust scientific support.[18,23,24] The 14-week long ERGT intervention is administrated adjunctive to treatment as usual and aims at reducing NSSI for self-harming individuals by improving emotion regulation. A newly conducted RCT on ERGT for adult women with borderline personality

disorder and self-harming behaviors showed significant effects on NSSI and other self-destructive behaviors, borderline personality disorder-symptoms, depression, anxiety, and emotion dysregulation.[25] These findings are consistent with previous studies evaluating ERGT.[22,26] The main findings from these studies were replicated in a Swedish multi-center effectiveness study conducted by this research group.[27] Recently, an adolescent adaption of ERGT was developed by our research group; Individual Emotion Regulation Therapy for Adolescents (ERITA; EPN dnr 2014/1-31/3), which in contrast to ERGT, is given in an individual format. In this pilot study (N = 17), ratings of treatment credibility/expectancy, therapeutic alliance and the treatment completion rate were satisfactory. Intent-to-treat analyses revealed significant improvements associated with large effect sizes in past-month NSSI frequency, NSSI versatility, emotion regulation difficulties, self-destructive behaviors, and global functioning from pre- to post-treatment. Further, with the exception of NSSI versatility, all of these improvements were either maintained or further improved upon at 6-month follow-up. Finally, change in emotion regulation difficulties mediated improvements in NSSI over the course of treatment. To conclude, results suggest the acceptability, feasibility, and utility of this treatment for adolescents with NSSI-D.[28]

Internet-delivered CBT

Guided internet-delivered CBT (ICBT) has been shown to be highly effective for several mental disorders, including depression and anxiety disorders for both adults[29,30] and adolescents.[31] In ICBT, the patient follows an online treatment manual and receives support and guidance from a trained professional through online communication. While ICBT is probably as efficacious as face-to-face CBT[32,33], it allows for less therapist time per patient, it eliminates the effects of geographical distance between therapist and patient, and patients do not need to schedule appointments during their work or school day. Furthermore, ICBT can increase accessibility to psychological treatment and individuals with stigmatizing illnesses are more likely to use the internet than traditional health care to seek help[34], suggesting that ICBT may be suitable for NSSI. Given the advantages of ICBT, ERITA was recently adapted to be delivered over the internet; Internet delivered Emotion Regulation Therapy for Adolescents (iERITA). iERITA is currently being evaluated in an open pilot study by our research group (EPN: dnr 2015/1895-31/5). This treatment consists of two parts: one for the adolescents and one for the parents. To date, all participants (N=25) have completed treatment. Three- and 6-month follow-up assessments are still being collected. So far, 19, and 15 participants, have completed the three- and six-month follow-up measure, respectively. Also, eight of the participants in the iERITA pilot study have been included in a qualitative interview sub-study. Preliminary results of the pilot study are presented in the section “Preliminary Results”.

AIMS AND OBJECTIVES

The overall objective of the present study is to evaluate the efficacy of adding iERITA to treatment as usual (TAU), compared to treatment as usual only. Further objectives are to investigate the possible mechanisms of change, prognostic factors, and to evaluate to cost-effectiveness of the treatment. These are the specific research questions:

- Is participation in iERITA in addition to treatment as usual, more effective than treatment as usual only, in regards to reducing NSSI, other self-destructive behaviors, emotion dysregulation, acceptance, suicidal thoughts and behaviors, depression, stress, and anxiety, for adolescents with repeated NSSI?
- Is participation in the parent program in addition to treatment as usual, more effective than treatment as usual only, in regards to enhancing the parent’s perceived ability to cope with their child’s negative emotions?
- Is iERITA associated with improved emotion regulation and attachment? If so, does improved emotion regulation mediate treatment outcome, and is attachment a potential competing mediator?
- Is it possible to identify predictive factors (e.g. age, NSSI frequency, BPD-symptoms, childhood trauma and psychiatric comorbidity) for treatment outcome?

- Can iERITA imply economic advantages?
- In which context (i.e. circumstances, antecedents, and consequences) does NSSI occur in, when reported in real time?

METHOD

DESIGN

This study adopts a randomized controlled trial (RCT) design. The study has an active control group (i.e. continuing ongoing treatment or initiating treatment as usual). The study is conducted on several Child and Adolescent Mental Health Outpatient Services (CAMHS), spread over three regions in Sweden (Stockholm, region Västra Götaland, and region Skåne). The study includes three sets of follow-up measures; post, three, and 12 months after treatment completion. The control group is measured directly after their 12 weeks of TAU, and at three-month follow-up. After they complete the three-month follow-up they are offered participation in iERITA.

RECRUITMENT

Participants are recruited from the whole country, through advertisements in daily press, social media and information directed to primary health care specialized for child and adolescents with psychiatric problems, CAMHS, and the school health care. The inclusion and exclusion criteria are assessed through clinician administered interviews and assessments, with clinicians experienced in assessing and treating adolescents with NSSI and suicidal ideation. Once the adolescents and his/her parents, have shown interest in participating, they are allocated to the participating CAHMS closest to their home for inclusion assessment.

INCLUSION CRITERIA

Adolescents, 13-17 years old that fulfill criteria for NSSI according to clinician-administered non-suicidal self-injury disorder index[35] and have engaged in NSSI during the past month. One parent needs to commit to participate in the parent program.

EXCLUSION CRITERIA

Primary psychosis, severe suicidal ideation, bipolar disorder type I, ongoing substance dependence, or comorbid axis-I-disorders (i.e. anorexia nervosa or post-traumatic stress disorder) that are considered primary and that require immediate treatment, or life circumstances that interfere with treatment (i.e. on-going abuse), or pronounced language skill deficits and learning difficulties (since the treatment format assumes normal reading and writing skills).

MATERIAL

TREATMENT MANUAL

The treatment is an internet-delivered intervention (iERITA) based on the face-to-face ERITA treatment manual (EPN dnr 2014/1-31/3) and further developed after experience gained from the ongoing pilot of the internet-delivered version of the intervention (EPN: dnr 2015/1895-31/5). The treatment consists of two parts: one for the adolescents and one for the parents.

The treatment for the adolescents comprises 11 weekly modules and emphasizes the following themes: (a) the potentially paradoxical effects of emotional avoidance, (b) the emotion-regulating effect of emotional acceptance/willingness and (c) the importance of controlling behaviors when emotions are present, rather than controlling emotions themselves. Detailed individual behavior analysis is made for each individual and instructions are given on how to increase emotional awareness and gradually decrease fear of emotions and widen the behavioral repertoire. The material is accessed through a secure Internet platform where the adolescents will conduct daily registrations (of NSSI and protective factors). The adolescents will be encouraged to use a mobile app to assist them in their daily training. The mobile application also includes an individual crisis list with instructions

about who to contact in case of psychiatric deterioration (e.g. increased suicidal ideation). These features will also be available on the secure Internet platform for adolescents without access to a smart phone.

At least one parent[1] for each adolescent will be required to participate in a parent program administrated through the Internet. The parent program will be administered in parallel to the adolescents' treatment. The parent program comprises written psychoeducation and exercises on how to support the adolescent's increased use of emotion regulation and interpersonal skills.

Therapist contact

Throughout treatment, adolescents and parents have regular contact with an assigned therapist through an Internet platform, where the treatment content is also presented. The therapist provides feedback, assists in planning homework assignments, and is available for questions at any time during treatment. Text-messages to the participants will be used as reminders to work with the treatment. The therapists are licensed psychologists, psychologists under training or licensed psychotherapists with experience in treating adolescents with NSSI. All therapists undergo a mandatory four-day education where they receive information about the treatment model/manual, therapeutic stance and the Internet format. Throughout the treatment, therapists receive supervision from the research team experienced in treating adolescents with internet-delivered ERITA.

The treatment team at each CAHMS (therapists and psychiatrists) will have continuous treatment conferences with the research group during treatment, to discuss aspects of the clinical care of the participants and the treatment. The research team could consult the treatment team, but the CAHMS where the internet-therapist work is responsible for the participants' health care and security.

Mobile application

The internet platform, mobile app and the text function have been implemented using secure technology and have previously been approved by the ethics committee for several previous projects by members of this research group (dnr: 2015/1722-31; EPN dnr 2015/969-31; 2014/673-31/2; 2014/1885-31). Several security measures have been implemented with regards to the smartphone app:

- Access to the smartphone app requires a password
- A participant's account is locked to one smartphone and cannot be accessed from other smartphones
- Participant are signed out from the app after a short period of inactivity

Measures

Data, in the form of self-assessments, will be collected for all participants at baseline (weekly for four weeks) preceding the face-to-face assessment, pre-treatment, weekly during treatment, post-treatment, weekly for four weeks after treatment termination, and weekly for four weeks at three-months follow-up. Additional follow-up for participants that are randomized to iERITA will be collected 12 months after treatment is terminated (see attached flow diagram). Thenceforth, the participants will be offered the possibility to answer the same measures once every year for five years. Assessments are administered via the Internet and answers (also results of the clinical interviews) are securely stored on a database developed for this purpose and have been used in several completed and ongoing studies (dnr: 2011/1816 31/1, 2010/1993-31/4, 2011/2027-31/5, 2012/1995-31/1, 2013/1321-31/3, 2014/1-31/3, 2014/673-31/2; 2014/1885-31, 2015/1722-31, 2015/1895-31/5, and 2015/969-31). All data traffic is encrypted (2048 bit SSL) ensuring a high security level.

[1] The term "parents" refers to legal guardian(s) of the adolescent.

To measure the effect of the treatments and to screen for NSSI and psychiatric comorbidity, the following measures will be used (for a measurement chart, see figure 1):

1. SCREENING MEASURES

FREQUENCY AND SEVERITY OF NSSI

Clinician-Administered Non-Suicidal Self-Injury Disorder Index (CANDI)[35]

PSYCHIATRIC COMORBIDITY

Mini-Kid Internationell Neuropsykiatrisk Intervju Svensk version 6 (MINI KID)[36], Body Dysmorphic Disorder Questionnaire (administered as an interview; BDDQ)[37] and The Autism-Tics, AD/HD and other Comorbidities Inventory (A-TAC)[38]

SUICIDAL IDEATION

The suicidality module included in the MINI KID[36]

SYMPTOMS OF BORDERLINE PERSONALITY DISORDER

Structured Clinical Interview for DSM-IV Axis II Personality Disorders Borderline Personality Disorder Module (SCID-II-BPD)[.39]

GLOBAL FUNCTIONING:

Childrens Global Assessment Scale (CGAS)[40]

SYMPTOM SEVERITY AND IMPROVEMENT:

The Clinical Global Impressions —Severity and Improvement scales (CGI-S[41] och CGI-I[42])

2. PRIMARY OUTCOME MEASURE:

SELF-HARMING BEHAVIOR

The Deliberate Self-harm Inventory – youth version (DSHI-Y)[43]

3. SECONDARY OUTCOME MEASURES:

EMOTION REGULATION

Difficulties in Emotion Regulation Scale (DERS)[44], and Difficulties in Emotion Regulation Scale – Short form (DERS-16)[45]

OTHER SELF-DESTRUCTIVE BEHAVIORS AND BORDERLINE SYMTOMS

Borderline Symptom List Behavior – Behavior Supplement (BSL-Supplement)[46], and Borderline Personality Features in Childhood (BPFS-C).[47]

ANXIETY, DEPRESSION, STRESS, AND SUICIDALITY

Depression, Anxiety and Stress Scale (DASS-21)[48], and Suicidal Ideation Questionnaire (SIQ-JR).[49]

LIFE QUALITY

Kidscreen-10[50]

DEGREE OF ACCEPTANCE TOWARDS EMOTIONS

Acceptance and Action Questionnaire (AAQ-II)[51]

DEGREE OF ANXIOUS AND AVOIDANT ATTACHMENT TOWARDS PARENT

Short form of the Experience in Close Relationships Scale – Revised Child version [52]

4. BASELINE MEASURE

SLEEP

Insomnia Severity Index (ISI)[53]

5. TREATMENT PROCESS VARIABLES:

Working Alliance Inventory–short form (WAI)[54], the Credibility/Expectancy Questionnaire (CEQ)[55], the Client Satisfaction Questionnaire (CSQ)[56], the Adverse Event Protocol (AEP)[57], and the clinician rated ICBT adherence scale (PIAS)[58]

6. PARENT MEASURES:

PARENT VARIABLES

History of NSSI and Suicidal Ideation (SITBI-SF-SH)[59] and the Coping with Children's Negative Emotions Scale Adolescent Version (CCNES-A).[60]

7. HEALTH ECONOMICS

Trimbos and Institute of Technology Cost Questionnarie for Psychiatry (TIC-P)[61]

POWER

The sample size of this study was determined based on the observed treatment effects in the uncontrolled pilot study of i ERITA (EPN: dnr 2015/1895-31/5). Based on an expected average difference of two self-harming episodes between the groups at post-treatment yields a total sample size of 140 participants with a power of 82% given alpha .05, after the 12-week follow-up. Given an estimated attrition rate of 15%, we aim to include 165 adolescents.

The power refers to a 0.05-level Wald test for the interaction between a binary treatment and linear time in a zero-inflated negative binomial regression model at a 0.05 level. The power was estimated with 200 bootstrap samples. The data for the treated group were obtained by sampling with replacement from the available pilot data. The data for the control group was obtained by adding the difference to the count of a bootstrap sample from the iERITA pilot data on the 25 treated patients.

PROCEDURE

The study will be hosted by the research clinic of the Child and Adolescent Mental Health care (BUP-CPF) in Stockholm. This unit is a collaboration between CAHMS and Centre for Psychiatry Research (Centrum för psykiatriforskning). Three CAHMS over the country will be hosting the participants.

Adolescents with repeated NSSI, and their parents, will receive information about the study through information directed to CAMHS, primary health care specialized for child and adolescents with psychiatric problems, the school health care, and newspaper and social media advertisement. There are three ways in which the family can be recruited to the study: (1) if the adolescent and his/her parents have contact with a clinician and show preliminary interest in participating in the study, the treating clinician can send a letter of referral to one of the hosting units; (2) the parents can notify interest to participate in the study through self-referral; (3) clinicians at the hosting units recommend families to the designated contact at that unit. In either case, a clinician in the research group or at one of the hosting units, will call the adolescent and parents and examine overall inclusion/exclusion criteria. If it is apparent that the participant does not fulfill inclusion criteria (e.g. has not engaged in repeated NSSI), or fulfills exclusion criteria, the participant will not be invited to the assessment interview. If the youth is in need of some other kind treatment, he/she will receive advice and/or referral to adequate treatment. If the adolescent currently fulfills inclusion criteria, he/she is instructed to log on to a secure website and complete four weekly assessments. These assessments include questions regarding NSSI, other self-destructive behaviors, emotions regulation and attachment. The purpose of these assessments is to confirm that the adolescent is actively engaging in self-harm behavior and to establish a baseline measurement of NSSI severity. After the fourth assessment, the adolescent and his/her parents will undergo a structured face-to-face assessment by a clinician experienced in assessing and treating adolescents with self-harm behavior and suicidal ideation. This includes an assessment of the adolescents' mental health status and the inclusion/exclusion criteria consisting of evaluation of psychiatric comorbidity and suicidal ideation (MINI-KID and BDDQ), borderline personality disorder (SCID-II BPD), functional impairment (CGAS), self-harm behaviors as well as presence or absence of the Nonsuicidal self-injury diagnosis (CANDI). In addition to these

measures, questions about the adolescents' home and school situation will be asked. The adolescent will also get recommendation/referral to other adequate treatment, based on the assessment, independent of ongoing treatment or not.

If a participant and/or his/her parents are not interested in participating, the routines for regular care will be followed (i.e. if the adolescent is not eligible for inclusion the family will be informed about the reasons for exclusion and the participant will receive adequate advice or referral).

Families eligible for inclusion into the study will be informed about the study in a way that is easy to understand for the participant and his/her parents and the right to not participate will be stressed. Participants and parents will be given both verbal and written information about the study, in which the details of the study are outlined and the contact information of the research group is presented, to enable direct contact with the researchers by telephone or e-mail.

When the adolescent is less than 15 years of age, written informed consent will be required from both adolescent's caregivers (if there are two) as well as verbal consent from the adolescent. If the adolescent is over 15 years of age and assessed as being adequately mature (i.e. at expected level of a 15-year old or older) only the parent that participates in the parent program is required to give written informed consent regarding his/her own participation. In these cases, the adolescent provides written informed consent.

If the adolescent and parent(s) consent to participation, they will receive information on how to log on to a secure website comprising pre-assessment and information on demographic variables for both the adolescent and his/her parents. Once the pre-assessment is completed, randomization is executed. The family could either be allocated to starting treatment immediately or start treatment 24 weeks later (when the intervention group has completed the three month follow-up). The treatment lasts for twelve weeks. Both groups are asked to undergo the post-assessment approximately twelve weeks after the pre-assessment. The post-assessment could either be done face-to-face, or via telephone. The follow-up measures at three, six, and twelve months are done via the phone. All assessments are done by the treating clinician (psychologists, psychologists under training or psychotherapists); the post- and follow-up measures are conducted by independent assessors.

Pre-, post and follow-up assessments for the adolescents

The pre- and post-assessments includes the structured clinician administrated assessment of the adolescent's functional impairment (CGAS), NSSI (CANDI and DSHI-Y), emotion dysregulation (DERS and DERS-16), borderline personality disorder symptomatology (BPFS-C) and other self-destructive behaviors (BSL-Supl), suicidal ideation (SIQ-JR), accepting stance (AAQ-2), depression, anxiety and stress (DASS-21), and client satisfaction (CSQ). At post and follow-up assessments questions regarding potential adverse events will be addressed (Adverse events). In addition to these scales the therapist will rate participant adherence during treatment (iiPAS).

Weekly measures will be administered four weeks before the face-to-face assessment, during treatment, and four weeks after treatment termination. Four weekly measures will also be administered at each follow-up. The weekly measures consist of four short self-report scales that measure emotion regulation (DERS-16), NSSI (DSHI-Y), other self-destructive behaviors (BSL-Supl), and attachment (ECR-RC). In addition to these scales the participant will be asked to report if there have been any changes in medication during the past week. At treatment week 1, treatment expectancy/credibility (CEQ) will be rated, and therapeutic alliance (WAI) will be rated at week 4. The participants will answer a maximum of 58 questions per week. For a measurement chart, see figure 1.

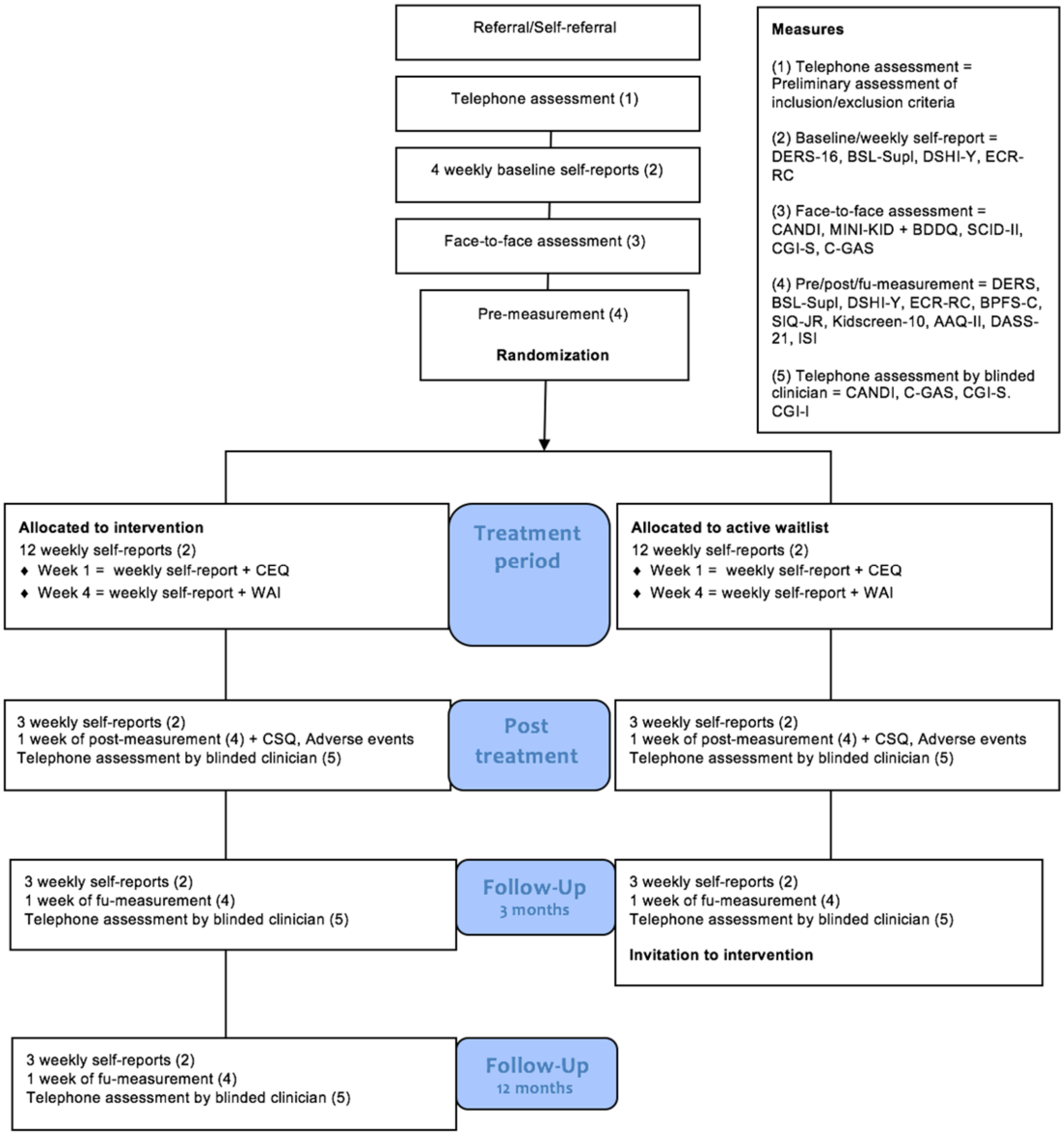

Figure 1. Chart over the different assessments and measures for both the intervention and waitlist group.

Pre-, post- and follow-up assessments for the parents

Parents will complete questionnaires about their own possible history of NSSI (SITBI-SF-SH), and about how they cope with their child's negative emotions (CCNES-A). At post, questions regarding potential adverse events will be addressed (Adverse events).

Preliminary results

As a first step in evaluating the feasibility, acceptability, and utility iERITA for adolescents with repeated NSSI, the research group has conducted an open pilot study including 25 adolescents with NSSID, aged 13-17 years (EPN dnr 2015/1895-31/5). To date, all adolescents have completed treatment, 24 participants have provided post-treatment data and so far, 19, and 15 participants,

respectively have completed the three- and six-month follow-up assessments. Preliminary analyses reveal statistically significant large effects on NSSI (standardized mean difference between pre- and post-treatment, d = 0.88), and moderate effects on emotion dysregulation (d = 0.77). At six-month follow-up, these improvements were either maintained or further improved upon, from pre-treatment with statistically significant large effects on NSSI (standardized mean difference between pre- and six-month follow-up, d = 1.28), and emotion dysregulation (d = 0.92). Ratings of treatment satisfaction, treatment credibility/expectancy and number of completed modules (median = 11; IQR=9-11) were high. No adverse events directly related to the treatment have been reported.

The research group has also interviewed a sub-sample of the participants (N = 8; dnr 2015/1895-31/5) about their experience of internet-delivered ERITA. Preliminary results of the qualitative analysis revealed three themes associated with the experience of iERITA: utility, support, and flexibility. Overall, a positive treatment experience was described. What was perceived as useful differed between participants, emphasizing the need of a broad treatment outline. Support was experienced as sufficient and the therapist's contribution was described as necessary for a positive experience. Further, the flexibility of the treatment format was experienced as both positive and negative. A high flexibility could, for some, put a lot of pressure on organization and responsibility, which was described as burdensome. On the other hand, the high flexibility made it easier for the participants to adjust the treatment work to other obligations and was time-saving. These findings were considered when the treatment manual was revised.

Statistical Analyses

Treatment effects will be evaluated according to the intention-to-treat principle. Primary end-point for all outcomes is post-treatment. Secondary end-point include a controlled 3 month follow-up.

Primary Analysis

The primary outcome analysis will include treatment group (ERITA , TAU) and weekly reports of NSSI frequency measured once every week 4 weeks prior to treatment start, once every week during treatment, and once every week four weeks after treatment termination. Regression analysis modelled for count data will be used to estimate trend over time. Four weeks after treatment termination will be considered primary endpoint. Pairwise contrasts (group x time interaction) from the regression model will be used to evaluate between-group differences at primary endpoint (4 weeks after treatment termination).

Secondary Analyses

Secondary outcomes measured once before, during, and after treatment will be analyzed in a similar fashion as for the primary outcome, modelled after data's distribution (count or continuous). Measures collected only once at baseline and post-treatment will be analyzed with regression analyses including treatment group (online ERITA , TAU) as the between-subjects factor, time (baseline to post) as the within-subjects factor, and group × time interactions, modelled after data's distribution (count or continuous).

Parallel process latent growth curve modeling will be used to determine whether change in week-to-week emotion dysregulation (DERS-16) during treatment mediate the overall effect of ERITA+ETAU on week-to-week change in the primary outcome self-rated NSSI frequency (DSHI-Y).

3 Month Follow-Up

Data collected at 3-month follow-up will be used to determine the extent to which potential treatment gains were maintained both within and between treatment conditions.

Time plan

The participants and their parents will be recruited and treated during winter 2017 until the end of 2019.

Previous experience of methods and access to relevant staff

ERGT (the equivalent treatment for adults) has been studied in the US and a total of 106 adult women have been treated. Two randomized controlled trials and one open trial of ERGT have been conducted

in the US with positive results[22,26,62]. No complications or ethical problems have been reported. In a recently completed Swedish trial[27], the research group behind this study has also investigated the effectiveness of ERGT for adult women with NSSI with promising results. The research group has also conducted a pilot trial of ERGT for adolescents (ERITA; dnr: 2014/1-31/3) with promising results[28] and a pilot trial of iERITA with preliminary promising results (see “Preliminary results” above; EPN dnr 2015/1895-31/5). The research group includes competences and experience that guarantees good quality regarding how the study will be planned and conducted.

Research group

Clara Hellner Gumpert, MD, PhD, Specialist and associate professor in child- and adolescent psychiatry. She has experience from psychiatric research regarding a range of different behavioral problems, such as NSSI and other destructive behaviors. Clara Hellner Gumpert is PI for the large study “Emotion Regulation Group Therapy (ERGT) for women with non-suicidal self-injury (NSSI) – a pilot study” (dnr: 2013/1321-31/3), the study “Emotion Regulation Individual Therapy for adolescents with repeated non-suicidal self-injury” (dnr: 2014/1-31/3), the study “Internet-delivered Emotion Regulation Individual Therapy for adolescents with repeated non-suicidal self-injury” (dnr 2015/1895-31/5), and has been project leader in several large projects with many actors.

Brjánn Ljótsson, psychologist, PhD. Involved in more than 30 completed intervention studies regarding psychological treatment for different psychological and somatic disorders. Brjánn Ljótsson has also been instrumental in the ERGT study for adult women with NSSI (dnr: 2013/1321-31/3), the ERITA study for adolescents with NSSI (dnr: 2014/1-31/3) and the iERITA study for adolescents with NSSI (dnr 2015/1895-31/5). Brjánn Ljótsson is a senior researcher in the “Child Internet Project” (BarnInternetProjektet, BiP) and is experienced conducting research with adolescents and their parents. BiP has received ethics committee approval to conduct research on treatment for children/adolescents with functional gastrointestinal disorders (dnr: 2011/1816 31/1), anxiety disorders (dnr: 2010/1993-31/4, 2011 2027-31/5) and obsessive-compulsive disorder (dnr: 2012/1995-31/1).

Erik Hedman, psychologist, PhD, Karolinska Institutet, has conducted several randomized controlled trials on CBT and internet based treatments. He has also conducted research on prognostic and prescriptive factors for psychological treatments and has conducted health economic evaluations of CBT.

Hanna Sahlin, PhD-candidate, psychologist, psychotherapist, specialist in clinical psychology with extensive experience of treatment, supervision and educating in ERGT, DBT, BPD and NSSI. Hanna Sahlin was the clinical expert and main clinical supervisor in the ERGT study for adult women with NSSI (dnr: 2013/1321-31/3), clinical supervisor in the ERITA study for adolescents with NSSI (dnr: 2014/1-31/3) and clinician/therapist in the iERITA study for adolescents with NSSI (dnr 2015/1895-31/5). She has co-authored both the Swedish translation of the ERGT-manual and the ERITA-manual.

Johan Bjureberg, PhD-candidate, psychologist with experience regarding treatment of NSSI, ERGT and DBT. Johan Bjureberg was one of the project coordinators in the ERGT study (dnr: 2013/1321-31/3), and has also co-authored the Swedish translation of the ERGT-manual and the ERITA-manual. He was clinical supervisor in the ERGT study for adult women with NSSI (dnr: 2013/1321-31/3) and the project coordinator, and therapist and supervisor in the face-to-face ERITA study for adolescents with NSSI (dnr: 2014/1-31/3), and in the internet-delivered ERITA study for adolescents with NSSI (dnr 2015/1895-31/5).

Anna Ohlis, PhD-candidate, MD, resident in child psychiatry in another project focusing on self-harm. Anna Ohlis assisted in assessment and monitoring of the participants in the study of iERITA (dnr 2015/1895-31/5). Dr. Ohlis works at a specialized unit for youth with self-harm and borderline personality traits, and has vast experience from assessment and treatment of youth with these problems.

Olivia Simonsson, Psychologist under supervision with experience in treating adolescents with internet-delivered ERITA. Within her master thesis, Olivia Simonsson conducted the qualitative interviews within the internet-delivered ERITA project (dnr 2015/1895-31/5). She will work as a project coordinator in the present study.

Overall, the project team has expertise in treatment for behavioral change as well as management of adolescents with repeated self-harm.

ETHICAL CONSIDERATIONS AND PARTICIPANTS' SAFETY

The potential risks and negative consequences with treatment participation could be several; for example, violation of personal integrity (both for the adolescent and their parents), lack of effect of treatment, deterioration in mental health status, extra burden in terms of repeated assessments, and delayed treatment start. In order to minimize these risks as much as possible the following measures will be taken:

Personal integrity: The participants and their families are given both verbal and written information about the study. There may be a risk that the adolescent feels forced to participate if their parents give their consent. This is prevented by clearly informing the adolescent that they can choose not to participate even if their parents want them to participate. The families can at any time terminate their participation in the study.

The adolescent's parents are asked to complete questionnaires about their own history of NSSI and their sense of parenting, which could be perceived as violation of the parents' personal integrity. It is therefore stressed that it is optional for the parents to answer the questionnaires and they are instructed about the purpose of these measurements. If serious mental health problems are detected, the parent will receive recommendations for help seeking.

Patient integrity is ensured by following Socialstyrelsen's regulations regarding strong authentication for online communication and sensitive information.

Deterioration in mental health: As seen in the previous pilot studies on ERITA for adolescents, incidents outside of treatment may occur that will worsen the mental health status of the participant. This is a possible scenario in all treatment interventions, but with emotionally unstable youths there is a need for a robust patient safety management program. We have structured this as follows: During the entire treatment period, the adolescents will be under continuous monitoring by the research group and the main project investigator. At inclusion, an experienced clinician will assess suicide risk. During this screening, the severity of the self-harm and suicidal ideation is carefully assessed. High-risk patients will be excluded from the study and receive treatment recommendation and referral if needed. An individual crisis plan will be developed for all participants so that the family has both verbal and written information about what they should do in case of deterioration in mental health (e.g. increased suicidal ideation). Participant can also access the individual crisis plan using the mobile app. During treatment, the severity of self-harm and suicidal ideation will be continuously monitored (using the mobile app and internet-administered instruments), ensuring that sudden deterioration in mental health is detected and additional care is provided. It is worth pointing out that this is not common practice in usual child and adolescent health care, since the patient usually meets with his/her therapist only once a week and does not have contact in between sessions. During internet-delivered treatment, the participant as well as the parents have the possibility to contact the therapist at any time over the internet. If necessary, telephone contact and/or personal visits will be arranged. Thus, in comparison to treatment as usual only, the intensive surveillance of the patients in the present study could even be argued to lead to increased patient safety.

Inactive participants: Our experience from other studies is that those who manage to actively engage in treatment also benefit from them. Inactivity may thus lead to lack of effect. In case participants

stop/or never starts communicating through the platform, the therapist will contact the parents and agree on future arrangements (i.e. receive treatment recommendation and referral if needed).

Extra burden in terms of repeated assessments: Before the treatment starts the participant is thoroughly assessed through clinical assessment as well as self-report measures. This can imply an extra burden for the participant, and may be perceived as demanding. However, participating in a study like this one can also mean that the adolescent is granted access to a detailed and professional assessment and self-report measures. This allows participants the opportunity to report self-destructive behaviors in a non-stigmatizing way. It also gives access to a modern, promising cognitive behavioral treatment with an explicit rationale, i.e. there is an open dialogue regarding the techniques that the therapists are using and why. This should outweigh the negative impact that participation in the study might be associated with.

Delayed treatment start: Given the design of this study (with an active control group), half of the participants will be asked to initiate the iERITA treatment 24 weeks after the face-to-face assessment. This could possibly result in drop-outs, missing the participation in a possibly effective treatment (i.e. the addition of iERITA), due to being randomized to the control condition. Therefore we refer, recommend and encourage all participants, in both conditions, to participate in the best available treatment as usual. If a participant does not have an ongoing treatment/counselling, the receiving team will assist in helping the initiation of adequate contact. Further, the length of the TAU condition is kept to the minimum (three months after the intervention group has completed treatment) and all participants will be offered iERITA.

Benefits: Participants and their families can benefit from participation in the study. All participants are thoroughly assessed by professional and experienced clinicians. Many adolescents with self-harm behaviors have benefited in earlier studies from interventions aiming at increasing emotion regulation skills.[62] In these studies, serious symptoms and suffering, both for the participant and their families, have decreased. This is also evident in the ongoing pilot study of iERITA. Internet-delivered treatment in addition to other ongoing treatment/counselling would mean increased amount of care for the participants, which could benefit improvement. Furthermore, in case of psychiatric deterioration during participation, this could quickly be detected through self-reports and frequent contact, hence adjustments of the care level could be made.

In conclusion, possible risks with this study are estimated to be lower than the possibilities of pronounced benefits for the participants.

SIGNIFICANCE

Repeated NSSI affects about 6.7% of Swedish adolescents[3], is associated with anxiety and depression, and is predictive of suicide attempts, making it a serious issue for the society but especially for the afflicted adolescents and their families. To date, evidence based treatment options are lacking[15] and accessible treatment option for this patient group is urgently needed (SBU, 2015)[64]. A short-term internet-delivered treatment, if shown to be effective, could give greater access to treatment, lower health care costs, and contribute to evidence-based treatments for this group. As this project is of relevance for the “Nationella självskadeprojektet”, which aims to reduce self-harm among adolescents, the research team will interact with this organization to implement and conduct further studies if it demonstrates feasibility and efficacy. Thus, the project has the potential to lead to large benefits for the affected adolescents, their families, and society at large.

REFERENCES

1 Nock MK. Self-Injury. *Annu Rev Clin Psychol* 2010; **6**: 339–63.

2 Ross S, Heath N. A Study of the Frequency of Self-Mutilation in a Community Sample of Adolescents. *J Youth Adolescence* 2002; **31**: 67–77.

3 Zetterqvist M, Lundh L-G, Dahlström Ö, Svedin CG. Prevalence and Function of Non-Suicidal Self-Injury (NSSI) in a Community Sample of Adolescents, Using Suggested DSM-5 Criteria for a Potential NSSI Disorder. *J Abnorm Child Psychol* 2013; **41**: 759–73.

4 Lloyd-Richardson EE, Lewis SP, Whitlock JL, Rodham K, Schatten HT. Research with adolescents who engage in non-suicidal self-injury: ethical considerations and challenges. *Child Adolesc Psychiatry Ment Health* 2015; : 1–14.

5 Klonsky ED. Non-suicidal self-injury in United States adults: prevalence, sociodemographics, topography and functions. *Psychol Med* 2011; **41**: 1981–6.

6 Nock M, Joinerjr T, Lloyd-Richardson E, Gordon K, Prinstein MJ. Non-suicidal self-injury among adolescents: Diagnostic correlates and relation to suicide attempts. *Psychiat Res* 2006; **144**: 65–72.

7 Jacobson CM, Muehlenkamp JJ, Miller AL, Turner JB. Psychiatric Impairment Among Adolescents Engaging in Different Types of Deliberate Self-Harm. *J Clin Child Adolesc Psychol* 2008; **37**: 363–75.

8 Glenn CR, Klonsky ED. Nonsuicidal Self-Injury Disorder: An Empirical Investigation in Adolescent Psychiatric Patients. *J Clin Child Adolesc Psychol* 2013; **42**: 496–507.

9 American Psychiatric Association. Diagnostic and statistical manual of mental disorders, 5 edn. Washington, DC: Author, 2013.

10 Andover MS, Gibb BE. Non-suicidal self-injury, attempted suicide, and suicidal intent among psychiatric inpatients. *Psychiat Res* 2010; **178**: 101–5.

11 Asarnow JR, Porta G, Spirito A, *et al.* Suicide attempts and nonsuicidal self-injury in the treatment of resistant depression in adolescents: findings from the TORDIA study. *J Am Acad Child Adolesc Psychiatry* 2011; **50**: 772–81.

12 Wilkinson P, Kelvin R, Roberts C, Dubicka B, Goodyer I. Clinical and psychosocial predictors of suicide attempts and nonsuicidal self-injury in the Adolescent Depression Antidepressants and Psychotherapy Trial (ADAPT). *Am J Psychiatry* 2011; **168**: 495–501.

13 Nock MK, Borges G, Bromet EJ, Cha CB, Kessler RC, Lee S. Suicide and Suicidal Behavior. 2008; **30**: 133–54.

14 Hamza CA, Stewart SL, Willoughby T. Examining the link between nonsuicidal self-injury and suicidal behavior: A review of the literature and an integrated model. *Clin Psychol Rev* 2012; **32**: 482–95.

15 Kendall T, Taylor C, Bhatti H, Chan M, Kapur N, Guideline Development Group of the National Institute for Health and Clinical Excellence. Longer term management of self harm: summary of NICE guidance. BMJ. 2011; **343**: d7073–3.

16 Whitlock J, Eckenrode J, Silverman D. Self-injurious behaviors in a college population. 2006; **117**: 1939–48.

17 Washburn JJ, Gebhardt M, Styer DM, Juzwin KR, Gottlieb L. Co-Occurring Disorders in the Treatment of Nonsuicidal Self-Injury: An Evidence-Informed Approach. *J Cogn Psychother* 2012; **26**: 348–64.

18 Linehan M. Cognitive-behavioral treatment of borderline personality disorder. New York: Guilford press, 1993.

19 Stoffers JM, Völlm BA, Rücker G, Timmer A, Huband N, Lieb K. Psychological therapies for people with borderline personality disorder. *Cochrane Database Syst Rev* 2012; **8**: CD005652.

20 Kliem S, Kröger C, Kosfelder J. Dialectical behavior therapy for borderline personality disorder: A meta-analysis using mixed-effects modeling. *J Consult Psychol* 2010; **78**: 936–51.

21 Zanarini MC. Psychotherapy of borderline personality disorder. *Acta Psychiatrica Scandinavica* 2009; **120**: 373–7.

22 Gratz KL, Gunderson JG. Preliminary data on an acceptance-based emotion regulation group intervention for deliberate self-harm among women with borderline personality disorder. *Behav Ther* 2006; **37**: 25–35.

23 Gratz KL, Bardeen JR, Levy R, Dixon-Gordon KL, Tull MT. Mechanisms of change in an emotion regulation group therapy for deliberate self-harm among women with borderline personality disorder. *Behav Res Ther* 2015; **65**: 29–35.

24 Gratz KL, Roemer L. The relationship between emotion dysregulation and deliberate self-harm among female undergraduate students at an urban commuter university. 2008; **37**: 14–25.

25 Gratz KL, Tull MT, Levy R. Randomized controlled trial and uncontrolled 9-month follow-up of an adjunctive emotion regulation group therapy for deliberate self-harm among women with borderline personality disorder. *Psychol Med* 2014; **44**: 2099–112.

26 Gratz KL, Tull MT. Extending research on the utility of an adjunctive emotion regulation group therapy for deliberate self-harm among women with borderline personality pathology. *Personal Disord* 2011; **2**: 316–26.

27 Sahlin H, Bjureberg J, Gratz KL, *et al.* (in press). Emotion Regulation Group Therapy for deliberate self-harm: A multi-site effectiveness study. *BMJ Open*.

28 Bjureberg J, Sahlin H, Gumpert CH, *et al.* 6.152 Emotion Regulation Individual Therapy Administered Face-To-Face For Adolescents With Nonsuicidal Self-Injury Disorder: An Open Pilot Study. *J Am Acad Child Adolesc Psychiatry* 2016; **55**: S252.

29 Andrews G, Cuijpers P, Craske MG, McEvoy P, Titov N. Computer Therapy for the Anxiety and Depressive Disorders Is Effective, Acceptable and Practical Health Care: A Meta-Analysis. *PLoS ONE* 2010; **5**: e13196.

30 Andersson G. Internet-Delivered Psychological Treatments. *Annu Rev Clin Psychol* 2016; **12**: 157–79.

31 Ebert DD, Zarski A-C, Christensen H, *et al.* Internet and Computer-Based Cognitive Behavioral Therapy for Anxiety and Depression in Youth: A Meta-Analysis of Randomized Controlled Outcome Trials. *PLoS ONE* 2015; **10**: e0119895–15.

32 Cuijpers P, Donker T, van Straten A, Li J, Andersson G. Is guided self-help as effective as face-to-face psychotherapy for depression and anxiety disorders? A systematic review and meta-analysis of comparative outcome studies. *Psychol Med* 2010; **40**: 1943–57.

33 Andersson G, Cuijpers P, Carlbring P, Riper H, Hedman E. Guided Internet-based vs. face-to-face cognitive behavior therapy for psychiatric and somatic disorders: a systematic review and meta-analysis. *World Psychiatry* 2014; **13**: 288–95.

34 Berger M, Wagner TH, Baker LC. Internet use and stigmatized illness. 2005; **61**: 1821–7.

35 Gratz KL, Dixon-Gordon KL, Chapman AL, Tull MT. Diagnosis and Characterization of DSM-5 Nonsuicidal Self-Injury Disorder Using the Clinician-Administered Nonsuicidal Self-Injury Disorder Index. *Assessment* 2015; **22**: 527–39.

36 Sheehan DV, Sheehan KH, Shytle RD, *et al.* Reliability and validity of the Mini International Neuropsychiatric Interview for Children and Adolescents (MINI-KID). *J Clin Psychiatry* 2010; **71**: 313–26.

37 Phillips KA. Understanding Body Dysmorphic Disorder: an Essential Guide. New York: Oxford University Press, 2009.

38 Hansson SL, Röjvall AS, Rastam M, Gillberg C. Psychiatric telephone interview with parents for screening of childhood autism–tics, attention-deficit hyperactivity disorder and other comorbidities (A–TAC). *Br J Psychiatry* 2005; **187**: 262–7.

39 First MB, Gibbon M, Spitzer RL, Williams JB, Benjamin L. Structured clinical interview for DSM-IV personality disorders (SCID-II): Interview and questionnaire. *APA, Washington, DC* 1997.

40 Shaffer D, Gould MS, Brasic J, *et al.* A children's global assessment scale (CGAS). *Arch Gen Psychiatry* 1983; **40**: 1228–31.

41 National Institute of Mental Health. Rating scales and assessment instruments for use in pediatric psychopharmacology research. *Psychopharmacol Bull* 1985; **21**: 839–43.

42 Guy W. Assessment manual for psychopharmacology, revised (DHEW Publication ABM 76–366). *Washington, DC: US Government Printing Office* 1976.

43 Gratz KL, Latzman RD, Young J, *et al.* Deliberate self-harm among underserved adolescents: The moderating roles of gender, race, and school-level and association with borderline personality features. *Personal Disord* 2012; **3**: 39–54.

44 Gratz KL, Roemer L. Multidimensional Assessment of Emotion Regulation and Dysregulation: Development, Factor Structure, and Initial Validation of the Difficulties in Emotion Regulation Scale. *J Psychopathol Behav Assess* 2004; **26**: 41–54.

45 Bjureberg J, Ljótsson B, Tull MT, *et al.* Development and Validation of a Brief Version of the Difficulties in Emotion Regulation Scale: The DERS-16. *J Psychopathol Behav Assess* 2015; : 1–13.

46 Bohus M, Limberger MF, Frank U, Sender I, Gratwohl T, Stieglitz RD. [Development of the Borderline Symptom List]. *Psychother Psychosom Med Psychol* 2001; **51**: 201–11.

47 Crick NR, Murray-Close D, Woods K. Borderline personality features in childhood: a short-term longitudinal study. *Dev Psychopathol* 2005; **17**: 1051–70.

48 Lovibond PF, Lovibond SH. The structure of negative emotional states: comparison of the Depression Anxiety Stress Scales (DASS) with the Beck Depression and Anxiety Inventories. *Behav Res Ther* 1995; **33**: 335–43.

49 Reynolds WM, Mazza JJ. Assessment of suicidal ideation in inner-city children and young adolescents: Reliability and validity of the suicide ideation questionnaire-JR. *School Psychology Review* 1999; **28**: 17–30.

50 Ravens-Sieberer U, Erhart M, *et al.* Reliability, construct and criterion validity of the KIDSCREEN-10 score: a short measure for children and adolescents' well-being and health-related quality of life. *Qual Life Res* 2010; **19**: 1487–500.

51 Bond FW, Hayes SC, Baer RA, *et al.* Preliminary Psychometric Properties of the Acceptance and Action Questionnaire–II: A Revised Measure of Psychological Inflexibility and Experiential Avoidance. 2011; **42**: 676–88.

52 Brenning K, Van Petegem S, Vanhalst J, Soenens B. The psychometric qualities of a short version of the Experiences in Close Relationships Scale â€“ Revised Child version. *Personality And Individual Differences* 2014; **68**: 118–23.

53 Bastien CH, Vallières A, Morin CM. Validation of the Insomnia Severity Index as an outcome measure for insomnia research. *Sleep Medicine* 2001; **2** :297-307.*)*

54 Falkenstr m F, Hatcher RL, Skjulsvik T, Larsson MH, Holmqvist R. Development and validation of a 6-item working alliance questionnaire for repeated administrations during psychotherapy. *Psychological Assessment* 2015; **27**: 169–83.

55 Devilly GJ, Borkovec TD. Psychometric properties of the credibility/expectancy questionnaire. *J Behav Ther Exp Psychiatry* 2000; **31**: 73–86.

56 Attkisson CC, Zwick R. The client satisfaction questionnaire. Psychometric properties and correlations with service utilization and psychotherapy outcome. *Eval Program Plann* 1982; **5**: 233–7.

57 Andersson E. (Unpublished manuscript). Adverse Event Protocol. Institute for Clinical Neuroscience. Karolinska institutet.

58 Lenhard F. (Unpublished manuscript). The clinician rated ICBT adherence scale. Institute for Clinical Neuroscience. Karolinska institutet.

59 Nock MK, Holmberg EB, Photos VI, Michel BD. Self-Injurious Thoughts and Behaviors Interview: development, reliability, and validity in an adolescent sample. *Psychological Assessment* 2007; **19**: 309–17.

60 Fabes RA, Eisenberger N. (Unpublished manuscript). The Coping with Children's Negative Emotions Scale Adolescent Version: Procedures and Scoring. 1998.

61 L Haakart-van Roijen. Manual Trimbos/iMTA questionnaire for costs associated with psychiatric illness. Rotterdam: Institute for Medical Technology Assessment; 2002.

62 Gratz KL, Tull MT, Levy R. Randomized controlled trial and uncontrolled 9-month follow-up of an adjunctive emotion regulation group therapy for deliberate self-harm among women with borderline personality disorder. *Psychol Med* 2013; **44**: 2099–112.

63 Washburn JJ, Richardt SL, Styer DM, *et al.* Psychotherapeutic approaches to non-suicidal self-injury in adolescents. *Child Adolesc Psychiatry Ment Health* 2012; **6**: 14.

64 SBU, Statens beredning för medicinsk och social utvärdering (SBU); Swedish Agency for Health Technology Assessment and Assessment of Social Services (SBU). Skolbaserade program för att förebygga självskadebeteende inklusive suicidförsök. 2015: 1–72.

Power and Sample Size

A Randomized Controlled Trial of Internet-delivered Emotion Regulation Individual Treatment for Adolescents with Non-suicidal Self-injury

Professor Matteo Bottai

September 15, 2017

Figure 1. Histogram of episodes8 (yellow bars) and predicted probabilities from a zero-inflated negative binomial model (red dots)

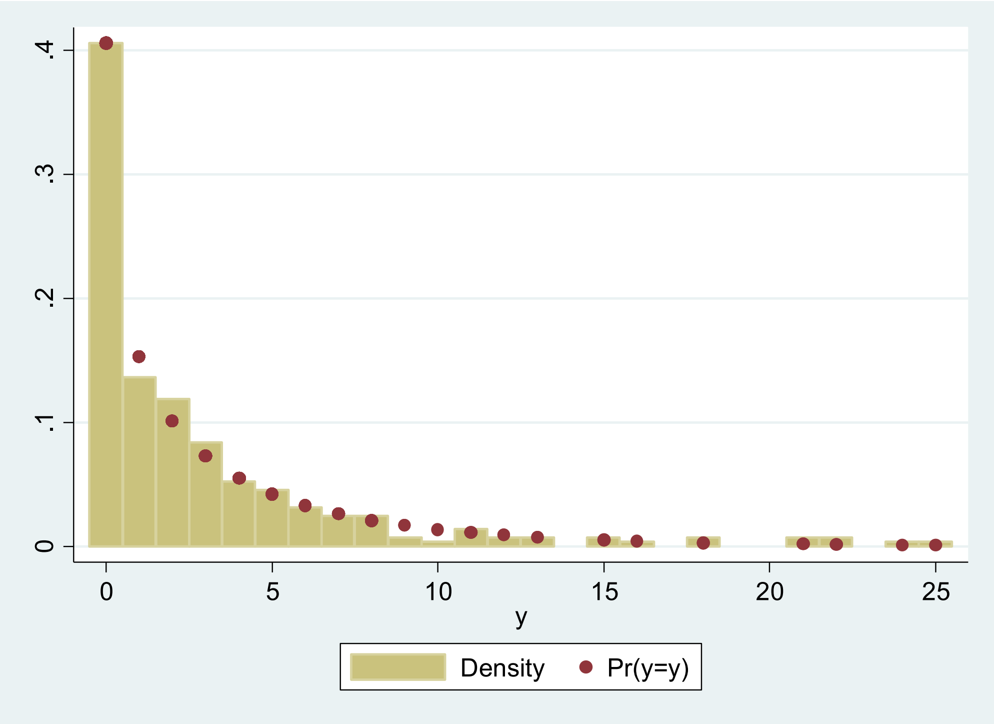

Figure 2. Box plots of the distribution of episodes8 over time in weeks

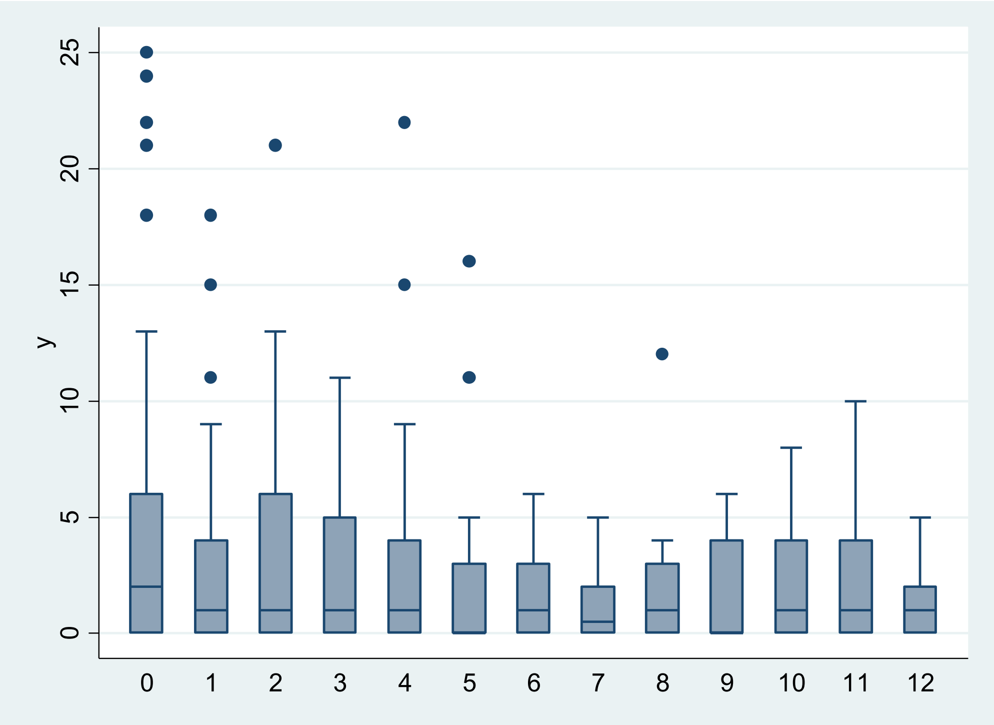

Figure 3. Estimated trend of the rate of episodes8 over time with a zero-inflated negative binomial model.

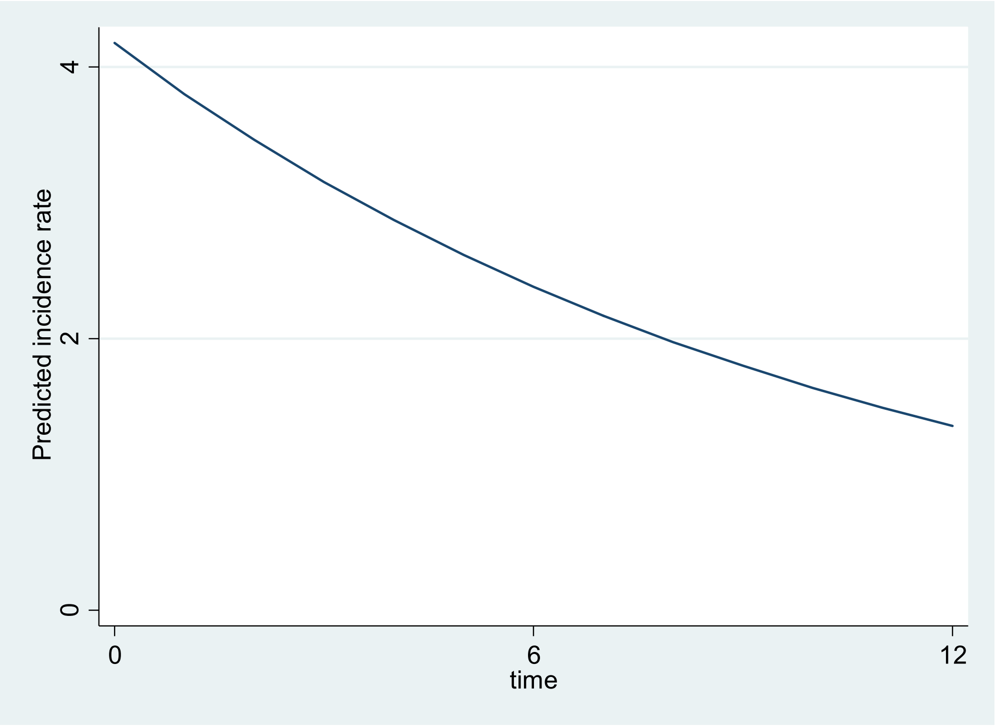

Table 1. Estimated power to detect a different slope between the treated and control groups corresponding to different sample sizes and differences in the expected count between groups after the 12-week follow-up. The power refers to a 0.05-level Wald test for the interaction between a binary treatment and linear time in a zero-inflated negative binomial regression model at a 0.05 level. The power was estimated with 200 bootstrap samples. The data for the treated group were obtained by sampling with replacement from available pilot data from an earlier study on 25 treated patients. The data for the control group was obtained by adding the difference specified in the table to the count of a bootstrap sample from the pilot data on the 25 treated patients.

Difference between groups in average count after 12 weeks	Sample size per group	Estimated power
1	150	0.80
2	70	0.82
