# Supplementary material for: Effect of Internet-Delivered Emotion Regulation Individual Therapy for Adolescents With Nonsuicidal Self-Injury Disorder: A Randomized Clinical Trial
Source: JAMA Netw Open. 2023 Jul 13;6(7):e2322069. doi: 10.1001/jamanetworkopen.2023.22069 (PMC10346121; doi:10.1001/jamanetworkopen.2023.22069)
Supplement: Supplement 3. — Data Sharing Statement [file jamanetwopen-e2322069-s003.pdf]

Data Sharing Statement

Bjureberg. Effect of Internet-Delivered Emotion Regulation Individual Therapy for Adolescents With Nonsuicidal Self-Injury Disorder. *JAMA Netw Open*. Published July 13, 2023. doi:10.1001/jamanetworkopen.2023.22069

Data
Data available: No

Additional Information
Explanation for why data not available: Patient-level data are not publicly available due to national (Swedish) and EU legislation but could be made available from the corresponding author upon reasonable request following approval from the Swedish Ethical Review Authority.
